# Discovery and Validation of a Novel Class of Necroptosis Inhibitors Targeting RIPK1

**DOI:** 10.1021/acschembio.5c00112

**Published:** 2025-06-20

**Authors:** Lior Soday, Chotima Seripracharat, Janine L. Gray, André F. S. Luz, Ryan T. Howard, Ravi Singh, Thomas J. Burden, Erika Bernardini, Miguel Mateus-Pinheiro, Jens Petersen, Anders Gunnarsson, Jenny Gunnarsson, Anna Aagaard, Tove Sjögren, Sarah Maslen, Edward J. Bartlett, Abigail F. Iles, David M. Smith, James S. Scott, Mark Skehel, Andrew M. Davis, Ana S. Ressurreição, Rui Moreira, Cecília M. P. Rodrigues, Avinash R. Shenoy, Edward W. Tate

**Affiliations:** † Department of Chemistry, Molecular Sciences Research Hub, 4615 Imperial College London, London W12 0BZ, U.K.; ‡ Research Institute for Medicines (iMed.ULisboa), Faculty of Pharmacy, Universidade de Lisboa, Lisbon 1649-004, Portugal; § Discovery Sciences, R&D Gothenburg, AstraZeneca, Pepparedsleden 1, SE-431 83 Mölndal, Sweden; ∥ 376570 The Francis Crick Institute, London NW1 1AT, U.K.; ⊥ Hit Discovery, Discovery Sciences, R&D, 4625 AstraZeneca, Cambridge CB2 0AA, U.K.; # Department of Infectious Diseases, Imperial College London, Flowers Building, South Kensington Campus, London SW7 2AZ, U.K.

## Abstract

Necroptosis is a form of programmed cell death that, when dysregulated, is associated with cancer and inflammatory and neurodegenerative diseases. Here, starting from hits identified from a phenotypic high-throughput screen for inhibitors of necroptosis, we synthesized a library of compounds containing a 7-phenylquinoline motif and validated their anti-necroptotic activity in a novel live-cell assay. Based on these data, we designed an optimized photoaffinity probe for target engagement studies and through biochemical and cell-based assays established receptor-interacting kinase 1 (RIPK1) as the cellular target, with inhibition of necroptosis arising from the prevention of RIPK1 autophosphorylation and activation. X-ray crystallography and mass spectrometry revealed that these compounds bind at the hinge region of the active conformation of RIPK1, establishing them as type I kinase inhibitors. In addition, we demonstrated *in vitro* synergy with type III kinase inhibitors, such as necrostatin-1 and found that lead compounds protected mice against acute inflammation in necroptosis models *in vivo*. Overall, we present a novel pharmacophore for inhibition of human RIPK1, a key protein involved in necroptosis, and provide a photoaffinity probe to explore RIPK1 target engagement in cells.

## Graphical abstract

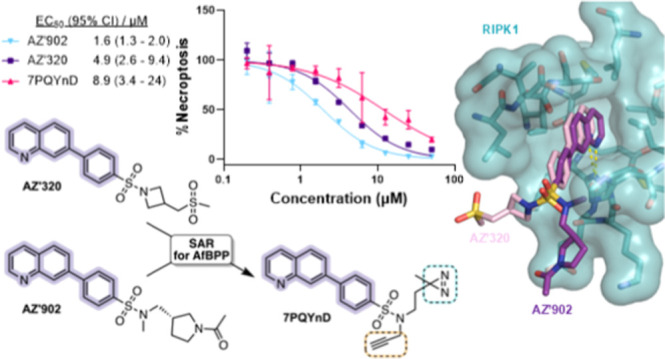

## Introduction

Necroptosis is a form of regulated cell death which occurs in response to inflammatory cytokines or infection with pathogens and therefore plays a critical role in the host immune response and defense against infection.^1,2^ Canonical programmed cell death signaling can be initiated by the binding of tumor necrosis factor alpha (TNF) to its receptor TNFR1 on the cell surface, resulting in NF-kB-dependent cell-survival signaling or caspase-8-dependent apoptosis.^3^ However, in specific cellular contexts, such as the inhibition of caspase-8 or the absence of the signaling adaptor FADD, necroptosis occurs through the formation of the necrosome complex. The necrosome comprises receptor-interacting serine/threonine-protein kinase 1 (RIPK1), RIPK3, and mixed lineage kinase domain-like protein (MLKL).^4,5^ Active RIPK1 phosphorylates and activates RIPK3, which in turn phosphorylates MLKL. Phospho-MLKL oligomerization and translocation to the plasma membrane results in membrane rupture, release of damage-associated molecular patterns (DAMPs), and inflammation, a hallmark of necroptosis.^6–8^

While necroptosis plays an important role in immunity, its dysregulation is associated with inflammatory and autoimmune disorders,^1,2^ ischemic-reperfusion injury,^9^ neurodegenerative diseases,^10–12^ and cancer.^13–15^ The clinical potential of necroptosis inhibitors has led to an array of drug discovery programs primarily targeting components of the necrosome. A range of tool compounds has been developed, including Necrostatin 1 (Nec-1) and its derivatives,^9,16^ which are type III kinase inhibitors that bind to an allosteric pocket of RIPK1, and Necrosulfonamide (NSA),^17^ a covalent inhibitor of MLKL, which was instrumental in elucidating the critical role of the protein in the pathway. Several small molecules are in clinical trials, including RIPK1 inhibitors developed by Denali Therapeutics/Sanofi for inflammatory and neurodegenerative disorders, emphasizing the therapeutic potential of necroptosis inhibition.^18,19^

We previously reported a phenotypic high-throughput screen (HTS) which evaluated the antinecroptotic potential of a diverse library of >250,000 compounds in mouse fibroblast (L929) cells and human FADD^–/–^ acute T-cell lymphoblastic leukemia (Jurkat) cells (I2.1).^20^ Hits were further triaged in counterscreens for RIPK1 or RIPK3 kinase activity, with active compounds removed from the set. The HTS identified 356 compounds that selectively inhibited necroptosis by an unknown mechanism of action, including a series of compounds containing a 7-phenylquinoline (7PQ) motif, which we further explored in this study.

Here, we describe a multifaceted array of molecular, cellular, structural, and chemical biology approaches to characterize the 7PQ inhibitor series and identify RIPK1 as their target. A novel photoaffinity-based probe was designed and synthesized to confirm target engagement, and structures of RIPK1 bound to 7PQ analogues were solved by X-ray crystallography, with the binding mode further confirmed by surface plasmon resonance (SPR) and hydrogen–deuterium exchange (HDX). Notably, the 7PQ compounds bound RIPK1 at the hinge region, a site distinct from Nec-1, and cell-based experiments demonstrated functional synergy between these classes of inhibitors. Finally, we report *in vivo* activity in mouse models of acute inflammation comparable to that of one of the most potent known necroptosis inhibitors, providing a validated and novel chemotype for this important area of drug discovery.

## Results

### Identification of the 7-Phenylquinoline Pharmacophore Series as Novel Necroptosis Inhibitors

All compounds in the series identified in the HTS share the 7PQ motif (LHS) but bear diverse *para* substitutions at the phenyl group linked by a sulfonamide or amide (RHS).^20^ These compounds potently inhibited necroptosis via an unknown mechanism of action; **AZ’902** and **AZ’320** were selected as representatives for further investigation and target identification (Figure 1a).

**Figure 1:**
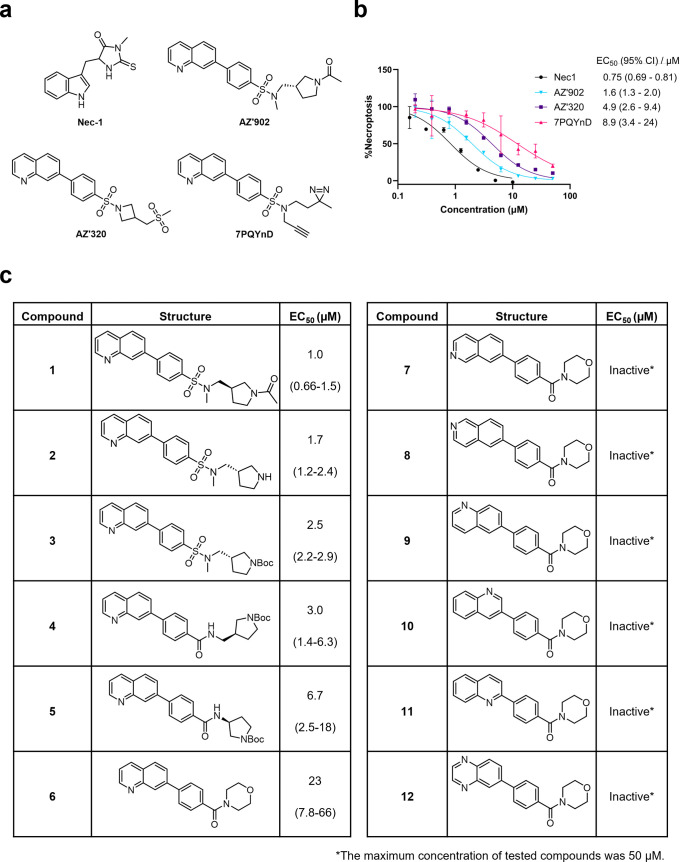
Structure–activity relationship (SAR) studies of a novel series of necroptosis inhibitors. (a) Structures of necroptosis inhibitors Nec-1, **AZ’902**, and **AZ’320** and the photoaffinity-based probe **7PQYnD** from the 7PQ compound series. (b) Dose–response curves for Nec-1, **AZ’902**, **AZ’320**, and **7PQYnD** in I.21 FADD^–/–^ human Jurkat cells. Results are representative of at least three independent repeats; error bars represent SD (*n* ≥ 3). (c) Structures and EC_50_ values (95% confidence interval (CI)) for compounds within the 7PQ SAR series. EC_50_ values in (b,c) are expressed as the geometric mean with a 95% CI of at least three biological replicates.

A cell-based assay was established to monitor necroptosis in I2.1 cells using the IncuCyte S3 live cell microscopy platform (Figure S1a). Necroptosis was induced by treatment with TNF,^21^ and Sytox Green was used to monitor membrane permeabilization over time as an indicator of progressive necroptotic cell death (Figure S1b–d), allowing determination of the EC_50_ of candidate necroptosis inhibitors. This novel assay was validated using well-characterized necroptosis inhibitors Nec-1s,^9,16^ GSK’872,^22,23^ and NSA^17^ (inhibitors of RIPK1, RIPK3, and MLKL, respectively), with EC_50_ values consistent with those previously reported (Figure S1e). Necroptosis inhibition by **AZ’902** and **AZ’320** was confirmed in I2.1 cells, with low micromolar EC_50_ (1.6 and 4.9 μM) in a range similar to that of the archetypal RIPK1 inhibitor Nec-1 (0.75 μM) (Figure 1b). The activity of **AZ’902** was further confirmed in HT-29 cells, a type of colonic epithelial cell line, as a second cell-based model of necroptosis (Figure S1f).^24^

A series of 7PQ analogues was synthesized to explore the SAR for necroptosis inhibition in the IncuCyte assay (Figure 1c). Considering the two key structural motifs in these compounds, we first explored the RHS group, retaining the 7PQ motif. The enantiomer (**1**) showed comparable activity to **AZ’902**, indicating no chirality preference in this region of the molecule. Removal of the acetyl group (**2**) or replacement with a larger carbamate moiety (**3**) was also well-tolerated. Substitution of the sulfonamide with an amide (**4**) and shortening the linker of the amide nitrogen substituent (**5**) demonstrated little change in activity, suggesting limited interaction of the RHS with the target and indicating a potential site for modification in development of a photocrosslinkable affinity-based probe for target engagement studies.^25^

We next installed a simplified amide motif (**6**) to facilitate rapid derivatization of analogues varying the 7PQ motif. Quinolinyl-nitrogen was then placed at different positions (**7**–**11**), and we found that any position other than the parental analogue exhibited a loss of inhibition, suggesting a critical role for the position of the nitrogen in the 7PQ ring. Replacement with a quinoxaline ring (**12**) also resulted in a loss of activity. The electron-withdrawing effect of the additional nitrogen is expected to reduce the basicity of the ring, further supporting a key role for the nitrogen lone pair.

We next designed photoaffinity probe **7PQYnD** (Figure 1a), incorporating a diazirine photoreactive warhead along with a terminal alkyne ligation handle appended to the 7PQ-sulfonamide scaffold. As anticipated based on our SAR data, **7PQYnD** displayed necroptosis inhibition activity, albeit ∼2–5-fold less potent than the parent compounds **AZ’902** and **AZ’320**, but was deemed sufficiently potent for progression to cellular target engagement studies.

### **AZ’902** and **AZ’320** Inhibit Receptor-Interacting Kinase 1 Phosphorylation

Formation of the necrosome involves RIPK1 autophosphorylation at multiple sites, followed by phosphorylation of RIPK3, and finally MLKL phosphorylation,^7^ which initiates pore formation and necroptosis. The phosphorylation state of RIPK1 Ser166, which is highly correlated with RIPK1 activity and autophosphorylation,^26^ was monitored to further investigate the mechanism of necroptosis in I2.1 cells. Induction of necroptosis by TNF in I2.1 cells resulted in phosphorylation of RIPK1 Ser166, as expected, and was blocked by prior treatment with 10 μM **AZ’902** or **7PQYnD**, or 10 μM RIPK1 inhibitor Nec-1 (Figure 2a). **7PQYnD** less efficiently inhibited RIPK1 phosphorylation compared to **AZ’902**, consistent with the lower potency of the photoprobe, whereas the RIPK3 inhibitor GSK’872 showed no inhibition of RIPK1 Ser166 phosphorylation, as expected. Furthermore, the effect of **AZ’902** and **AZ’320** on phosphorylation of RIPK1 Ser166 was dose dependent, suggesting a pharmacological mode of action at RIPK1 (Figure 2b). These results were recapitulated in HT-29 cells; here, necroptosis was induced by treatment with TNF, birinapant,^27^ and carbobenzoxy-valyl-alanyl-aspartyl-[O-methyl]-fluoromethylketone (Z-VAD-FMK). Pretreatment with 7PQ compounds showed a dose-dependent effect on RIPK1 Ser166 phosphorylation, while GSK’872 inhibited phosphorylation of RIPK3 and MLKL but not RIPK1, and NSA retained phosphorylation of all components of the necrosome, consistent with the mode of action of these compounds (Figure S2a,b). Taken together, these data suggested that inhibition of necroptosis by **AZ’902** and **AZ’320** is mediated by inhibition of RIPK1 phosphorylation.

**Figure 2:**
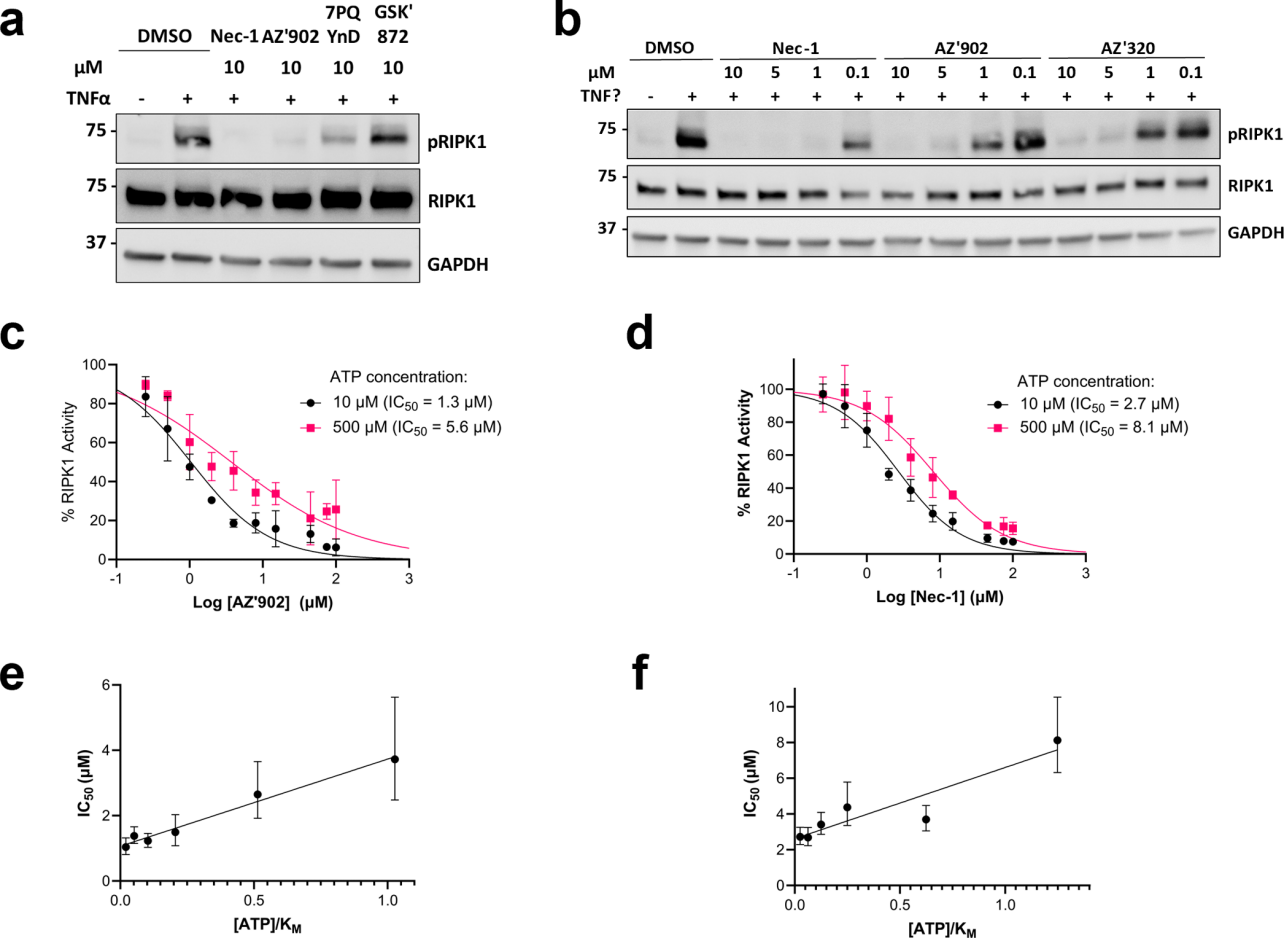
Validation of the 7PQ series as inhibitors of RIPK1. (a,b) Western blot analysis of RIPK1 Ser166 phosphorylation in I2.1 cells with treatment of necroptosis inhibitors and the 7PQ series. Results are representative of two experiments. GAPDH was used as a loading control. Uncropped immunoblots associated with all figures can be found in Figure S5. (c–f) ADP-Glo kinase assay to determine **AZ’902** and Nec-1 inhibition of RIPK1 kinase activity with increasing concentrations of ATP. (c–d) Dose response curves for **AZ’902** (c) and Nec-1 (d) with ATP concentrations of 10 μM and 500 μM. (e,f) IC_50_ values of **AZ’902** (e) and Nec-1 (f) as a function of ATP concentration divided by the Km for RIPK1. Experiments were carried out with **AZ’902** or Nec-1 at ten concentrations (0–100 μM), varied ATP concentrations (10–500 μM), and constant GST-RIPK1 concentration (75 nM). Data are shown as means ± SD (for c,d) and as IC_50_ ± 95% CI (for e,f). Experiments were conducted in triplicate.

Given the precedence for inhibition of RIPK1 to prevent necroptosis, the possibility of direct RIPK1 inhibition was tested in a biochemical ADP-Glo kinase assay using the recombinant RIPK1 kinase domain. Both **AZ’902** and Nec-1 inhibited RIPK1 kinase activity with comparable IC_50_s (Figure 2c,d), confirming the 7PQ series as inhibitors of RIPK1. Investigation at a range of ATP concentrations (10–500 μM) revealed that both compounds showed competition with ATP (Figure 2e,f). To establish the selectivity of the 7PQ compounds, **AZ’902** and **AZ’320** were tested alongside Nec-1 against a panel of 25 kinases, with off–target activity observed primarily against Aurora kinase B, a cell cycle kinase with no known role in necroptosis (Figure S2c). These data suggest that 7PQ anti-necroptotic activity is likely to be mediated directly through RIPK1.

### 7-Phenylquinoline Compounds Bind Receptor-Interacting Kinase 1 at a Binding Site Distinct from Necrostatin 1

Further confirmation of target engagement was established using photoaffinity probe **7PQYnD** (Figure 1a). Following incubation of I2.1 cells with the probe, irradiation to induce photocrosslinking and bioorthogonal ligation to azide-TAMRA-biotin (AzTB^28^) using copper­(I)-catalyzed azide–alkyne cycloaddition, labeling of proteins could be visualized by in-gel fluorescence (Figure S3a). Target engagement was confirmed by Streptavidin mass shift assay,^29^ whereby lysates are incubated with streptavidin and subsequently separated by SDS–PAGE. The high stability of streptavidin at a range of temperatures, in various detergents and pH, in combination with the extremely high affinity for biotin means the streptavidin–biotin interaction is not separated during gel electrophoresis, resulting in a mass shift of biotin-labeled proteins in the presence of streptavidin, which can be visualized by Western blot (Figure 3a). Dose-dependent engagement of RIPK1 was observed as a stronger band at the streptavidin-shifted molecular weight at higher concentrations of probe, particularly after 5 min of UV irradiation (Figure 3b). A competition assay was employed to confirm binding of the parent compounds to RIPK1, whereby the treatment of cells with **AZ’902** prior to photoaffinity labeling with **7PQYnD** eliminated the observed RIPK1 band shift (Figure 3c). However, the **7PQYnD**-mediated streptavidin band shift was retained on pretreatment with Nec-1, indicating that 7PQ compounds likely bind at a site distinct from that of Nec-1 (Figure 3d).

**Figure 3:**
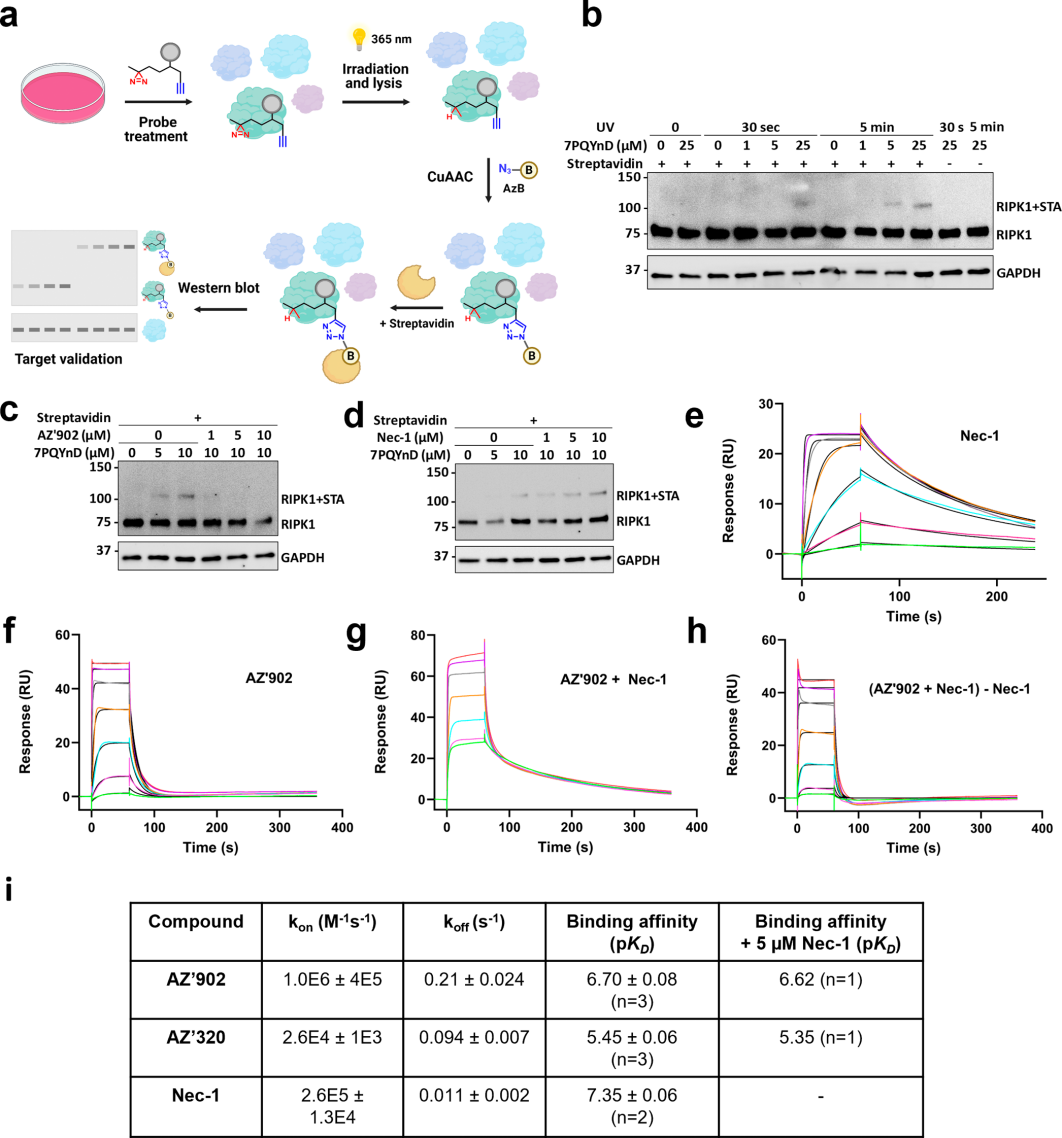
7PQ series binds to the kinase domain of RIPK1. (a) Schematic of the streptavidin mass shift assay workflow used for target engagement studies in I2.1 cells. (b–d) Western blot analysis of the streptavidin mass shift assay in I2.1 cells. Streptavidin is abbreviated as STA. GAPDH was used as a loading control. (b) **7PQYnD** probe used alone. (c) Competition assay where **AZ’902** was preincubated before **7PQYnD** was added to cells. (d) Preincubation of Nec-1 does not impact **7PQYnD** binding to RIPK1. (e–h) SPR representative sensorgrams of increasing concentrations of **AZ’902** and Nec-1 (green, pink, teal, gray, orange, purple) and 1:1 fitting (black) of the interactions with the kinase domain of RIPK1. (i) SPR fitted values of **AZ’320** and **AZ’902** in the presence and absence of Nec-1.

SPR was used to further validate the binding of the compounds to the kinase domain of RIPK1. The p*K*_D_ (−log_10_(*K*_D_)) values of **AZ’902** and **AZ’320** were 6.70 and 5.45, respectively, making them marginally less potent binders than Nec-1 (p*K*_D_ 7.35) (Figures 3e,f and S3b). Furthermore, the binding of 7PQ compounds in the presence of Nec-1 resulted in a shift of the sensorgram, corresponding to the additive binding of these compounds to RIPK1 (Figures 3g and S3c). Subtraction of the Nec-1 signal returned equivalent sensorgrams to the 7PQ compounds alone and demonstrated no alteration of the p*K*_D_ in the presence of Nec-1 (Figures 3h,i and S3d). This further supports binding of **AZ’902** and **AZ’320** to a distinct site on RIPK1, compared to that of Nec-1.

### 7-Phenylquinoline Compounds Bind Receptor-Interacting Kinase 1 at the Hinge Region

To determine the binding mode of 7PQ compounds, we solved and refined X-ray crystal structures of **AZ’902** (PDB code: 9GTG) and **AZ’320** (PDB code: 9GTY) bound to RIPK1 to 2.25 Å and 2.15 Å, respectively (Figure 4a). RIPK1 was purified and crystallized in the presence of Nec-1s followed by soaking with **AZ’902** or **AZ’320**. Nec-1s is an allosteric inhibitor of RIPK1, and binding was observed at a hydrophobic pocket behind the ATP binding site, as previously described.^30^ In contrast, **AZ’902** and **AZ’320** both bound RIPK1 at identical sites around the hinge region, with the 7PQ motif interacting directly with RIPK1 (Figure 4b). A hydrogen bond interaction was identified between the 7PQ nitrogen and the backbone nitrogen of RIPK1 Met95, supporting the critical position of the nitrogen observed in the SAR studies and explaining the inactivity of compounds in which this nitrogen is varied (**7**–**12**) (Figure 1c). The compounds partially overlap with the predicted ATP binding site (Figure S4a) and thus likely act as Type I kinase inhibitors; as per literature, most inhibitors for RIPK1 are Type II or III (Figure S4b).^18^ The RHS of the molecules is exposed to the solvent and does not form notable interactions with RIPK1, consistent with the observation that a range of motifs are tolerated in the *para*-position of the 7-phenyl ring. We were also able to fit electron density to the glycine-rich loop of RIPK1, a region that is often disordered and not well elucidated in previous crystal structures of the protein.

**Figure 4:**
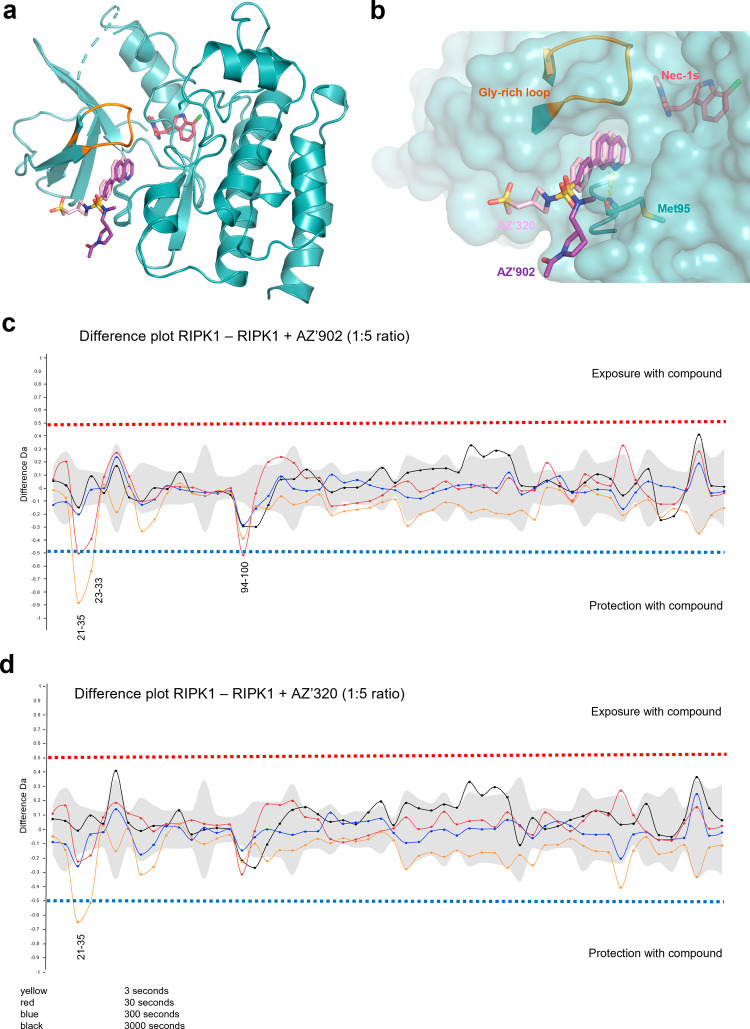
Structural determination of 7PQ binding mode to RIPK1. (a) Co-crystal structure of **AZ’320** (light pink sticks) and Nec-1s (dark pink sticks) bound to RIPK1 (teal cartoon) (PDB = 9GTY). **AZ’902** (purple sticks) overlaid for comparison. The normally disordered glycine-rich loop, which was able to be modeled, is highlighted in orange. (b) Surface representation of the 7PQ binding site, with hydrogen bonds to key residue Met95 shown as yellow dashes. The 7PQ pharmacophore fits into a defined pocket, while the sulfonamide motifs are exposed to solvent. (c,d) HDX-MS experiment representing the differences observed with 7PQ compounds bound to RIPK1 as opposed to RIPK1 alone. The different colored lines represent different incubation times in D_2_O buffer, yellow = 3 s, red = 30 s, blue = 300 s, and black = 3000 s. The experiments were performed in triplicate. (c) HDX-MS difference plot for **AZ’902** bound to RIPK1. The glycine-rich loop (21–35) and hinge region (94–100) are protected from deuterium exchange by the compound. (d) HDX-MS difference plot for **AZ’320** bound to RIPK1. The glycine-rich loop (21–35) is protected from deuterium exchange by the compound.

To explore the binding mode in solution, HDX-mass spectrometry was performed on RIPK1 in the presence or absence of 7PQ compounds. Unbound RIPK1 was used as the reference state, and the difference in deuterium uptake was explored following incubation with **AZ’902** or **AZ’320** (Figure 4c,d); excellent peptide coverage of 91% was obtained (Figure S4c). Both **AZ’902** and **AZ’320** showed protective effects on the glycine-rich loop of RIPK1 (residues 21–35), supporting the stabilization of this region suggested by X-ray crystallography data (Figure S4d). Furthermore, incubation with **AZ’902** led to significant changes in HDX exchange at the hinge region around Met95 (residues 94–100), with the same trend observed for **AZ’320**. Together, these data demonstrate that distinct from Nec-1 analogues, the 7PQ series occupies a hinge region site consistent with data from both SPR analysis and photoaffinity labeling competition studies, and coordinates an ordered state of the glycine-rich loop.

### 7-Phenylquinoline Receptor-Interacting Kinase 1 Inhibitors Synergize with Necrostatin 1 In Vitro and Protect against Acute Inflammation In Vivo

Given the distinct binding sites of the necrostatin and 7PQ series, we investigated the possibility of the synergistic inhibition of necroptosis. The compounds were combined in a checkerboard format, and necroptosis was examined in the IncuCyte assay as previously described. Nec-1s and **AZ’902** acted synergistically specifically in the range of 0.06–0.25 μM Nec-1s and 0.78–3.13 μM **AZ’902**, using the HSA model in the Combenefit analysis package (Figure 5a,b).^31^ Interestingly, synergistic inhibition of necroptosis was observed despite the clear absence of binding synergy in SPR analyses (Figures 3e–h and S3b–d), suggesting an additional layer of functional cooperation between the two compound classes for future investigation.

**Figure 5:**
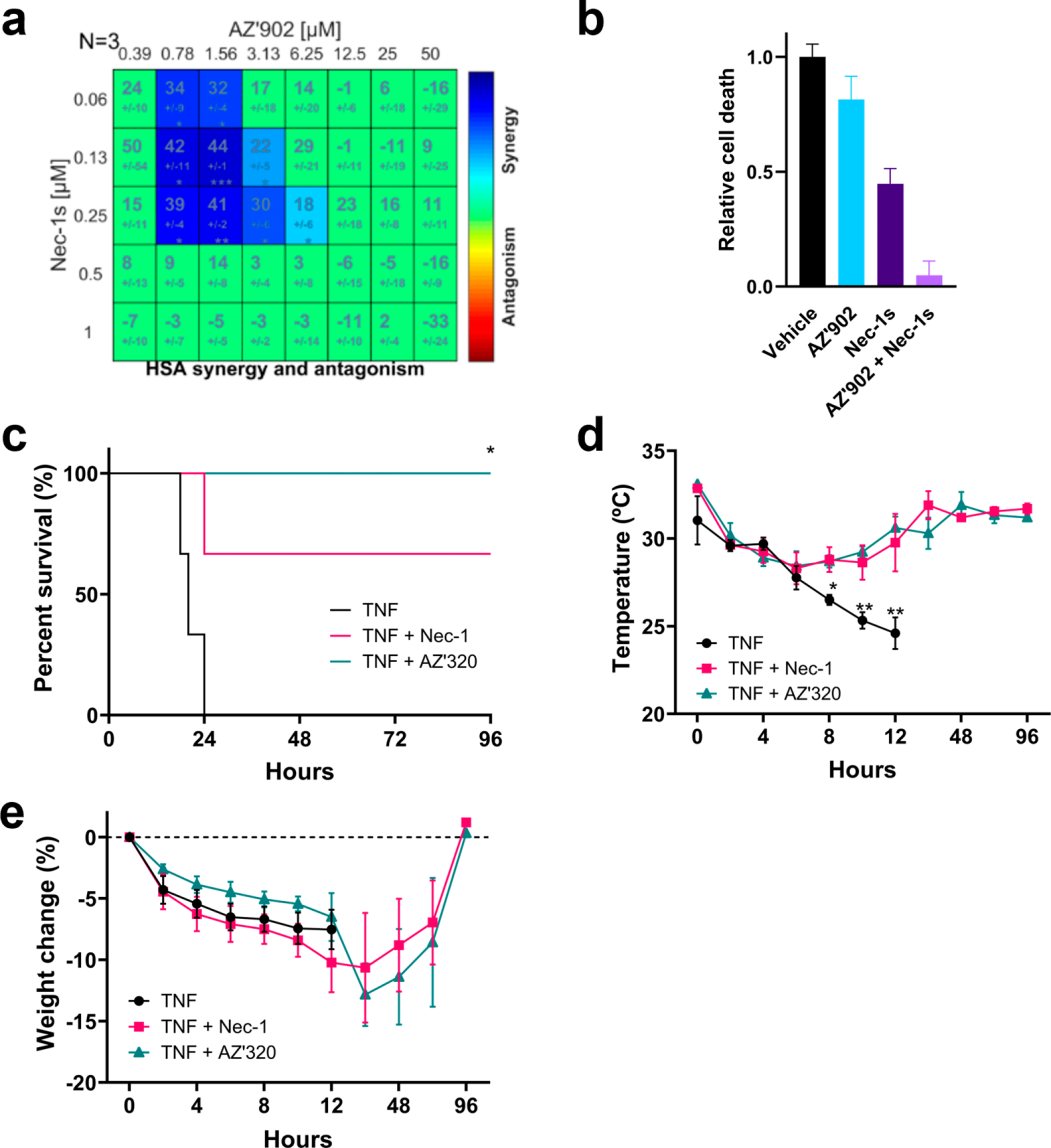
7PQ series synergizes with necrostatins in vitro and exhibits comparable protection to Nec1 against necroptosis-related death in an *in vivo* mouse model for systemic inflammatory response syndrome (SIRS). (a) Synergy/antagonism scores of necroptosis inhibition by Nec-1s and **AZ’902** using the HSA model generated from Combenefit software in I2.1 cells. Blue represents synergism and red represents antagonism. The synergy scores were calculated from three independent biological replicates. (b) Quantification of cell death from the synergy assay. The combination of **AZ’902** (0.78 μM) and Nec-1s (0.25 μM) was more protective than either compound alone or compared to vehicle (DMSO). (c–e) Inhibitory effects of TNF-induced SIRS murine model. 6–8 weeks old C57BL/6J mice were treated intravenously with Nec-1 (5 μg/g) or **AZ’320** (5 μg/g) for 15 min prior to being injected intravenously with TNF (0.5 μg/g). Control mice were injected with TNF and vehicle (44.5% phosphate-buffered saline (PBS) 1×, 17% DMSO, 10% ethanol, 2.5% cyclodextrin, 18% PEG 400, and 8% cremophor). During the first 12 h, mice were frequently monitored for temperature and weight. (c) Survival curve of mice, (d) temperature change of mice, and (e) % body weight change of mice after TNF injection. 24 h after TNF administration, a single injection was lethal in 100% of control mice, accompanied by a reduction in body temperature and weight. Nec-1 partially protected mice from TNF-induced mortality and hypothermia, while **AZ’320** administration completely reverted the toxic effects induced by TNF. Data is expressed as mean ± SEM arbitrary units or percentage of 3 individual mice. *p < 0.5, **p < 0.01.

Finally, given the involvement of necroptosis in inflammatory disorders, the ability of **AZ’320** to prevent mortality in a mouse model of SIRS was investigated.^32^ C57BL/6 mice were injected intravenously (i.v.) with 5 mg/kg Nec-1 or **AZ’320** or vehicle control, followed by mouse TNF (0.5 mg/kg, i.v.) to induce an acute inflammatory response. Treatment with **AZ’320** was found to be protective against SIRS, preventing mortality of the mice (Figure 5c) and rescuing effects on temperature and body weight (Figure 5d,e) to a similar degree as Nec-1.

## Discussion and Conclusions

In this work, we have validated the previously identified 7PQ pharmacophore series as potent inhibitors of necroptosis. We established a novel and robust live-cell imaging assay to monitor necroptosis in real time, which enabled rapid determination of the inhibitory activity of a library of analogues. This established a key role of the heterocyclic nitrogen atom in the 7PQ motif, while the tolerance of sulfonamide substituents instigated development of a photoaffinity probe with the diazirine handle incorporated in this part of the molecule. The **7PQYnD** probe confirmed RIPK1, a key regulatory protein involved in the necrosome, as a cellular target in multiple necroptosis cell models.

An ADP-Glo kinase assay confirmed that the 7PQ series inhibited RIPK1 via the prevention of its kinase autophosphorylation activity. Subsequent streptavidin mass shift and SPR experiments confirmed direct binding to RIPK1, at a distinct site to allosteric type III RIPK1 inhibitors. The binding mode was further elucidated by a combination of X-ray crystallography and HDX-mass spectrometry, showing that the 7PQ series forms a key hydrogen bond with the hinge region of RIPK1 and thus may be classified as a type I kinase inhibitor. Notably, most previously published inhibitors for RIPK1 are Type II or III (Figure S4b).^18^ We subsequently established that **AZ’902** exhibited synergy with Nec-1s *in vitro* and demonstrated that a representative member of the 7PQ series is protective against inflammation *in vivo*, comparable to gold-standard necroptosis inhibitors. The 7PQ series possesses reasonable selectivity across a small panel of representative kinases, with the exception of Aurora B; interestingly, we note that the only previously reported type I RIPK1 inhibitors were developed starting from an Aurora kinase inhibitor.^18,33^

The characterization of the 7PQ series as RIPK1 inhibitors was originally unexpected, as anti-necroptotic compounds from the HTS were counter-screened and triaged based on the inhibition of RIPK1 or RIPK3 kinase activity.^20^ Hits were deprioritized for further investigation if they exhibited >50% inhibition at 1 μM in radiometric-binding (RIPK1) and FRET-based (RIPK3) assays. As such, the 7PQ compounds were not potent enough to be identified in this counter-screen. However, they exhibit comparative potency in *in vivo* inflammation models compared to the leading reference necroptosis inhibitor Nec-1. This highlights the challenges in deconvoluting results from HTS and provides a cautionary tale in using stringent target-based potency parameters for active compounds identified from phenotypic screens.

The synergy observed between the 7PQ series and necrostatins warrants future exploration. Synergy between Nec-1s and **AZ’902** was established within a specific range of concentrations, although no synergistic effect was observed by SPR. The co-crystal structure also did not show binding synergy between the compounds. Key structural motifs adjacent to the Nec-1s allosteric site, such as the DFG loop and C-α helix, are in the same inactive conformation observed when Nec-1s is bound to RIPK1 alone, suggesting that the mechanism of Nec-1s binding is unaffected by the 7PQ series. The typically disordered glycine-rich loop could be modeled in the co-crystal structure, but it is unclear whether this orientation is related to the synergy observed between the molecules. The evidence thus far suggests that the necrostatins and 7PQ series synergize in a functional manner rather than a simple biochemical synergy.

To conclude, we have validated and developed a novel chemotype for RIPK1 inhibition, which may offer a basis for future development of more potent RIPK1 inhibitors *in vitro* and *in vivo*, either alone or in combination with type III RIPK1 kinase inhibitors. Furthermore, the novel photoaffinity probe developed in this work may serve to establish the selectivity of the 7PQ series in cells, as well as to explore the mechanism of RIPK1 activity in necroptosis and disease.

## Experimental Section

### General

All reagents and solvents used were purchased from Acros Organics, Alfa Aesar, Fisher Scientific, Fluorochem, Sigma-Aldrich, Tokyo Chemical Industry, or VWR International Ltd. and used without further purification. For purification, high-performance liquid chromatography (HPLC)-grade solvents were used (Merck or Fisher Scientific). Reactions were monitored by thin layer chromatography (TLC) on Merck TLC silica gel 60 F254 aluminum sheets, and the compounds were visualized under UV (254 nm) or by liquid chromatography mass spectroscopy on an Agilent 1260 Infinity II MSD/XT Single Quad system. The analytical column was a Poroshell HPH-C18 3.0 × 50 mm with a flow rate of 0.8 mL/min and an injection volume of 2.50 μL. The solvent gradient started at 5% MeCN in water with 0.1% formic acid as an additive, ending at 95% MeCN after 7 min. Flash chromatography was carried out using Silica 60 (40–63 μm) silica gel (Merck), with the indicated solvent system according to standard techniques. For reactions that required heating, a hot plate was used with the appropriate DrySyn aluminum block. Nuclear magnetic resonance (NMR) spectra were recorded using a 300, 400, or 600 MHz Bruker spectrometer (^1^H NMR 300, 400, or 600 MHz, ^13^C NMR at 75, 101, or 151 MHz) and processed using MestReNova 12.0. Chemical shifts (δ) for ^1^H NMR spectra are reported in parts per million (ppm) relative to the internal standard (tetramethyl silane) and referenced to residual solvent (CDCl_3_ at 7.26 ppm, DMSO-*d*_6_ at 2.50 ppm, MeOD-*d*_4_ at 3.31 ppm). Chemical shifts (δ) for ^13^C NMR spectra are reported in parts per million (ppm) relative to the internal standard (tetramethylsilane) and referenced to the residual solvent (DMSO-*d*_6_ at 39.5 ppm, MeOD-*d*_4_ at 49.0 ppm). The following abbreviations are used for multiplicities: s—singlet, d—doublet, t—triplet, q—quartet, qi—quintet, m—multiplet, dd—doublet of doublets, br—broad. Coupling constants (*J*) are reported in Hz. All spectra were run at 25 °C unless otherwise stated. NMR spectra are included in the Supporting Information. The details of the intermediates’ syntheses are reported in the Supporting Information. The purity of all final compounds was >92%.

High-resolution mass spectra (HRMS) were recorded on a Micromass Autospec Premier through Imperial College London mass spectrometry services. Mass-to-charge ratios (*m*/*z*) were reported in Daltons, and HRMS was reported with less than 5 ppm error, or analytical HPLC analysis was carried out on an Alliance HPLC 2695 system (Waters, Ireland), equipped with an autosampler and photodiode array detector 2996 (Waters, Ireland). A Waters SunFire C18 5 μm (2.1 × 100 mm) reverse phase column was used with a constant flow rate of 0.3 mL min^–1^ and a gradient method of 30 min from 70:30 H_2_O (with 0.1% formic acid)/ACN to 5:95 H_2_O (with 0.1% formic acid)/ACN. Data acquisition and processing was carried out on MassLynx 4.1 software.

High-resolution electrospray ionization (ESI) positive mode mass spectrometry of **AZ’320** was carried out on a Bruker Impact II quadrupole-time-of-flight mass spectrometer (Bruker Daltonics) operating in high resolution mode. Samples were analyzed by flow injection analysis using an isocratic gradient 30:70 A/B, where A was a solution of 0.1% formic acid in water and B was a solution of 0.1% formic acid in acetonitrile, at a flow rate of 10 μL min^–1^ over 15 min. The scan mass spectra were acquired over a range of 100–1000 *m*/*z*, at a spectra rate of 1 Hz. Data Analysis 5.1 software was used to process spectra data.

AzTB was synthesized in-house as previously reported.^34^ Streptavidin magnetic beads (S1420S) were purchased from New England BioLabs. Neutravidin agarose resin (29201) and Pierce control agarose resin (26150) were purchased from Thermo Fisher Scientific.

Necroptosis inhibitors: Nec-1 (Fluorochem, #300433), Nec-1s (Cell signaling, #17802), GSK’872 (Abcam, #ab254395), NSA (Sigma-Aldrich, #480073).

IncuCyte reagents: recombinant human TNF was purchased from PeproTech (#300-01A). Sytox Green was purchased from Invitrogen (#S7020). Birinapant was purchased from Cambridge Bioscience (#2597-1) and Z-VAD-FMK from R&D systems (Biotechne; FMK001).

Antibodies: anti-RIPK1 (BD Biosciences, #610459), anti-pRIPK1 (Ser166) (Cell Signaling Technology, #65746S), anti-RIPK3 (Cell Signaling Technology, #13526S), anti-pRIPK3 (Ser227) (Cell Signaling Technology, #93654S), anti-MLKL (Novus, #NBP1-56729), anti-pMLKL (Ser358) (Cell Signaling Technology, #91689S), anti-GAPDH (Abcam, #Ab9485), antivinculin (Abcam, #Ab129002), antimouse HRP (Advansta, #R-05071-500), antirabbit HRP (Advansta, #R-05072-500).

### Synthesis of **AZ’902**


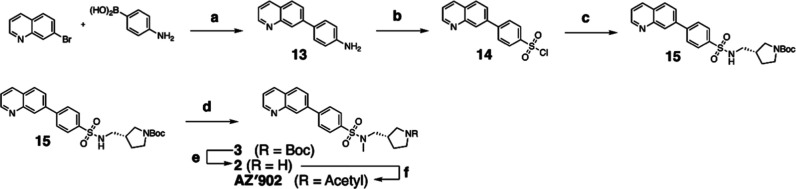


Reagents and conditions: (a) Pd­(PPh_3_)_4_, Na_2_CO_3_, H_2_O/MeCN, 130 °C, 18 h, 93%; (b) (i) conc. HCl, −15 °C, (ii) NaNO_2_, H_2_O (iii) SOCl_2_, CuCl, H_2_O, −15 °C, 1.5 h, 25%; (c) NEt_3_, CH_2_Cl_2_, rt, 18 h, 86%; (d) CH_3_I, K_2_CO_3_, DMF, rt, 4 h, 60%; (e) 4 M HCl in dioxane, N_2_, rt, 2 h, 97%; (f) acetic anhydride (Ac_2_O), pyridine, CH_2_Cl_2_, rt, 2 h, 68%.

#### (*R*)-*N*-((1-Acetylpyrrolidin-3-yl)­methyl)-*N*-methyl-4-(quinolin-7-yl)­benzenesulfonamide (**1**)


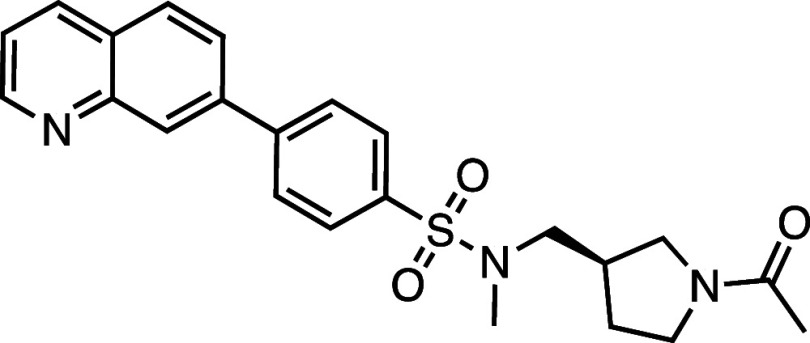


To a solution of (*R*)-*N*-methyl-*N*-(pyrrolidin-3-ylmethyl)-4-(quinolin-7-yl)­benzenesulfonamide (47 mg, 0.12 mmol) in anhydrous CH_2_Cl_2_ (5 mL) under a N_2_ atmosphere, pyridine (30 μL, 0.37 mmol) and then acetic anhydride (20 μL, 0.21 mmol) were added dropwise. The reaction mixture was stirred for 2 h at rt before quenching by pouring into saturated NaHCO_3_ (20 mL) and separated. The aqueous phase was extracted with CH_2_Cl_2_ (3 × 20 mL). The combined organics were then washed with 1 M HCl (20 mL), dried over anhydrous Na_2_SO_4_, filtered, and concentrated. Crude product was purified by flash column chromatography (5% MeOH/CH_2_Cl_2_) to give a pale yellow solid (36 mg, 68%). *R*_f_ = 0.28 (5% MeOH in CH_2_Cl_2_); ^1^H NMR (400 MHz, DMSO-*d*_6_, δ): 9.12 (1H, d, *J* = 4.9 Hz), 8.73 (1H, d, *J* = 8.3 Hz), 8.47 (1H, s), 8.28 (1H, d, *J* = 8.6 Hz), 8.14 (3H, t, *J* = 7.6 Hz), 7.95 (2H, d, *J* = 8.4 Hz), 7.78 (1H, dd, *J* = 8.3, 4.5 Hz), 3.58–3.49 (1H, m), 3.45–3.39 (1H, m), 3.30–3.15 (1H, m), 3.10–3.02 (1H, m), 3.02–2.99 (1H, m), 2.99–2.91 (1H, m), 2.75 (3H, d, *J* = 7.7 Hz), 2.63–2.51 (1H, m), 2.50–2.45, 2.06–1.95 (1H, m), 1.93 (3H, s), (d, *J* = 2.2 Hz), 1.77–1.54 (1H, m); ^13^C NMR (101 MHz, DMSO-*d*_6_, δ): 168.1, 149.6, 143.0, 140.8, 139.2, 136.5, 129.5 (2C), 128.3 (2C), 128.1 (2C), 127.9, 126.7, 124.3, 122.2, 52.1, 49.9, 48.5, 44.1, 36.9, 35.2, 28.9; HRMS (TOF MS ES^+^) found [M + H]^+^ 424.1700, C_23_H_26_N_3_O_3_S^+^ requires 424.1695.

#### (*S*)-*N*-Methyl-*N*-(pyrrolidin-3-ylmethyl)-4-(quinolin-7-yl)­benzenesulfonamide·HCl (**2**)


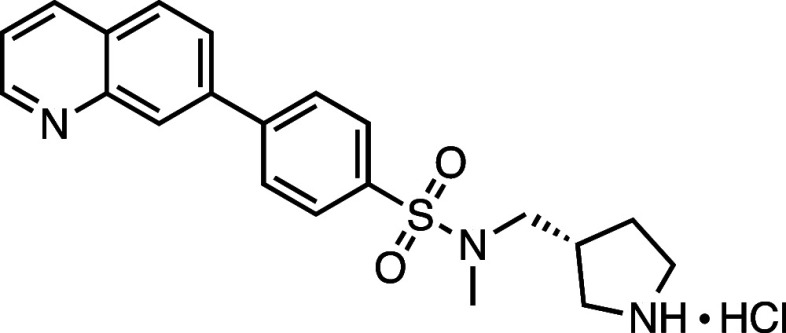


To a solution of *tert*-butyl (*S*)-3-(((*N*-methyl-4-(quinolin-7-yl)­phenyl)­sulfonamido)­methyl)­pyrrolidine-1-carboxylate (86 mg, 0.18 mmol) in CH_2_Cl_2_ (0.5 mL) under a nitrogen atmosphere was added 4 M HCl in 1,4-dioxane (0.8 mL) dropwise, and the mixture was stirred at rt for 2 h. The reaction mixture was concentrated under reduced pressure and triturated in diethyl ether (50 mL) to yield the product as a brown solid precipitate (66 mg, 0.17 mmol, 97%), which was used without purification. *R*_f_ = 0.42 (5% MeOH/CH_2_Cl_2_); ^1^H NMR (400 MHz; MeOD, δ): 9.29 (2H, t, *J* = 7.5 Hz), 8.52 (2H, d, *J* = 10.4 Hz), 8.37 (1H, d, *J* = 8.5 Hz), 8.15 (3H, d, *J* = 8.3 Hz), 8.05 (2H, d, *J* = 8.2 Hz), 3.52–3.39 (2H, m), 3.39–3.30 (1H, m) 3.24–3.17 (1H, m), 3.16–3.07 (2H, m), 2.85 (3H, s), 2.83–2.74 (1H, m), 2.27–2.14 (1H, m), 1.92–1.82 (1H, m); ^13^C NMR (101 MHz; MeOD, δ): 148.4, 147.0, 146.8, 143.9, 139.7, 139.2, 131.5, 130.7, 130.2, 130.0 (2C), 129.7 (2C), 123.4, 119.7, 53.3, 46.3, 37.8, 36.0, 28.8 (2C); HRMS (TOF MS ES^+^) found [M + H]^+^ 382.1586, [C_21_H_24_N_3_O_2_S]^+^ requires 382.1589.

#### *tert*-Butyl­(*S*)-3-(((*N*-methyl-4-(quinolin-7-yl)­phenyl)­sulfonamido)­methyl)­pyrrolidine-1-carboxylate (**3**)


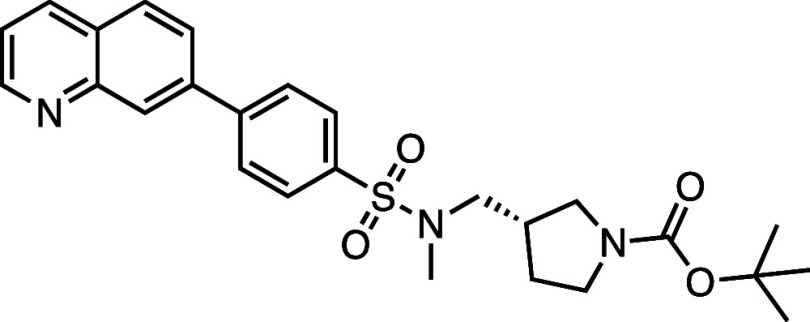


To the solution of *tert*-butyl (*S*)-3-(aminomethyl)­pyrrolidine-1-carboxylate (142 mg, 0.71 mmol) in CH_2_Cl_2_ (5 mL) at 0 °C, triethylamine (0.27 mL, 1.9 mmol) was added. 4-(Quinolin-7-yl)­benzenesulfonyl chloride **14** (175 mg, 0.58 mmol) in CH_2_Cl_2_ (15 mL) was added dropwise to the mixture and stirred at 60 °C overnight. The reaction mixture was diluted with CH_2_Cl_2_ (50 mL) and H_2_O (50 mL) and separated. The aqueous phase was further extracted with CH_2_Cl_2_ (50 mL). Combined organics were washed with water (50 mL) and brine (50 mL), then dried over anhydrous Na_2_SO_4_, filtered, and concentrated under reduced pressure. The crude product was purified by flash column chromatography (80% EtOAc/*n*-hexane) to give a pale yellow solid (188 mg, 0.40 mmol, 86%). *R*_f_ = 0.24 (80% EtOAc/*n*-hexane); ^1^H NMR (400 MHz; CDCl_3_, δ): 8.98 (1H, d, *J* = 4.3 Hz), 8.34 (1H, s), 8.22 (1H, d, *J* = 8.3 Hz), 7.98 (2H, d, *J* = 8.1 Hz), 7.94 (1H, d, *J* = 8.4 Hz), 7.89 (2H, d, *J* = 8.2 Hz), 7.81 (1H, d, *J* = 8.4 Hz), 7.46 (1H, dd, *J* = 8.4, 4.3 Hz), 5.02–4.94 (1H, m), 3.49 (1H, dd, *J* = 11.0, 7.4 Hz), 3.41 (1H, t, *J* = 10.2 Hz), 3.29 (1H, t, *J* = 8.5 Hz), 3.03 (3H, t, *J* = 6.8 Hz), 2.39 (1H, m), 2.02–1.91 (1H, m), 1.60 (1H, d, *J* = 7.6 Hz), 1.43 (9H, s); ^13^C NMR (101 MHz; CDCl_3_, δ): 154.6, 151.4, 148.5, 144.9, 136.0, 132.3, 128.9, 128.6, 128.3 (2C), 128.1, 128.0, 127.9 (2C), 126.0, 121.8, 79.5, 51.6, 49.4, 45.9, 39.3, 38.4, 28.6; HRMS (TOF MS ES^+^) found [M + H]^+^ 468.1947, [C_25_H_30_N_3_O_4_S]^+^ requires 468.1957.

#### (*S*)-*N*-((1-Acetylpyrrolidin-3-yl)­methyl)-*N*-methyl-4-(quinolin-7-yl)­benzenesulfonamide (**AZ’902**)


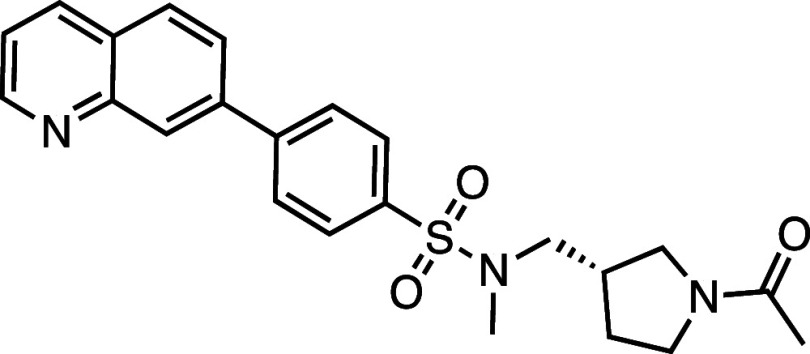


To a solution of (*S*)-*N*-methyl-*N*-(pyrrolidin-3-ylmethyl)-4-(quinolin-7-yl)­benzenesulfonamide (47 mg, 0.12 mmol) in anhydrous CH_2_Cl_2_ (5 mL) under a nitrogen atmosphere, pyridine (30 μL, 0.37 mmol), then acetic anhydride (20 μL, 0.21 mmol) were added dropwise. The reaction mixture was stirred for 2 h at rt before quenching by pouring into saturated NaHCO_3_ (20 mL) and separated. The aqueous phase was extracted with CH_2_Cl_2_ (3 × 20 mL). The combined organics were then washed with 1 M HCl (20 mL), dried over anhydrous Na_2_SO_4_, filtered, and concentrated under reduced pressure. Crude product was purified by flash column chromatography (5% MeOH/CH_2_Cl_2_) to give the product as a pale yellow solid (36 mg, 0.084 mmol, 68%). *R*_f_ = 0.28 (5% MeOH/in CH_2_Cl_2_); ^1^H NMR (400 MHz; DMSO-*d*_6,_) 8.98 (1H, dd, *J* = 4.3, 1.7 Hz), 8.44 (1H, d, *J* = 8.5 Hz), 8.38 (1H, s), 8.14 (3H, dd, *J* = 8.0, 3.6 Hz), 8.03 (1H, dd, *J* = 8.5, 1.9 Hz), 7.92 (2H, d, *J* = 8.1 Hz), 7.59 (1H, dd, *J* = 8.3, 4.2 Hz), 3.58–3.47 (1H, m), 3.47–3.37 (1H, m), 3.30–3.15 (1H, m), 3.10–3.03 (1H, m), 3.04–2.97 (1H, m), 2.94 (1H, dd, *J* = 13.3, 7.0 Hz), 2.75 (3H, d, *J* = 7.7 Hz), 2.58 (1H, d, *J* = 7.1 Hz), 2.04–1.97 (1H, m), 1.93 (3H, d, *J* = 2.3 Hz), 1.79–1.55 (1H, m); ^13^C NMR (101 MHz; DMSO-*d*_6_) 168.1, 149.6, 143.0, 140.8, 139.2, 136.5, 129.5 (2C), 128.3 (2C), 128.1 (2C), 127.9, 126.7, 124.3, 122.2, 52.1, 49.9, 48.5, 44.1, 36.9, 35.2, 28.9; HRMS (TOF MS ES^+^) found [M + H]^+^ 424.1700, [C_23_H_26_N_3_O_3_S]^+^ requires 424.1695.

### Synthesis of 7PQ Analogues


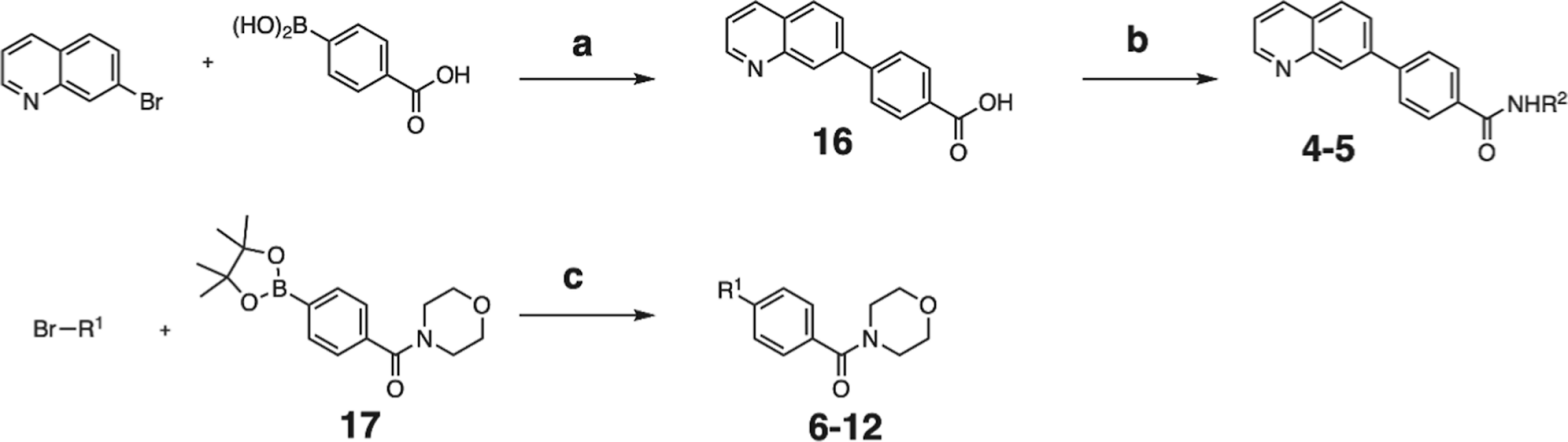


Reagents and conditions: (a) Pd­(PPh_3_)_4_, Na_2_CO_3_, H_2_O/MeCN, 90 °C, 18 h, 78%; (b) R^2^-NH_2_, DCC, DMAP, CH_2_Cl_2_, rt, 18 h; (c) R^1^-Br, Pd­(PPh_3_)_4_, Na_2_CO_3_, H_2_O/MeCN, 130 °C, 18 h.

#### General Procedure b

To a solution of 4-(quinolin-7-yl)­benzoic acid **16** (0.40 mmol), DMAP (0.80 mmol) and DCC (0.60 mmol) in CH_2_Cl_2_ (10 mL), amine (0.48 mmol) was added and stirred at rt overnight. The mixture was filtered, and the filtrate was washed with water (2 × 20 mL) and brine (2 × 20 mL), then dried over anhydrous Na_2_SO_4_, filtered, and concentrated. Crude product was purified by flash column chromatography.

#### General Procedure c

To a solution of aryl bromide (0.32 mmol) and 4-(morpholine-4-carbonyl)­phenylboronic acid pinacol ester **17** (0.32 mmol) in MeCN (5 mL) was added a solution of Na_2_CO_3_ (0.16 mmol) in H_2_O (5 mL). Pd­(PPh_3_)_4_ (0.01 mmol) was added, and the reaction mixture was stirred at 130 °C overnight. The reaction mixture was cooled to rt, filtered through Celite, and the Celite was washed with EtOAc (2 × 20 mL). The filtrate was concentrated under reduced pressure, and the remaining aqueous portion was diluted with EtOAc (20 mL) and H_2_O (20 mL) and separated. The aqueous phase was extracted with EtOAc (2 × 20 mL). Combined organics were washed with water (20 mL) and brine (20 mL), then dried over anhydrous Na_2_SO_4_, filtered, and concentrated under reduced pressure. Crude product was purified by flash column chromatography.

#### *tert*-Butyl (*R*)-3-((4-(Quinolin-7-yl)­benzamido)­methyl)­pyrrolidine-1-carboxylate (**4**)


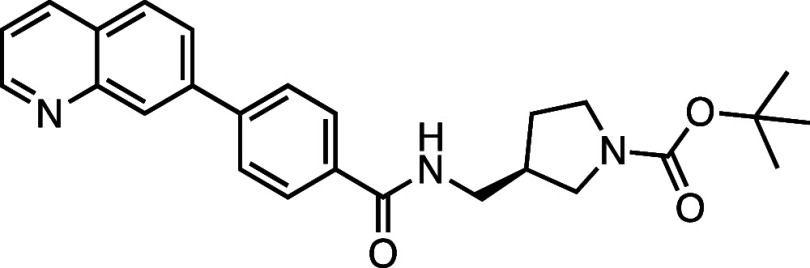


Prepared according to General Procedure b, the crude product was purified on silica gel (80% EtOAc/*n*-hexane); the product was a white solid (78 mg, 0.18 mmol, 45%); *R*_f_ = 0.17 (90% EtOAc/*n*-hexane). ^1^H NMR (400 MHz; DMSO-*d*_6_, δ): 8.96 (1H, dd, *J* = 4.3, 1.7 Hz), 8.70 (1H, br), 8.42 (1H, d, *J* = 8.5 Hz), 8.35 (1H, s), 8.11 (1H, d, *J* = 8.5 Hz), 8.03 (1H, d, *J* = 2.1 Hz), 8.00 (4H, d, *J* = 3.9 Hz), 7.56 (1H, dd, *J* = 8.3, 4.2 Hz), 3.44–3.35 (2H, m), 3.33–3.15 (2H, m), 3.02 (1H, m), 2.45 (1H, d, *J* = 6.2 Hz), 1.92 (1H, d, *J* = 6.3 Hz), 1.74–1.56 (2H, m), 1.39 (9H, s); ^13^C NMR (101 MHz, DMSO-*d*_6_, δ): 166.0, 153.6, 151.2, 148.0, 141.8, 140.1, 135.8, 133.8, 128.9, 128.1 (2C), 127.4, 127.1 (2C), 126.5, 125.7, 121.7, 78.2, 49.3, 49.1, 45.1, 44.9, 41.5, 28.2 (3C); HRMS (TOF MS ES^+^) found [M + H]^+^ 432.2280, [C_26_H_30_N_3_O_3_]^+^ requires 432.2287.

#### *tert*-Butyl (*R*)-3-(4-(Quinolin-7-yl)­benzamido)­pyrrolidine-1-carboxylate (**5**)


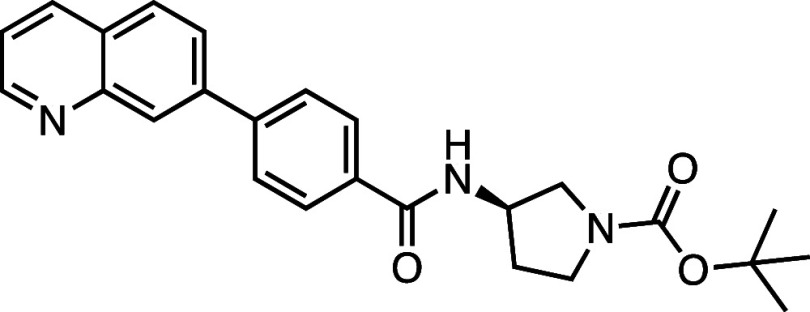


Prepared according to General Procedure b, the crude product was purified on silica gel (80% EtOAc/*n*-hexane); the product was a pale yellow solid (52 mg, 0.12 mmol, 31%). *R*_f_ = 0.27 (90% EtOAc/*n*-hexane); ^1^H NMR (400 MHz, DMSO-*d*_6_, δ): 8.95 (1H, s), 8.66 (1H, d, *J* = 6.5 Hz), 8.41 (1H, d, *J* = 7.6 Hz), 8.35 (1H, s), 8.10 (1H, d, *J* = 8.1 Hz), 8.06–7.94 (5H, m), 7.59–7.52 (1H, m), 4.53–4.35 (1H, m), 3.65–3.47 (2H, m), 3.31–3.18 (2H, m), 2.22–2.05 (1H, m), 2.01–1.85 (1H, m), 1.41 (3H, s); ^13^C NMR (101 MHz, DMSO-*d*_6_, δ): 166.1, 153.5, 151.2, 148.0, 141.9, 140.1, 135.8, 133.6, 128.9, 128.3 (2C), 127.4, 127.0 (2C), 126.5, 125.7, 121.7, 78.3, 50.8, 49.6, 48.9, 43.9, 28.2 (3C); HRMS (TOF MS ES^+^) found [M + H]^+^ 418.2121, C_25_H_28_N_3_O_3_^+^ requires 418.2131.

#### Morpholino­(4-(quinolin-7-yl)­phenyl)­methanone (**6**)


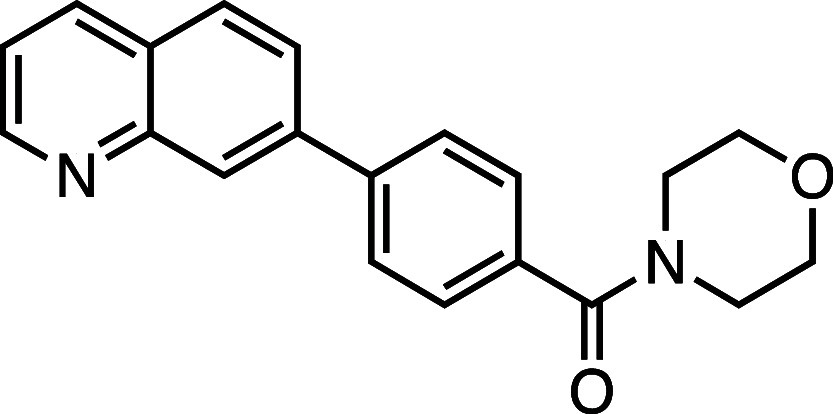


Prepared according to General Procedure c using 7-bromoquinoline, the crude product was purified on silica gel (5% MeOH/CH_2_Cl_2_); the product was a white solid (61 mg, 0.18 mmol, 90%). *R*_f_ = 0.61 (70% EtOAc/*n*-hexane); ^1^H NMR (400 MHz; CDCl_3_, δ): 8.97–8.96 (1H, d, *J* = 8.0 Hz), 8.34 (1H, s), 8.21–8.19 (1H, d, *J* = 8.0 Hz), 7.93–7.79 (3H, m), 7.57–7.54 (2H, m), 7.45–7.42 (1H, m), 3.99–3.61 (8H, m); ^13^C NMR (101 MHz, CDCl_3_, δ): 151.1, 147.6, 146.9, 142.8, 134.5, 128.5, 128.0, 127.7, 127.5, 126.0, 124.0, 123.7, 121.9, 67.6, 61.7; HRMS (TOF MS ES^+^) found [M + H]^+^ 319.1448, [C_20_H_19_N_2_O_2_]^+^ requires 319.1447.

#### (4-(Isoquinolin-7-yl)­phenyl)­(morpholino)­methanone (**7**)


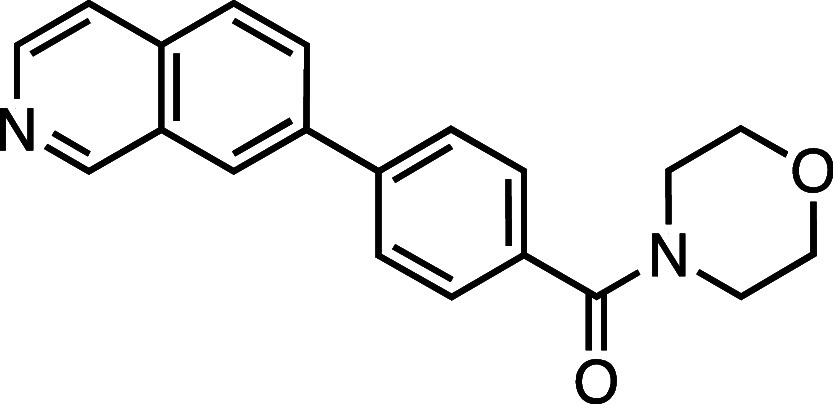


Prepared according to General Procedure c, using 7-bromoisoquinoline, the crude product was purified on silica gel (70% EtOAc/*n*-hexane); the product was an off-white solid (73 mg, 0.23 mmol, 72%). *R*_f_ = 0.11 (80% EtOAc/*n*-hexane); ^1^H NMR (400 MHz; DMSO-*d*_6_, δ): 9.42 (1H, s), 8.54 (1H, s), 8.49 (1H, s), 8.17 (1H, d, *J* = 8.6 Hz), 8.09 (1H, d, *J* = 8.6 Hz), 7.93 (2H, d, *J* = 8.0 Hz), 7.89 (1H, d, *J* = 5.6 Hz), 7.58 (2H, d, *J* = 7.9 Hz), 3.63 (8H, br); ^13^C NMR (101 MHz, DMSO-*d*_6_, δ): 168.8, 152.8, 143.0, 140.4, 138.1, 135.0, 134.6, 129.6, 128.0 (2C), 127.9, 127.4, 127.1 (2C), 125.4, 120.2, 66.1 (4C); HRMS (TOF MS ES^+^) found [M + H]^+^ 319.1443, [C_20_H_19_N_2_O_2_]^+^ requires 319.1447.

#### (4-(Isoquinolin-6-yl)­phenyl)­(morpholino)­methanone (**8**)


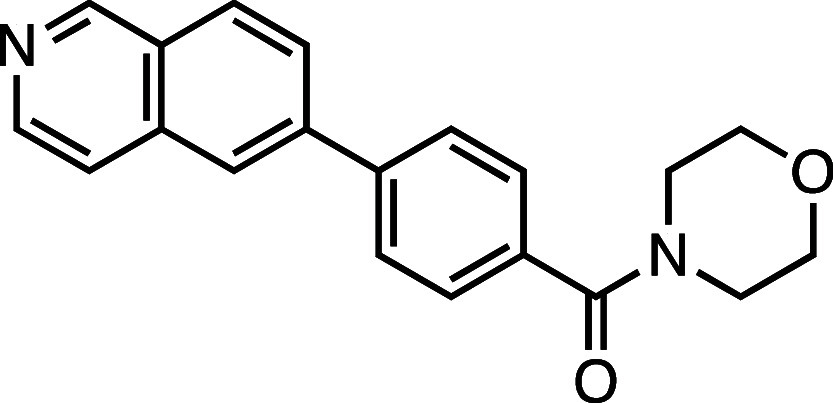


Prepared according to General Procedure c, using 6-bromoisoquinoline, the crude product was purified on silica gel (70% EtOAc/*n*-hexane); the product was an off white solid (73 mg, 0.23 mmol, 73%); *R*_f_ = 0.11 (80% EtOAc/*n*-hexane); ^1^H NMR (400 MHz; DMSO-*d*_6_, δ): 9.36 (1H, s), 8.54 (1H, s), 8.32 (1H, s), 8.23 (1H, d, *J* = 8.6 Hz), 8.05 (1H, d, *J* = 8.6 Hz), 7.92 (3H, t, *J* = 8.6 Hz), 7.58 (2H, d, *J* = 7.8 Hz), 3.63 (8H, br); ^13^C NMR (101 MHz, DMSO-*d*_6_, δ): 168.7, 152.1, 143.3, 141.1, 140.3, 135.6, 135.3, 128.5, 128.0 (2C), 127.4 (2C), 126.9, 126.6, 124.2, 120.8, 66.1 (4C); HRMS (TOF MS ES^+^) found [M + H]^+^ 319.1443, [C_20_H_19_N_2_O_2_]^+^ requires 319.1447.

#### Morpholino­(4-(quinolin-6-yl)­phenyl)­methanone (**9**)


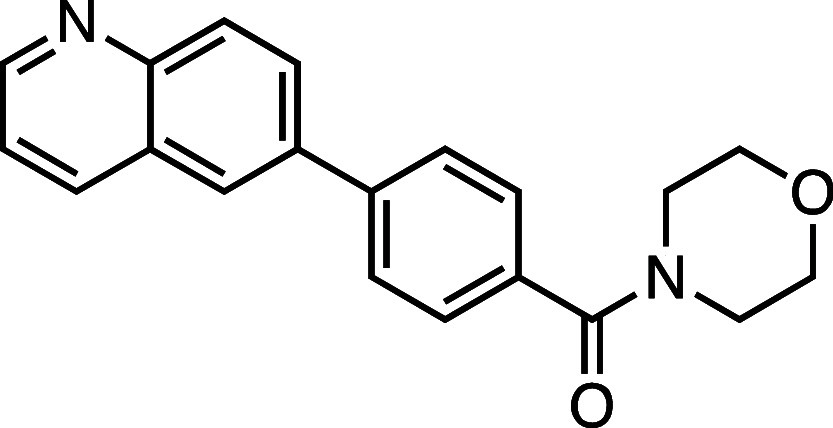


Prepared according to General Procedure c, using 6-bromoquinoline, the crude product was purified on silica gel (70% EtOAc/*n*-hexane); the product was an off-white solid (73 mg, 0.23 mmol, 73%). *R*_f_ = 0.16 (80% EtOAc/*n*-hexane); ^1^H NMR (400 MHz, DMSO-*d*_6_, δ): 8.92 (1H, d, *J* = 2.6 Hz), 8.45 (1H, d, *J* = 8.0 Hz), 8.35 (1H, s), 8.12 (2H, s), 7.92 (2H, d, *J* = 8.0 Hz), 7.61–7.55 (3H, m), 3.63 (8H, br); ^13^C NMR (101 MHz; DMSO-*d*_6_, δ): 168.8, 150.9, 147.3, 140.4, 137.1, 136.5, 134.9, 129.6, 128.5, 128.2, 127.9 (2C), 127.1 (2C), 125.9, 122.0, 66.1 (4C); HRMS (TOF MS ES^+^) found [M + H]^+^ 319.1433, [C_20_H_19_N_2_O_2_]^+^ requires 319.1447.

#### Morpholino­(4-(quinolin-3-yl)­phenyl)­methanone (**10**)


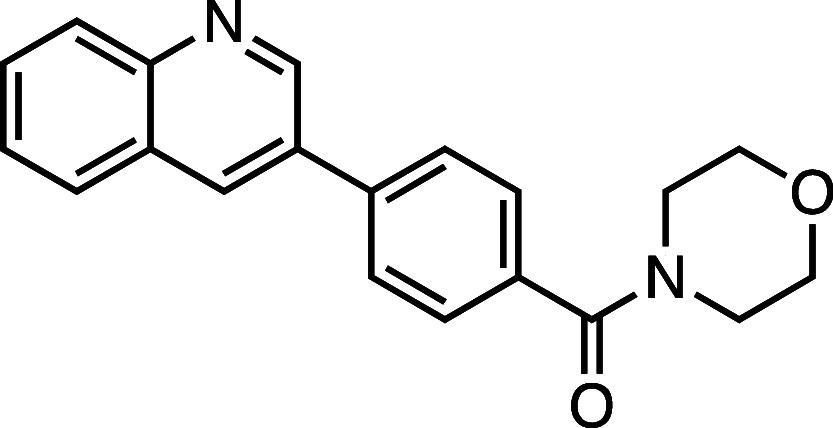


Prepared according to General Procedure c, using 3-bromoquinoline, the crude product was purified on silica gel (70% EtOAc/*n*-hexane); the product was an off-white solid (82 mg, 0.26 mmol, 82%). *R*_f_ = 0.22 (80% EtOAc/*n*-hexane); ^1^H NMR (400 MHz, DMSO-*d*_6_, δ): 9.29 (1H, s), 8.72 (1H, s), 8.07 (2H, d, *J* = 8.2 Hz), 7.98 (2H, d, *J* = 7.9 Hz), 7.80 (1H, t, *J* = 7.7 Hz), 7.67 (1H, t, *J* = 7.6 Hz), 7.60 (2H, d, *J* = 7.8 Hz), 3.64 (8H, br); ^13^C NMR (101 MHz, DMSO-*d*_6_, δ): 168.7, 149.3, 147.0, 138.2, 135.2, 133.3, 132.0, 129.8, 128.7, 128.5, 128.0 (2C), 127.6, 127.2 (2C), 127.1, 66.1 (4C); HRMS (TOF MS ES^+^) found [M + H]^+^ 319.1439, [C_20_H_19_N_2_O_2_]^+^ requires 319.1447.

#### Morpholino­(4-(quinolin-2-yl)­phenyl)­methanone (**11**)


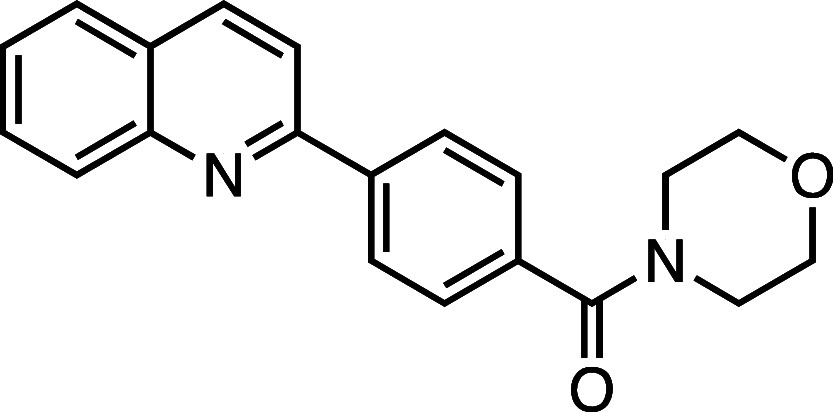


Prepared according to General Procedure c, using 2-bromoquinoline, the crude product was purified on silica gel (70% EtOAc/*n*-hexane); the product was a white solid (86 mg, 0.27 mmol, 85%). *R*_f_ = 0.28 (80% EtOAc/*n*-hexane); ^1^H NMR (400 MHz; DMSO-*d*_6_, δ): 8.49 (1H, d, *J* = 8.6 Hz), 8.35 (2H, d, *J* = 7.8 Hz), 8.20 (1H, d, *J* = 8.7 Hz), 8.09 (1H, d, *J* = 8.5 Hz), 8.02 (1H, d, *J* = 8.1 Hz), 7.80 (1H, t, *J* = 7.7 Hz), 7.65–7.56 (3H, m), 3.85–3.37 (8H, m); ^13^C NMR (101 MHz, DMSO-*d*_6_, δ): 168.8, 155.3, 147.5, 139.6, 137.4, 136.5, 130.1, 129.2, 128.8, 127.9, 127.7, 127.3, 127.1, 126.7, 118.8, 114.9, 66.1 (4C); HRMS (TOF MS ES^+^) found [M + H]^+^ 319.1440, [C_20_H_19_N_2_O_2_]^+^ requires 319.1447.

#### Morpholino­(4-(quinoxalin-6-yl)­phenyl)­methanone (**12**)


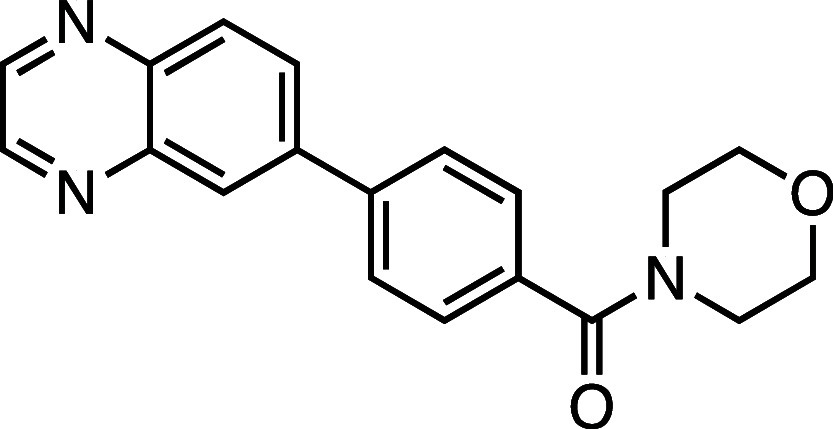


Prepared according to General Procedure c, using 6-bromoquinoxaline, the crude product was purified on silica gel (80% EtOAc/*n*-hexane); the product was a white solid (65 mg, 0.20 mmol, 70%). *R*_f_ = 0.19 (100% EtOAc); ^1^H NMR (400 MHz, DMSO-*d*_6_, δ): 8.99 (2H, d, *J* = 12.7 Hz), 8.40 (1H, s), 8.25 (1H, dd, *J* = 8.7, 2.1 Hz), 8.20 (1H, d, *J* = 8.7 Hz), 7.98 (2H, d, *J* = 8.0 Hz), 7.59 (2H, d, *J* = 8.0 Hz), 3.64 (8H, br); ^13^C NMR (101 MHz, DMSO-*d*_6_, δ): 168.7, 146.4, 145.9, 142.5, 141.8, 140.9, 139.7, 135.4, 129.9, 129.4, 128.0 (2C), 127.5 (2C), 126.5, 66.1 (4C); HRMS (TOF MS ES^+^) found [M + H]^+^ 320.1405, [C_19_H_18_N_3_O_2_]^+^ requires 320.1399.

### Synthesis of the Affinity-Based Probe **7PQYnD**


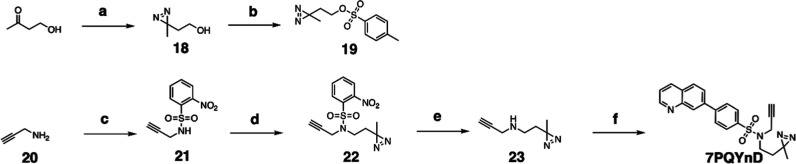


Reagents and conditions: (a) (i) NH_3_, MeOH, 5 h, −78 °C, (ii) hydroxylamine-*O*-sulfonic acid (HOSA), 16 h, −78 °C to rt, (iii) I_2_, DIPEA, MeOH, 1 h, 0 °C, 36%; (b) *p*-toluenesulfonyl chloride (*p*-TsCl), pyridine, 3 h, 0 °C to rt, 65%; (c) 2-nitrobenzenesulfonyl chloride, NEt_3_, CH_2_Cl_2_, 3 h, 0 °C to rt, N_2_, 88%; (d) **21**, K_2_CO_3_, DMF, 5 h, 80 °C, 78%; (e) LiOH·H_2_O, 3-mercaptopropionic acid (HS­(CH_2_)_2_COOH), DMF, 5 h, rt, 75%; (f) **14**, NEt_3_, CH_2_Cl_2_, 0 °C to rt, 2.5 h, 48%.

#### *N*-(2-(3-Methyl-3*H*-diazirin-3-yl)­ethyl)-*N*-(prop-2-yn-1-yl)-4-(quinolin-7-yl)­benzenesulfonamide (**7PQYnD**)


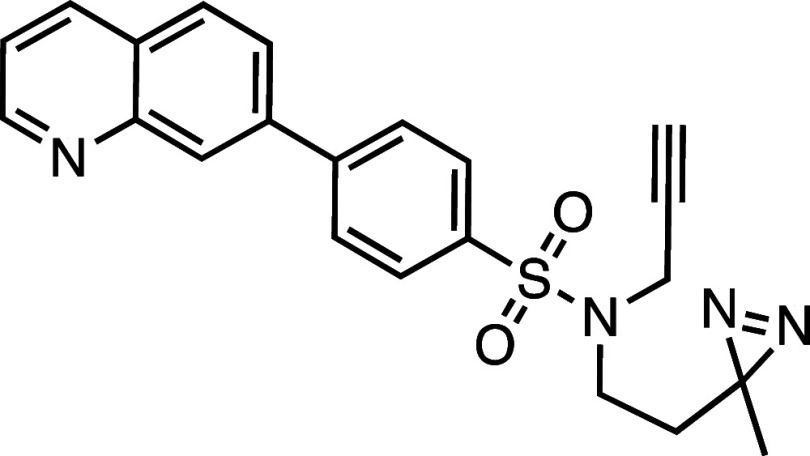


Triethylamine (0.18 mL, 1.3 mmol, 2.1 equiv) was added to a stirred solution of *N*-(2-(3-methyl-3*H*-diazirin-3-yl)­ethyl)­prop-2-yn-1-amine **19** (100 mg, 0.73 mmol, 1.2 equiv) in CH_2_Cl_2_ (2 mL) and the solution cooled to 0 °C. Crude 4-(quinolin-7-yl)­benzenesulfonyl chloride **14** (185 mg, 0.61 mmol, 1 equiv) was added and the reaction stirred at rt for 2.5 h. The reaction mixture was diluted with CH_2_Cl_2_ (20 mL) and H_2_O (20 mL) and separated. The aqueous phase was further extracted with CH_2_Cl_2_ (20 mL) and the combined organics were washed successively with H_2_O (20 mL) and brine (20 mL), then dried over anhydrous MgSO_4_, filtered, and concentrated in vacuo. The crude product was purified by automated flash column chromatography (10–100% EtOAc in *n*-hexane) to afford the product as an off-white solid (118 mg, 0.29 mmol, 48%). ^1^H NMR (400 MHz, CDCl_3_) 8.98 (1H, d, *J* = 3.4 Hz), 8.40–8.34 (1H, m), 8.27–8.19 (1H, m), 8.00–7.96 (2H, m), 7.95 (1H d, *J* = 8.1 Hz), 7.92–7.87 (2H, m), 7.83 (1H, dd, *J* = 8.5, 1.8 Hz), 7.47 (1H, dd, *J* = 8.3, 4.2 Hz), 4.18 (2H, d, *J* = 2.5 Hz), 3.32–3.22 (2H, m), 2.05 (1H t, *J* = 2.5 Hz), 1.69–1.60 (2H, m), 1.09 (3H, s); ^13^C NMR (101 MHz, CDCl_3_) 151.2, 148.3, 145.0, 140.4, 137.7, 138.4, 136.2, 128.9, 128.6 (2C), 128.1 (2C), 127.9, 126.0, 121.8, 76.2, 74.5, 42.0, 36.8, 33.5, 24.2, 19.6; HRMS (TOF MS ESI^+^) found [M + H]^+^ 405.1381, [C_22_H_21_N_4_O_2_S]^+^ requires 405.1385.

### Synthesis of **AZ’320**


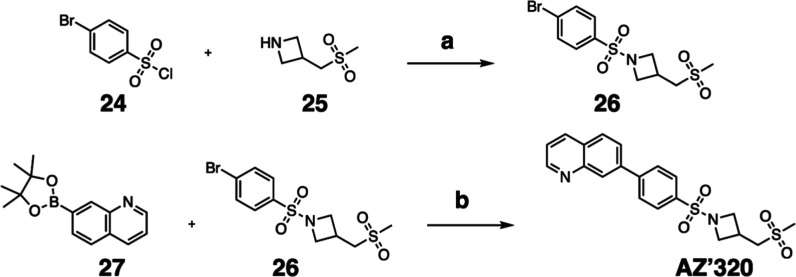


Synthetical procedure to prepare compound **AZ’320**. Reagents and conditions: (a) TEA, N_2_, rt, 16 h, 93%; (b) PdCl_2_(PPh_3_)_2_, XPhos, K_2_CO_3_, TBAB, N_2_, 95 °C, overnight, 71%.

#### 7-(4-((3-((Methylsulfonyl)­methyl)­azetidin-1-yl)­sulfonyl)­phenyl)­quinoline (**AZ’320**)


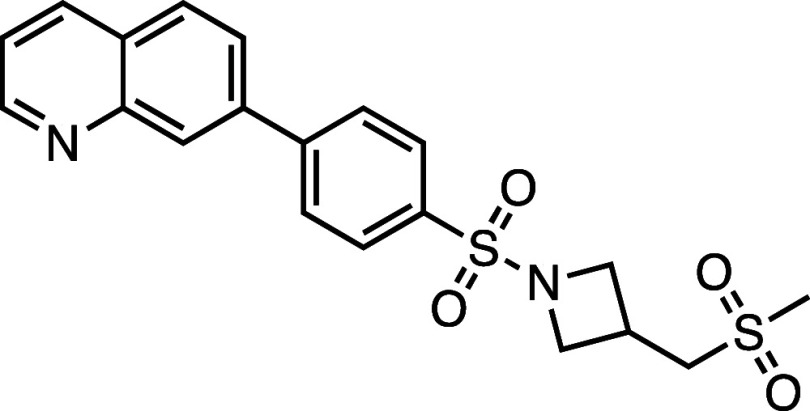


A solution of 1-((4-bromophenyl)­sulfonyl)-3-((methylsulfonyl)­methyl)­azetidine **26** (52 mg, 0.20 mmol, 1.5 equiv), 7-quinolinyl boronic acid pinacol ester **27** (50 mg, 0.14 mmol, 1 equiv), PdCl_2_(PPh_3_)_2_ (9.5 mg, 14 μmol, 0.1 equiv), and XPhos (6.5 mg, 14 μmol, 0.1 equiv) in dioxane (1.8 mL) was stirred for 0.5 h at 95 °C. A tetrabutylammonium bromide solution (1.36 mL, 0.14 mmol, 1 equiv) was added, and the mixture was stirred for 5 min, followed by the addition of K_2_CO_3_ solution (0.170 mL, 0.68 mmol, 5 equiv). The reaction mixture was stirred overnight at 95 °C. After completion, the mixture was diluted in H_2_O (20 mL) and extracted with EtOAc (4 × 20 mL). The catalyst was removed by vacuum filtration through a pad of Celite. The filtrate was dried with anhydrous Na_2_SO_4_, and the solvent was removed under reduced pressure. The crude product was purified by flash chromatography on silica gel to obtain the product as a white solid (40 mg, 97 μmol, 71%). ^1^H NMR (300 MHz, DMSO-*d*_6_, δ): 8.97 (1H, dd, *J* = 4.2, 1.8 Hz), 8.42 (1H, dd, *J* = 7.5, 1.7 Hz), 8.41 (1H, s), 8.19 (2H, d, *J* = 8.3 Hz), 8.13 (1H, d, *J* = 8.6 Hz), 8.03 (1H, dd, *J* = 8.5, 1.8 Hz), 7.95 (1H, d, *J* = 8.4 Hz), 7.57 (1H, dd, *J* = 8.3, 4.2 Hz), 4.00–3.87 (2H, m), 3.63 (2H, dd, *J* = 8.5, 6.4 Hz), 3.27 (2H, d, *J* = 7.5 Hz), 3.02–2.93 (1H, m), 2.91 (3H, s). ^13^C NMR (75 MHz, DMSO-*d*_6_, δ): 151.4, 147.9, 144.1, 139.2, 135.8, 133.1, 129.1, 128.9, 128.2, 127.7, 127.1, 125.6, 122.0, 56.0, 55.2, 40.4, 22.8. HRMS (TOF MS ESI^+^) found [M + H]^+^ 417.0937, and [C_20_H_20_N_2_O_4_S]^+^ requires 417.0939. HPLC: retention time 2.37 min, 100%.

### Cell Culture

All cell culturing was carried out in a sterile tissue culture cabinet sprayed with 70% (v/v) EtOH before and after use. All cell lines were cultured at 37 °C in a 5% CO_2_ incubator. FADD-deficient Jurkat T (I2.1) cells (ATCC, CRL-2572) were cultured in Roswell Park Memorial Institute 1640 medium (RPMI-1640, Gibco) supplemented with 10% (v/v) fetal bovine serum (FBS, Gibco). HT-29 cells (kind gift from Gad Frankel) were cultured in RPMI-1640 medium supplemented with 10% (v/v) FBS, 2 mM GlutaMAX (Gibco), 1 mM sodium pyruvate (Gibco), 10 mM HEPES (Gibco), and 2.5 mg mL^–1^ glucose. Passage numbers were limited to 20–25.

### IncuCyte Necroptosis Assay

I2.1 cells were seeded in a sterile 96-well plate (Greiner) at 20,000 cells per well and incubated at 37 °C overnight. The cells were treated with a final concentration of 600 pM TNF, 1 μM Sytox Green, and compounds in a dose-dependent manner. Wells containing TNF and Sytox Green served as positive control. Wells containing DMSO and Sytox Green served as a negative control. The plate was placed into an IncuCyte S3 Live Cell Analysis System (Sartorius) and readings for phase and green fluorescence were taken every hour for 24 h, with 4 images per well. The images were analyzed with IncuCyte 2021C software and the Green Integrated Intensity Per Image/Phase Area Per Image (GCU × μm^2^/image/μm^2^/image or GP) was exported. The background at *t* = 0 h was subtracted, and the linear slope (*t* = 3–7 h) was calculated by plotting GP against time. The percent inhibition of necroptosis was determined by normalizing to the positive and negative controls and graphing against the [compound]. EC_50_ values were determined using Prism 10 for each replicate, which were then used to calculate the geometric mean EC_50_ and associated 95% CIs.

HT-29 cells were seeded in a sterile 96-well plate (Greiner) at 20,000 cells per well and incubated at 37 °C overnight. The cells were treated with a final concentration of 1.2 nM TNF, 100 nM Birinapant, 20 μM Z-VAD-FMK, 250 nM Sytox Green, and compounds in a dose-dependent manner. Wells containing TSZ served as a positive control. Wells containing DMSO and Sytox Green served as a negative control. The plate was placed into an IncuCyte S3 Live Cell Analysis System (Sartorius) and readings for phase and green fluorescence were taken every hour for 48 h, with 4 images per well. The images were analyzed with IncuCyte 2021C software, and the green integrated intensity per image/phase area per image (GCU × μm^2^/image/μm^2^/image or GP) was exported. The background at *t* = 0 h was subtracted and the linear slope (*t* = 35–45 h) calculated by plotting GP against time. The percent inhibition of necroptosis was determined by normalizing to the positive and negative control and graphing against the [compound]. EC_50_ values were generated from nonlinear regression fits performed on GraphPad Prism 10 for each replicate, which were then used to calculate the geometric mean EC_50_ and associated 95% CIs.

### Cell Treatments and Western Blots

I2.1 cells were seeded in sterile six-well plates at a density of 2 × 10^6^ cells per well and incubated at 37 °C overnight. All compound treatments were performed with a preprepared 1000× stock of the desired concentration in DMSO and added directly to the relevant plate, with a final concentration of 0.1% DMSO on the cells. TNF (final concentration 575 pM) was added to induce necroptosis, the cells incubated for 4 h and harvested.

HT-29 cells were seeded in sterile six-well plates at a density of 1.5 × 10^6^ cells per well and incubated at 37 °C overnight. All compound treatments were performed with a preprepared 1000× stock of the desired concentration and added directly to the relevant plate with a final concentration of 0.1% DMSO on the cells. A combination of TNF (final concentration of 1.2 nM), Birinapant (final concentration 100 nM), and Z-VAD-FMK (final concentration 20 μM) was added to induce necroptosis, the cells incubated for 7 h, and harvested.

Cells were transferred to a microcentrifuge tube and centrifuged at 200*g* for 5 min, and media were removed. Cells were washed with PBS and lysed with 100 μL of lysis buffer (radioimmunoprecipitation assay lysis buffer, Sigma-Aldrich, R0278) supplemented with 1× phosphatase inhibitor cocktail 2 (Sigma, P5726) and 1× complete EDTA-free protease inhibitor cocktail (Sigma, R0278)) on ice for 30 min. The lysate was collected by centrifugation at 17,000*g*, 4 °C for 5 min, and the lysate was transferred to a new microcentrifuge tube. Protein concentration was determined using the DC Protein Assay (Bio-Rad) in a 96-well plate as per the manufacturer’s instructions and subsequently adjusted to 1 mg mL^–1^ using lysis buffer. Samples were prepared by adding 4 μL of 4× loading buffer (Biorad, 161-0747) containing β-mercaptoethanol to 12 μL of protein sample and boiling at 95 °C for 10 min. 14 μL of each sample was loaded onto a 4–15% Mini-PROTEAN TGX precast gel (Biorad, 456-1086) and run in 1× running buffer (0.25 M Tris, 0.2 M glycine, 0.1% (w/v) SDS) for 1.15 h at 130 V. Either 2 μL of precision plus protein all blue prestained protein standards (Bio-Rad, 1610373) or precision plus protein dual color standards (Bio-Rad, 1610374) was used in at least one well.

Proteins were transferred onto a nitrocellulose membrane (GE Healthcare) using the Trans-Blot Turbo Transfer System (Biorad, 1704150) at 2.5 A and 25 V for 7 min. The membrane was stained with Ponceau S to confirm protein transfer prior to blocking with 5% (w/v) nonfat dried skimmed milk in TBS-T (1× Tris-buffered saline, 0.1% (v/v) Tween-20) at rt for 1 h. Staining with the desired primary antibody in 5% (w/v) nonfat dried skimmed milk in TBS-T was performed overnight at 4 °C. The blot was washed with TBS-T at rt (3 × 5 min) and stained with secondary antibody in 5% (w/v) nonfat dried skimmed milk in TBS-T for 1 h at rt. The blot was washed with TBS-T at rt (3 × 5 min), visualized with Immobilon Crescendo Western HRP substrate, and imaged using ImageQuant LAS-4000 Imaging System (Fujifilm), and the contrast normalized in Fiji.

### ADP Glo Kinase Assay

Compound potency and mode of inhibition against RIPK1 kinase activity were determined by using an ADP-Glo kinase assay (Promega). The assay was carried out in accordance with the manufacturer’s protocol. The initial kinase reaction was performed by incubating recombinant hRIPK1 (GST-Th-1-327 from BPS Bioscience ref 40371, 75 nM) with the inhibitor (0–100 μM) and ATP (0–500 μM) for 4 h at 24 °C in kinase assay buffer (50 mM HEPES pH 7.5, 50 mM NaCl, 30 mM MgCl_2_, 0.5 mg mL^–1^ BSA, 0.02% CHAPS, and 4 mM DTT). Following this reaction, ADP-Glo Reagent and Kinase Detection Reagent were successively added and incubated for 1 and 0.5 h at 24 °C, respectively. A 2:2:1 (kinase assay reaction/ADP-Glo Reagent/Kinase Detection Reagent) volume ratio of the kit’s components was used. Bioluminescence was measured on a Varioskan LUX multimode microplate reader with SkanIt Software. Experiments were conducted in triplicate. Concentration response curves and IC_50_ values were generated from nonlinear regression fits performed on GraphPad Prism 9.1.1. Additionally, the produced ADP concentration values were divided by the kinase reaction incubation time to calculate reaction velocity values and generate velocity curves. Velocity curves were fitted in Michaelis–Menten model on GraphPad Prism 9.1.1 to calculate the maximum velocity (*v*_max_) and Michaelis–Menten constant (*K*_M_) parameters.

### Streptavidin Shift Assay

I2.1 cells were seeded in sterile six-well plates at a density of 2 × 10^6^ cells per well and incubated at 37 °C overnight. 1000× stocks of the desired concentration of compound in DMSO were preprepared. Cells were treated with either the parent compound or DMSO with a final concentration of 0.1% DMSO and incubated for 1 h before treating with probe and further incubating for 3 h. TNF (final concentration 575 pM) was added to induce necroptosis and the cells incubated for 1 h before irradiation with UV light (365 nm) at various time points or kept in the dark before being harvested.

Cells were transferred to a microcentrifuge tube, centrifuged at 200*g* for 5 min, and media removed. Cells were washed with PBS and lysed with 100 μL of lysis buffer (as detailed before) on ice for 30 min. The lysate was collected by centrifugation at 17,000*g*, 4 °C for 5 min and the lysate transferred to a new microcentrifuge tube. Protein concentration was determined using the DC Protein Assay (Bio-Rad) in a 96-well plate as per the manufacturer’s instructions and the concentration adjusted to 1–2 mg mL^–1^ using lysis buffer.

The “click mixture” was prepared by combining a final concentration of 100 μM AzTB, 1 mM CuSO_4_, 1 mM TCEP, and 100 μM TBTA and incubated for 2 min at rt. 6 μL of the click mixture was added to every 100 μL of lysate. The reaction mixtures were shaken at rt for 1 h before being quenched with EDTA (500 mM EDTA in H_2_O, to a final concentration of 5 mM). Proteins were precipitated in ACN (4 vol) and briefly vortexed. The mixture was centrifuged at 10,000*g*, 4 °C for 5 min, and the supernatant was gently removed. The pellet was washed with 80% (v/v) EtOH (10 vol) and centrifuged at 16,000*g*, 4 °C for 2 min; the EtOH was removed, and the process was repeated for a total of three washes. The pellet was resuspended in 1% SDS in PBS, prior to dilution with PBS to a final concentration of 1–2 mg mL^–1^ of protein in 0.2% SDS.

Samples were prepared by adding 4 μL of 4× loading buffer to 10 μL of the protein samples and boiling at 95 °C for 10 min. 2 μL of either Streptavidin (100 μM in H_2_O) or Milli-Q H_2_O was added to the sample and shaken at rt for 10 min. 14 μL of sample was loaded onto a gel and run as previously described. In-gel fluorescence was detected by using a Typhoon FLA 9500 biomolecular imager (750 V, 100 μm pixels). Proteins were transferred onto a nitrocellulose membrane followed by staining with primary and secondary antibodies as previously described. The blot was visualized with Immobilon Crescendo Western HRP substrate and imaged using an ImageQuant LAS-4000 Imaging System (Fujifilm), and the contrast normalized in Fiji.

### Generation of Recombinant Kinase Domain of Receptor-Interacting Kinase 1 for Surface Plasmon Resonance

Biotinylated recombinant human kinase domain of RIPK1 [RIPK1 (8-322)-Avi-His] was produced in Sf21 cells using baculovirus infection (Bac-to-Bac system, Thermo Fisher Scientific). Briefly, the RIPK1 (8-322)-Avi-His baculovirus was inoculated into Sf21 cells at 1.5 × 10^6^ cells/mL, with 1 mL of virus added per 500 mL of cells for P2 virus generation. The cells were grown at 27 °C, 140 rpm for 72 h. RIPK1 was co-expressed with Cdc37 at a 4:1 (RIPK1/Cdc37) v/v virus ratio in Sf21 cells by inoculation with P2 virus (25 mL per 500 mL bottle). The cells were grown at 27 °C while shaking at 140 rpm for 48 h before being harvested by centrifugation at 6500 rpm and 4 °C for 15 min and stored at −80 °C before further purification. 25 μM of Nec-1s (ab221984, Abcam) was added to the culture medium at time of viral infection to stabilize protein.

The frozen pellet was resuspended in ice cold lysis buffer (40 mM HEPES pH 7.5, 1 M NaCl, 0.5 mM NaF, 10 mM β-glycerolphosphate, 50 μg/mL phenylmethanesulfonyl fluoride (PMSF), Benzonase 25 U/mL, 20 mM imidazole, 25 μM Nec-1s) and clarified by centrifugation at 24,000 rpm, 4 °C for 2 h. The supernatant was collected and purified on two Ni NTA Superflow affinity columns (Qiagen) using the KTA system equilibrated with wash buffer (50 mM Tris at pH 7.6, 1 M NaCl, 20 mM imidazole, 25 μM Nec-1s, 0.25 mM TCEP, and 1× complete protease inhibitor) and eluted with 250 mM imidazole. The eluted protein was further purified by size exclusion chromatography (Superdex 75, KTA, Cytiva) in SEC running buffer (25 mM Tris pH 8.0, 150 mM NaCl, 5 mM DTT, 25 μM Nec-1s) and biotinylation using BirA. The biotinylated protein was passed down a PD10 column to remove excess biotinylation components and changed to storage buffer (25 mM Tris pH 8, 100 mM NaCl, 2 mM TCEP, 25 μM Nec-1s). The protein was finally concentrated to 3.8 mg mL^–1^, snap-frozen in LN_2_, and stored in −80 °C.

### Surface Plasmon Resonance Binding Assay

Peptide affinities to the kinase domain of RIPK1 was determined in a direct binding assay using 8K or S200 SPR biosensor (Cytiva) at 20 °C. Briefly, RIPK1 kinase domain was immobilized on a streptavidin-coated sensor chip (Cytiva). The surface was washed with 10 mM NaOH, 1 M NaCl, followed by immobilization of the protein. Immobilization levels were typically 6000 RU. The reference spot was treated as described, omitting the injection of RIPK1. Compound concentration series was injected over the immobilized protein in increasing concentrations using multi cycle or single cycle kinetics in running buffer (10 mM HEPES, 150 mM NaCl, 0.05% Tween20, 0.1% DMSO, pH 7.4). A 1:1 Langmuir interaction model was fitted to the experimental traces, enabling determination of *k*_on_, *k*_off_, and *K*_D_.

### Expression of Recombinant Receptor-Interacting Kinase 1 Bound to Necrostatin 1 for X-Ray Crystallography and Hydrogen–Deuterium Exchange

Human recombinant RIPK1 residues 1–294 (Uniprot ID Q13546) with C34, C127, C233, and C240 mutated to alanine and N-terminal 6xHis-tag followed by TEV cleavage site was cloned into pFastBac1 vector (Thermo Fisher Scientific). Hsp90 co-chaperone Cdc37 in pFastBac1 vector was used for co-expression to facilitate correct folding of protein. Recombinant baculovirus was generated using a Bac-to-Bac system (Thermo Fisher Scientific). Bacmids were produced in DH10Bac cells, and virus amplification was carried out in ExpiSf9 cells cultured in ExpiSf CD medium (ExpiSf expression system, ThermoFisher Scientific). RIPK1 was co-expressed with Cdc37 at a 4:1 (RIPK1/Cdc37) v/v ratio using a multiplicity of infection (MOI) of 2 in ExpiSf9 cells for 48 h. 25 μM Nec-1s (ab221984, Abcam) was added to the culture medium at the time of viral infection to stabilize protein.

The cells were harvested by centrifugation, and the pellets were resuspended in lysis buffer (40 mM HEPES, 1 M NaCl, 20 mM imidazole, 2 mM TCEP, 25 μM Nec-1s, 1× complete protease inhibitor) and lysed through sonication for 2 × 20 s (Branson Sonifier 450, 80% duty cycle, Output Control 9). The lysate was cleared by centrifugation at 16,000 rpm, 4 °C for 1 h. The cleared lysate was purified by nickel affinity chromatography (HisTrap Crude FF, Cytiva) and eluted with 250 mM imidazole. The protein was cleaved with TEV (1:20 ratio) and dialyzed overnight at 4 °C. The protein was further purified by anion-exchange chromatography (5/50 GL MonoQ, Cytiva) and gel-filtration chromatography (Superdex75, Cytiva). The purified RIPK1 was stored in a buffer containing 25 mM Tris (pH 8.0), 150 mM NaCl, 2 mM TCEP, and 25 μM Nec-1s.

### Crystallization of **AZ’902** and **AZ’320** Bound to Receptor-Interacting Kinase 1

RIPK1 ((1–294) [C34A, C127A, C233A, C240A]) in complex with Nec-1s was concentrated to 11.5 mg mL^–1^ and crystallized in sitting drops at 20 °C by mixing a 1:1 ratio of protein solution with well solution containing 20% PEG4000, 0.3 M NaCl, and 0.1 M MES pH 6.5. The structure was obtained by soaking crystals with 10 mM compound for 90 h before being cryoprotected in 20% glycerol and flash frozen in liquid nitrogen. Data were collected at the BioMAX beamline at MAX IV in Lund, Sweden. The structures were determined using molecular replacement using Molrep and an internal search model. Refinement was carried out using refmac, and model building was carried using Coot. Refinement was carried out to 2.26 and 2.15 Å resolution having a Rwork and Rfree of 0.22 and 0.29 for **AZ’902** and 0.25 and 0.29 for **AZ’320**, respectively. For further statistics, see Table S1.

### Hydrogen–Deuterium Exchange Buffer Dialysis

Prior to HDX, the protein was dialyzed into the relevant buffer system. Briefly, the dialysis membrane (10K MWCO, Thermo Scientific) was prewet with distilled water. RIPK1 was loaded into the sealed dialysis tubing and immersed in a sterile dialysis buffer (25 mM Tris, 150 mM NaCl, and 2 mM TCEP, pH 8.0). The dialysis buffer was stirred overnight at 4 °C and then replaced with fresh buffer and stirred for another 3 h. The sample was recovered and concentrated by using a 30 kDaA MWCO concentrator (Vivaspin). The protein concentration was determined by NanoDrop One (Thermo Scientific) and the protein snap-frozen in LN_2_ and stored at −80 °C.

### Hydrogen–Deuterium Exchange Mass Spectrometry

Individual complexes (10 μM) were incubated with 40 μL of D_2_O buffer at rt for 3, 30, 300, and 3000 s in triplicate. The labeling reaction was quenched by adding chilled 2.4% v/v formic acid in 2 M guanidinium hydrochloride and immediately frozen in LN_2_. Samples were stored at −80 °C prior to analysis.

The quenched protein samples were rapidly thawed and subjected to proteolytic cleavage by pepsin, followed by reversed phase HPLC separation. Briefly, the proteins were passed through an Enzymate BEH immobilized pepsin column, 2.1 mm × 30 mm, 5 μm (Waters, UK) at 200 μL/min for 2 min and the peptic peptides trapped and desalted on a 2.1 mm × 5 mm C18 trap column (Acquity BEH C18 Van-guard precolumn, 1.7 μm, Waters, UK). Trapped peptides were subsequently eluted over 12 min using a 5–36% gradient of acetonitrile in 0.1% (v/v) formic acid at 40 μL/min. Peptides were separated on a reverse phase column (Acquity UPLC BEH C18 column), 1.7 μm, 100 mm × 1 mm (Waters, UK). Peptides were detected on a SYNAPT G2-Si HDMS mass spectrometer (Waters, UK) acquiring over *m*/*z* of 300–2000, with the standard ESI source and lock mass calibration using [Glu1]-fibrino peptide B (50 fmol/μL). The mass spectrometer was operated at a source temperature of 80 °C and a spray voltage of 3.0 kV. Spectra were collected in positive ion mode.

Peptide identification was performed by MS^e^ using an identical gradient of increasing acetonitrile in 0.1% (v/v) formic acid over 12 min. The resulting MS^e^ data were analyzed by using Protein Lynx Global Server software (Waters, UK) with an MS tolerance of 5 ppm.

Mass analysis of the peptide centroids was performed using DynamX software (Waters, UK). Only peptides with a score >6.4 were considered. The first round of analysis and identification was performed automatically by the DynamX software; however, all peptides (deuterated and non-deuterated) were manually verified at every time point for the correct charge state, presence of overlapping peptides, and correct retention time. Deuterium incorporation was not corrected for back-exchange and represents relative, rather than absolute changes in deuterium levels. Changes in H/D amide exchange in any peptide may be due to a single amide or a number of amides within that peptide. All time points in this study were prepared at the same time, and individual time points were acquired on the mass spectrometer on the same day.

### Synergy Assay

I2.1 cells were seeded in a sterile 96-well plate at a density of 2 × 10^5^ cells per well and incubated at 37 °C overnight. The cells were treated with a final concentration of 600 pM TNF, 1 μM Sytox Green, and the relevant concentrations of **AZ’902** and Nec-1s. Wells containing TNF and Sytox Green served as a positive control. Wells containing DMSO and Sytox Green served as a negative control. The plate was placed into an IncuCyte S3 Live Cell Analysis System (Sartorius), and readings for phase and green fluorescence were taken every 15 min for 24 h. The images were analyzed with IncuCyte 2021C software, the metric GP at *t* = 5 h was selected, and the background at time = 0 h was subtracted. The data from each replicate (*n* = 3) were normalized to the positive and negative controls analyzed for synergism using Combenefit software.

### In Vivo Studies

Male C57BL/6J mice of 18–25 g were used at the age of 6–8 weeks. Three animals were included in each experimental group. All animal experiments were carried out with the permission of the local animal ethical committee in accordance with the EU Directive (2010/63/EU), Portuguese laws (DL113/2013, 2880/2015, 260/2016), and all relevant legislations. Animals received humane care in a temperature-controlled environment with a 12 h light–dark cycle and *ad libitum* access to pelleted chow and water, complying with the Institute’s guidelines, and as outlined in the “Guide for the Care and Use of Laboratory Animals” prepared by the National Academy of Sciences and published by the National Institutes of Health (NIH publication 86-23 revised 1985). To validate the anti-necroptotic potential of a test compound on TNF-induced SIRS, mice were challenged with TNF in the presence and absence of **AZ’320**, while Nec-1 posed as positive control for TNF-induced SIRS protection. TNF was diluted in sterile PBS. Nec-1 and **AZ’320** were diluted as follows (v/v): 44.5% PBS 1×, 17% DMSO, 10% ethanol, 2.5% cyclodextrin, 18% PEG 400, and 8% cremophor. Vehicle control, **AZ’320** or Nec-1 (125 μg; 5 mg/kg body weight), was injected 15 min before TNF challenge (0.5 mg/kg body weight). All injections were administered intravenously (i.v.).

## Supplementary Material



## Notes

Uncropped immunoblots and gels; synthetic methods for intermediates and final compounds; kinase panel screen method; details of X-ray crystal structures; HPLC chromatograms; and NMR spectra (DOCX)

## Notes

PDB ID codes: 9GTG (RIPK1 cocrystallized with **AZ’902** and Nec-1s); 9GTY (RIPK1 cocrystallized with **AZ’320** and Nec-1s). Authors will release the atomic coordinates and experimental data upon article publication.

## Abbreviations Used

TNF: tumor necrosis factor alpha
RIPK: receptor-interacting serine/threonine-protein kinase
MLKL: mixed lineage kinase domain-like protein
DAMPs: damage-associated molecular patterns
Nec-1: Necrostatin 1
NSA: Necrosulfonamide
7PQ: 7-phenylquinoline
SPR: surface plasmon resonance
HDX: hydrogen–deuterium exchange
LHS: left-hand side
RHS: right-hand side
AzTB: azide-TAMRA-biotin
CuAAC: copper­(I)-catalyzed azide–alkyne cycloaddition
SIRS: systemic inflammatory response syndrome
