# Supplementary material for: Discovery and Validation of a Novel Class of Necroptosis Inhibitors Targeting RIPK1
Source: ACS Chem Biol. 2025 Jun 20;20(7):1527–43. doi: 10.1021/acschembio.5c00112 (PMC12281487; doi:10.1021/acschembio.5c00112)
Supplement: Supplementary file 1 [file cb5c00112_si_001.docx]

**Supporting Information for the manuscript Discovery and validation of a novel class of necroptosis inhibitors targeting RIPK1**

Lior Soday ^1,†^, Chotima Seripracharat ^1,†^, Janine L. Gray ^1^, André F.S. Luz ^2^, Ryan T. Howard ^1^, Ravi Singh ^1^, Thomas J. Burden ^1^, Erika Bernardini ^1^, Miguel Mateus-Pinheiro ^2^, Jens Petersen ^3^, Anders Gunnarsson ^3^, Jenny Gunnarsson ^3^, Anna Aagaard ^3^, Tove Sjögren ^3^, Sarah Maslen ^4^, Edward J. Bartlett ^1^, Abigail F. Iles ^1^, David M. Smith ^5^, James S. Scott ^5^, Mark Skehel ^4^, Andrew M. Davis ^5^, Ana S. Ressurreição ^2^, Rui Moreira ^2^, Cecília M.P. Rodrigues ^2^, Avinash R. Shenoy ^6^, Edward W. Tate ^1,4,*^

^1^ Department of Chemistry, Molecular Sciences Research Hub, Imperial College London, London, W12 0BZ, UK

^2^ Research Institute for Medicines (iMed.ULisboa), Faculty of Pharmacy, Universidade de Lisboa, Lisboa, Portugal

^3^ Discovery Sciences, R&D Gothenburg, AstraZeneca, Pepparedsleden 1, SE-431 83 Mölndal, Sweden

^4^ The Francis Crick Institute, London, NW1 1AT, UK

^5^ Hit Discovery, Discovery Sciences, R&D, AstraZeneca, Cambridge CB2 0AA UK

^6^ Department of Infectious Diseases, Flowers building, South Kensington Campus, Imperial College London, UK

† These authors contributed equally to this work

* Correspondence to: e.tate@imperial.ac.uk

**Supplemental Figures**


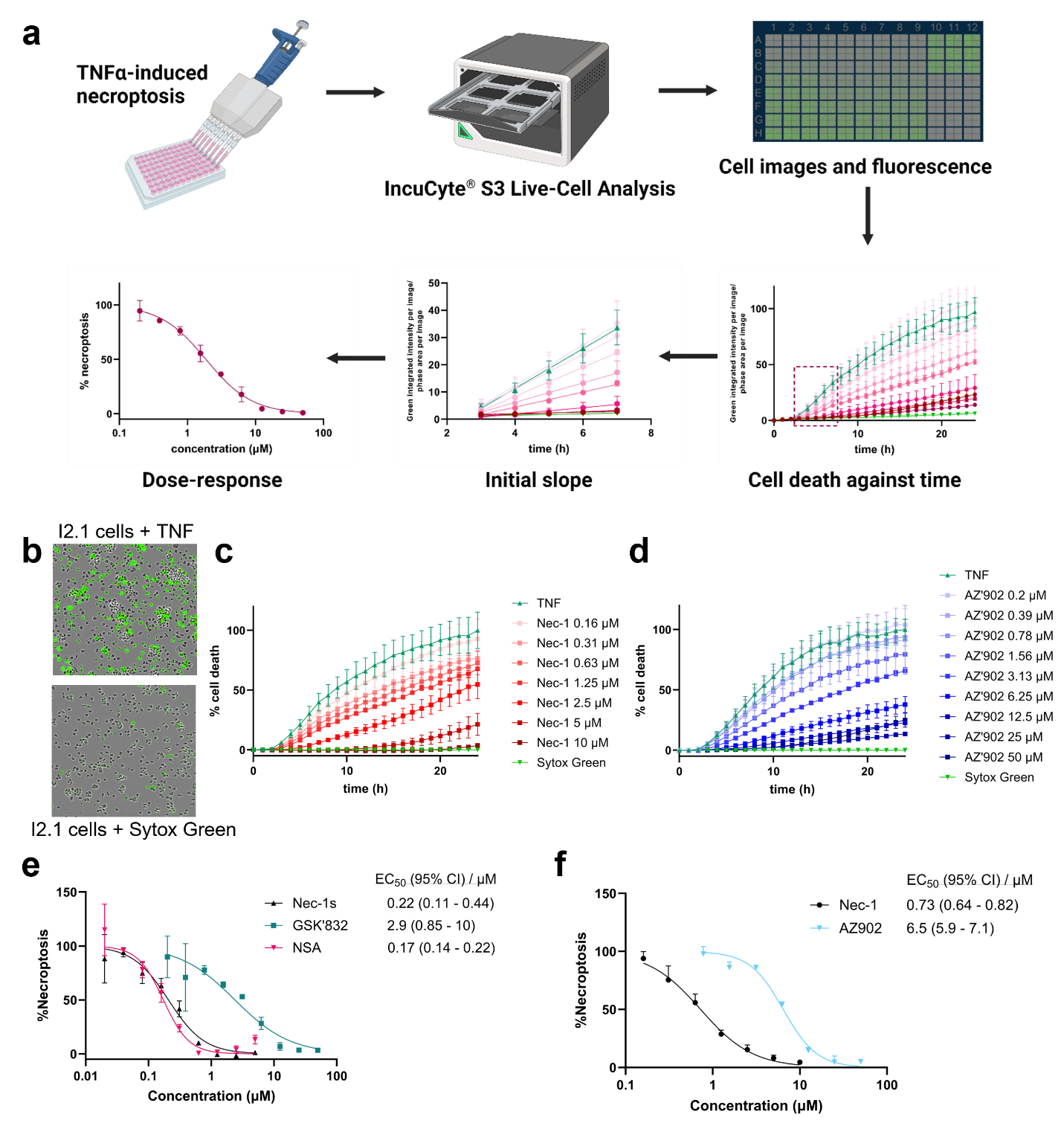


**Figure S1** **Novel IncuCyte® based necroptosis assay**

(**a**) Schematic overview of IncuCyte® based necroptosis assay. I2.1 cells in a 96-well plate were treated with TNF to induce necroptosis, a dilution series of the candidate necroptosis inhibitor and Sytox Green to monitor cell death. Plates were placed in the IncuCyte® and imaged every hour for 24 h. Cell death was measured over time and used to determine the proportion of necroptotic cells compared to those without necroptosis induction or not treated with an inhibitor. The linear portion of the graph was used to determine an initial rate for calculation of the EC_50_. TNF with Sytox Green and Sytox Green alone were used as positive and negative controls, respectively. (**b**) Phase and green fluorescence images of I2.1 cells taken from the IncuCyte® showing treatment with TNF and Sytox green (top) or with only Sytox green (bottom). (**c**) Cell death, as a percentage of total cells after treatment with TNF and varying concentration of Nec-1 over time shown as representative data from the Incucyte assay from which EC_50_s were determined. (**d**) Percentage cell death upon treatment with TNF and varying concentration of **AZ’902** over time, depicting representative data from the Incucyte assay from which EC_50_s were determined. (**e**) EC_50_ curves for known inhibitors of necroptosis in I2.1 cells from the Incucyte assay. EC_50_ values are expressed as the geometric mean with a 95% confidence interval (CI) of at least three biological replicates. Results are representative of at least three biological replicates; error bars represent SD (*n* = 3). (**f**) EC_50_ curves for Nec-1 and **AZ’902** in HT-29 cells. In this instance, the cells were treated with TNF, birinapant and Z-VAD-FMK to induce necroptosis. EC_50_ values were calculated from a single biological replicate; error bars represent SD from technical replicates (*n* = 3).


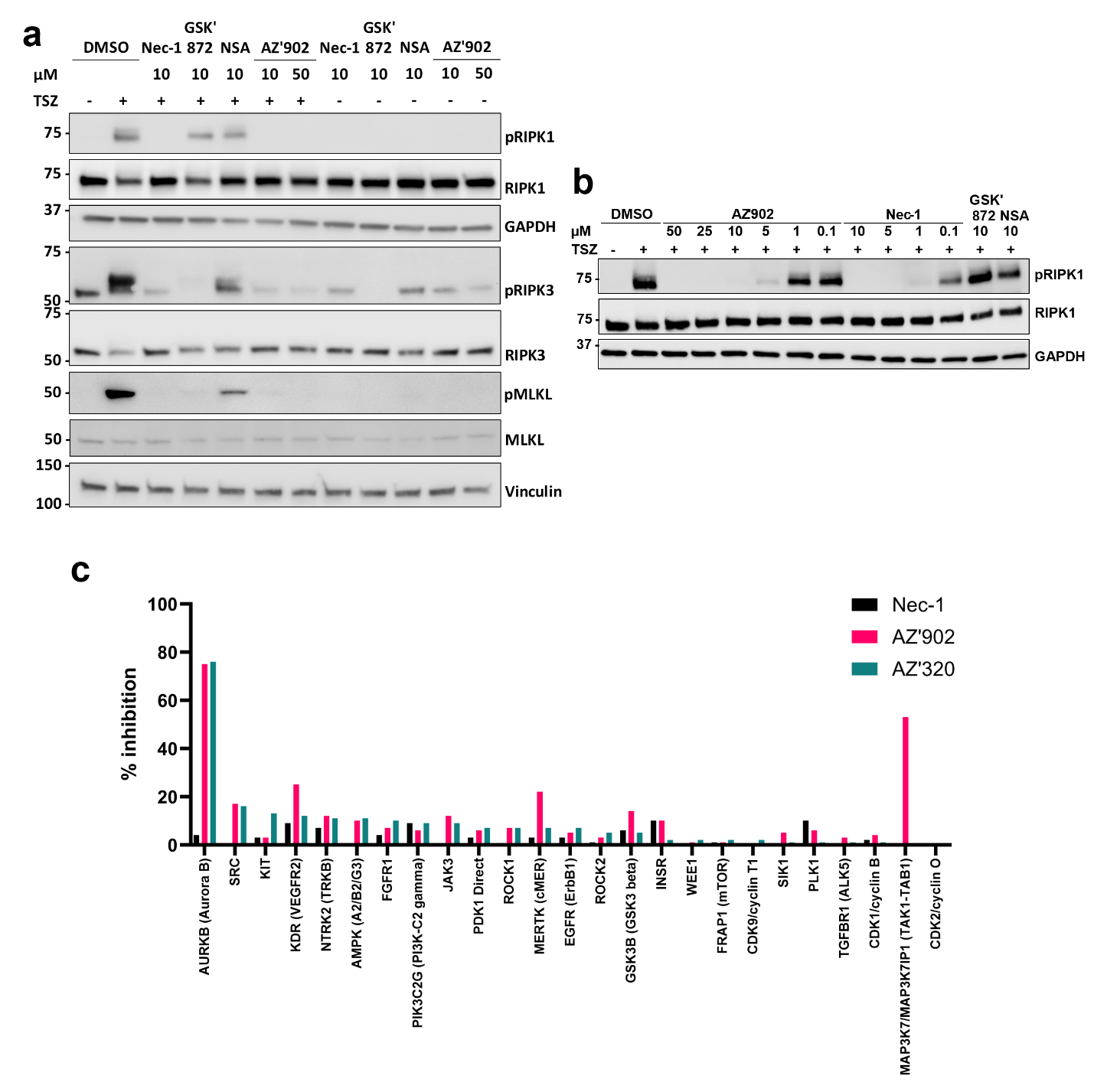


**Figure S2 Validation of the 7PQ series as inhibitors of RIPK1**

(**a**) Western blot analysis of RIPK1, RIPK3 and MLKL phosphorylation in HT-29 cells with treatment of necroptosis inhibitors and the 7PQ series. Results are representative of two experiments. (**b**) Western blot analysis of dose dependent inhibition of RIPK1 Ser166 phosphorylation in HT-29 cells by Nec-1 and **AZ’902**. Results are representative of two experiments. (**c**) Kinase activity panel screen. Compounds were screened at a single concentration of 1 µM at Thermofisher. Data shows % inhibition.

**
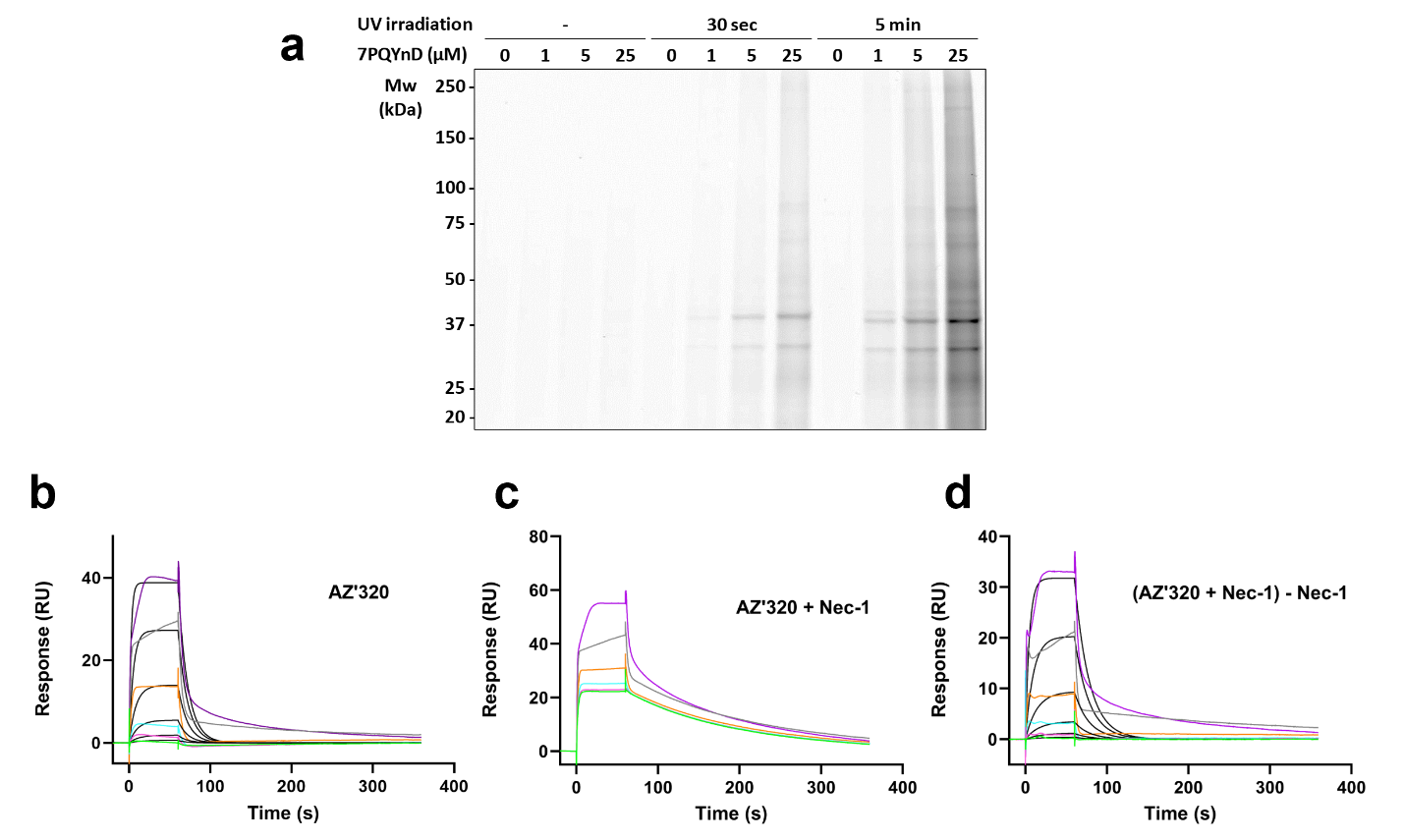
**

**Figure S3. 7PQ series binds to the kinase domain of RIPK1**

(**a**) Gel analysis of **7PQYnD** probe labelling of I2.1 cells. In-gel fluorescence was used to qualitatively assess TAMRA-tagged proteins following CuAAC of **7PQYnD** with Az-TB. (**b-d**) Representative SPR sensorgrams of increasing concentrations of **AZ’320** and Nec-1 (green, pink, teal, grey, orange, purple) and 1:1 fitting (black) of the interactions with the kinase domain of RIPK1.

**
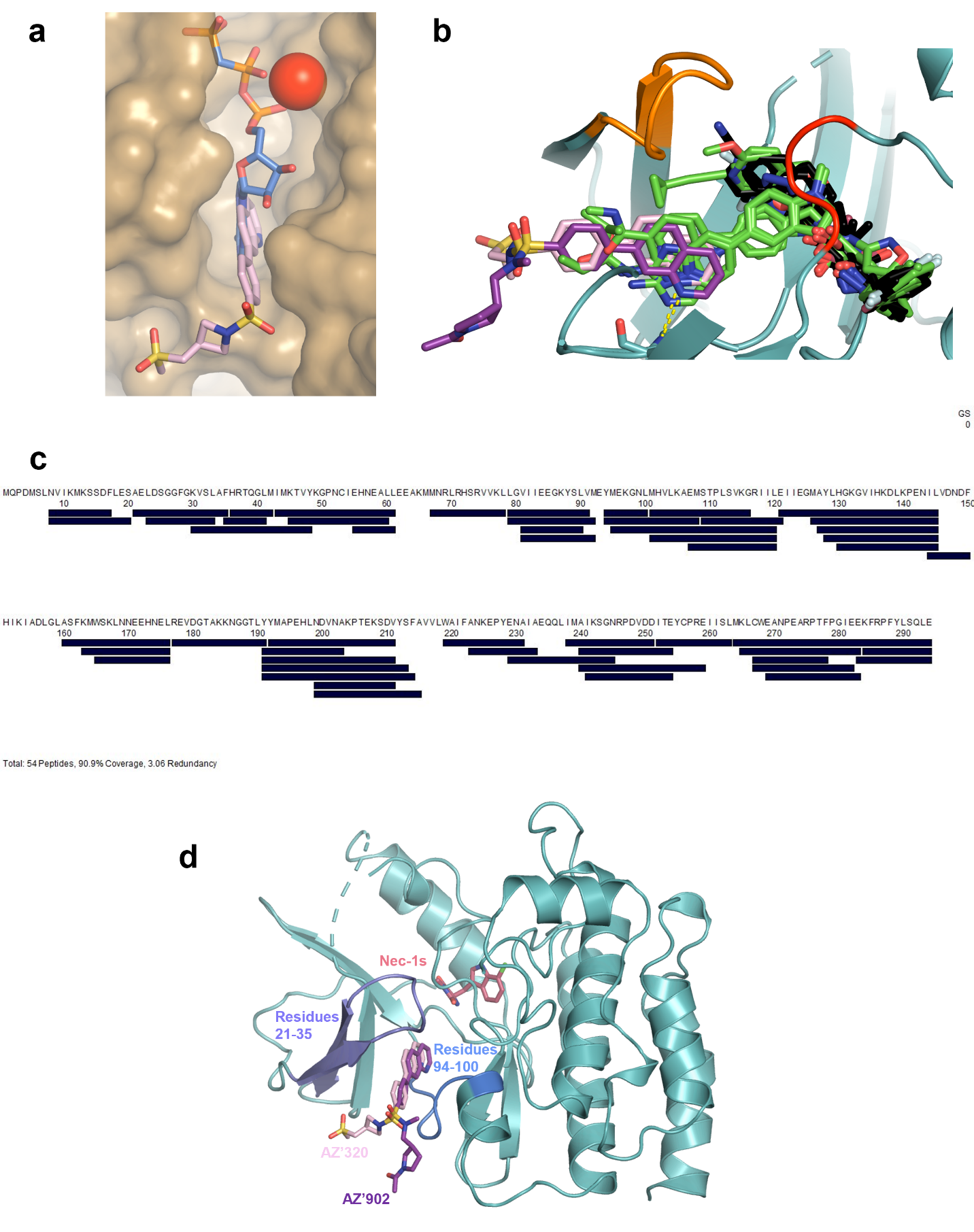
Figure S4. Characterization of 7PQ binding mode as Type I kinase inhibitors**

(**a**) Alignment of **AZ’320** (pink sticks) with ATP (blue sticks) bound to RIPK2 (PDB code: 5AR3). Mg^2+^ ion is shown as a red sphere. **AZ’320** binds in the hinge region, overlapping with the adenine ring of ATP, characterizing the 7PQ series as a Type I kinase inhibitor. (**b**) Comparison of Type II (green sticks) and Type III (black sticks) to **AZ’320** (pink sticks) and **AZ’902** (purple sticks) bound to RIPK1 (PDB codes: 4ITH, 8I2N, 5TX5, 5HX6, 7XMK, 4NEU, 7YDX, 7FCZ, 6C4D, 6HHO, 6NW2, 6NYH, 6OCQ, 6R5F, 6RLN, 9GTG, 9GTY). (**c**) Sequence coverage of RIPK1 during HDX-MS experiment. (**d**) Residues protected during the HDX-MS experiment are depicted on the RIPK1 (teal cartoon) co-crystal structure (PDB code = 9GTY) of Nec-1s (dark pink sticks) and **AZ’320** (pink sticks) aligned with **AZ’902** (purple sticks). Regions 21-35 and 94-100 are depicted as light purple and light blue cartoons, respectively. These areas align with the glycine rich loop and hinge region highlighted in Figure 4a.

**Figure S5. Uncropped Immunoblots and gels**

Red boxes indicate portions of the blots taken for figures.

Figure 2a


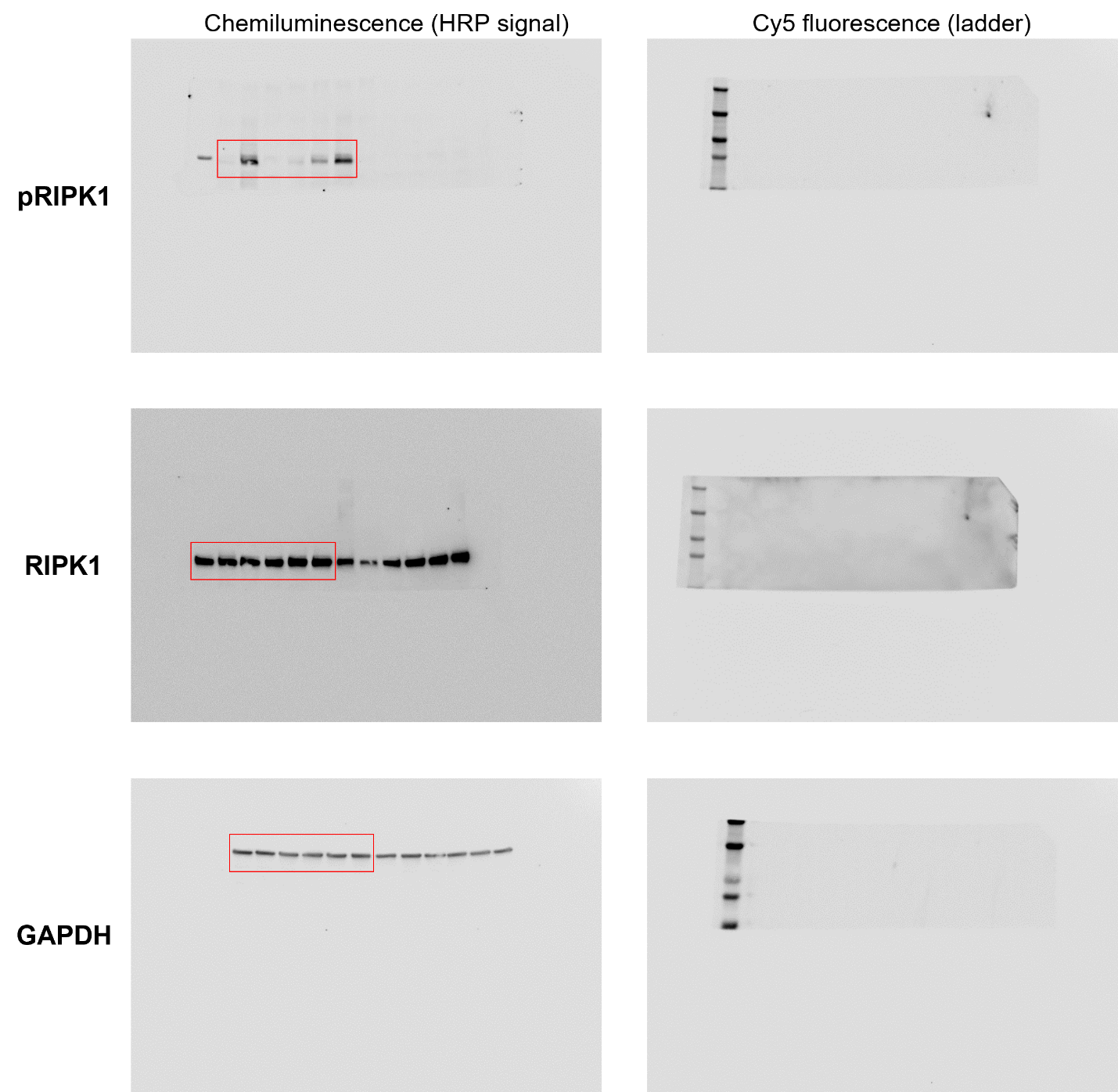


Figure 2b


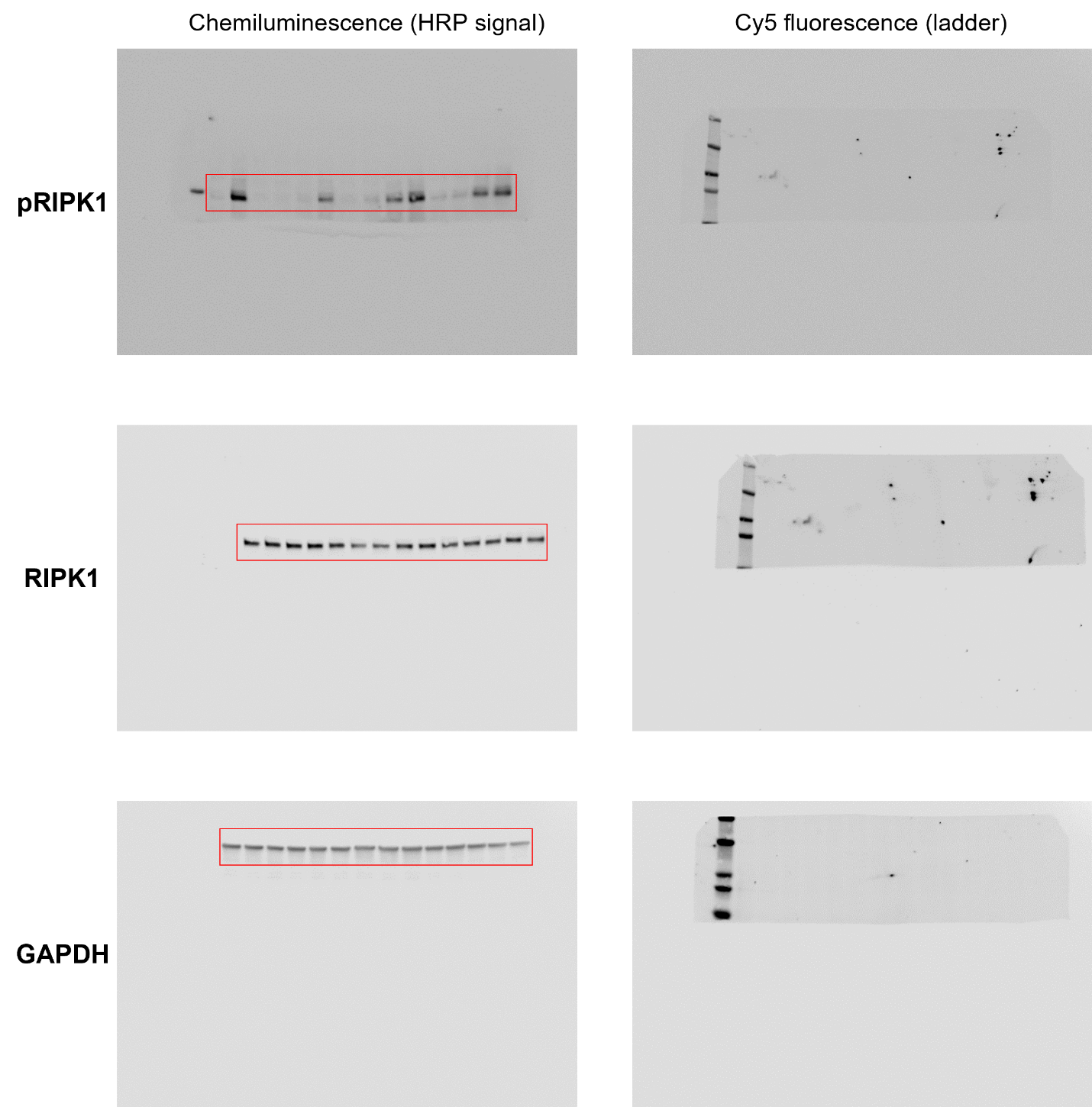


Figure S2a


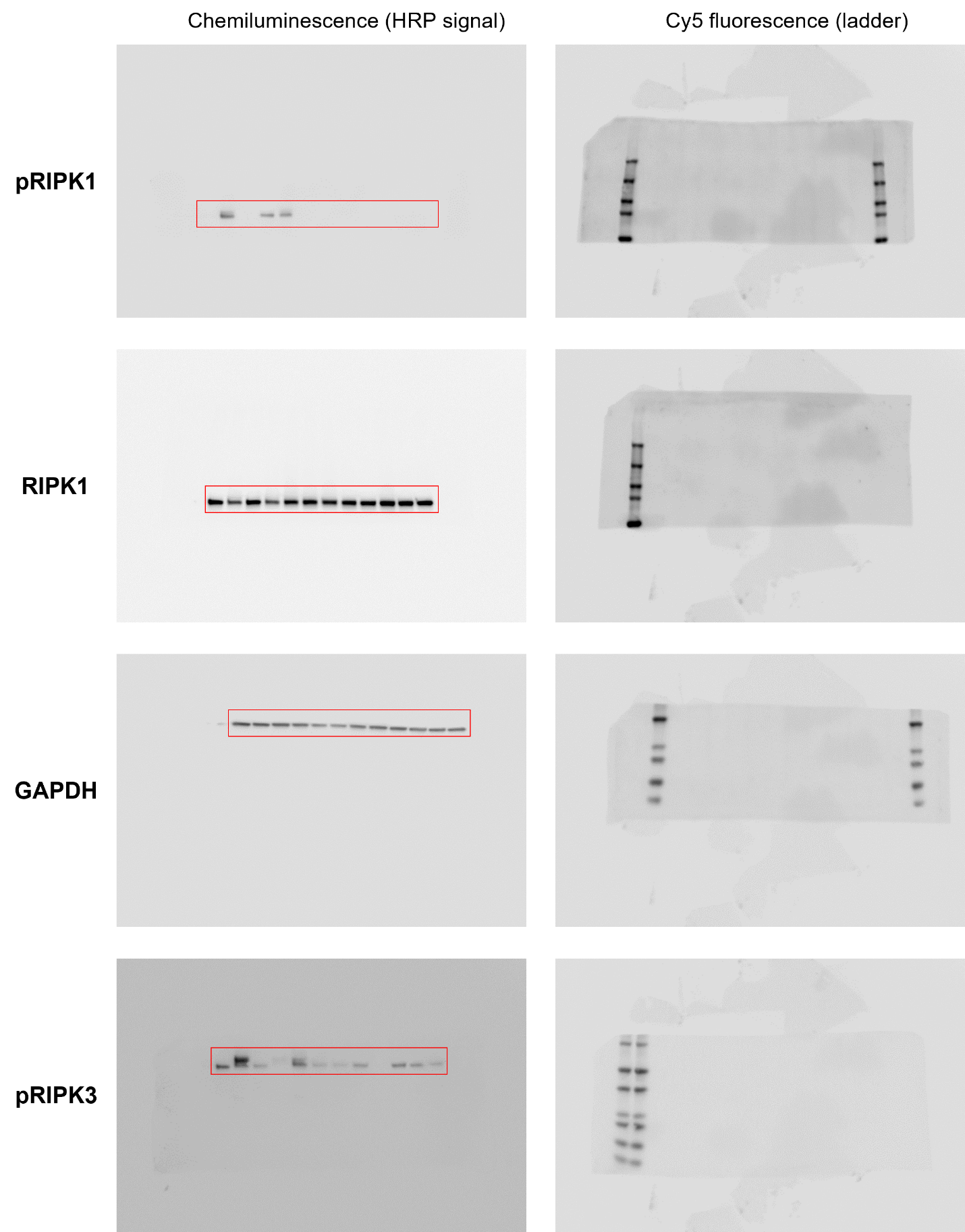


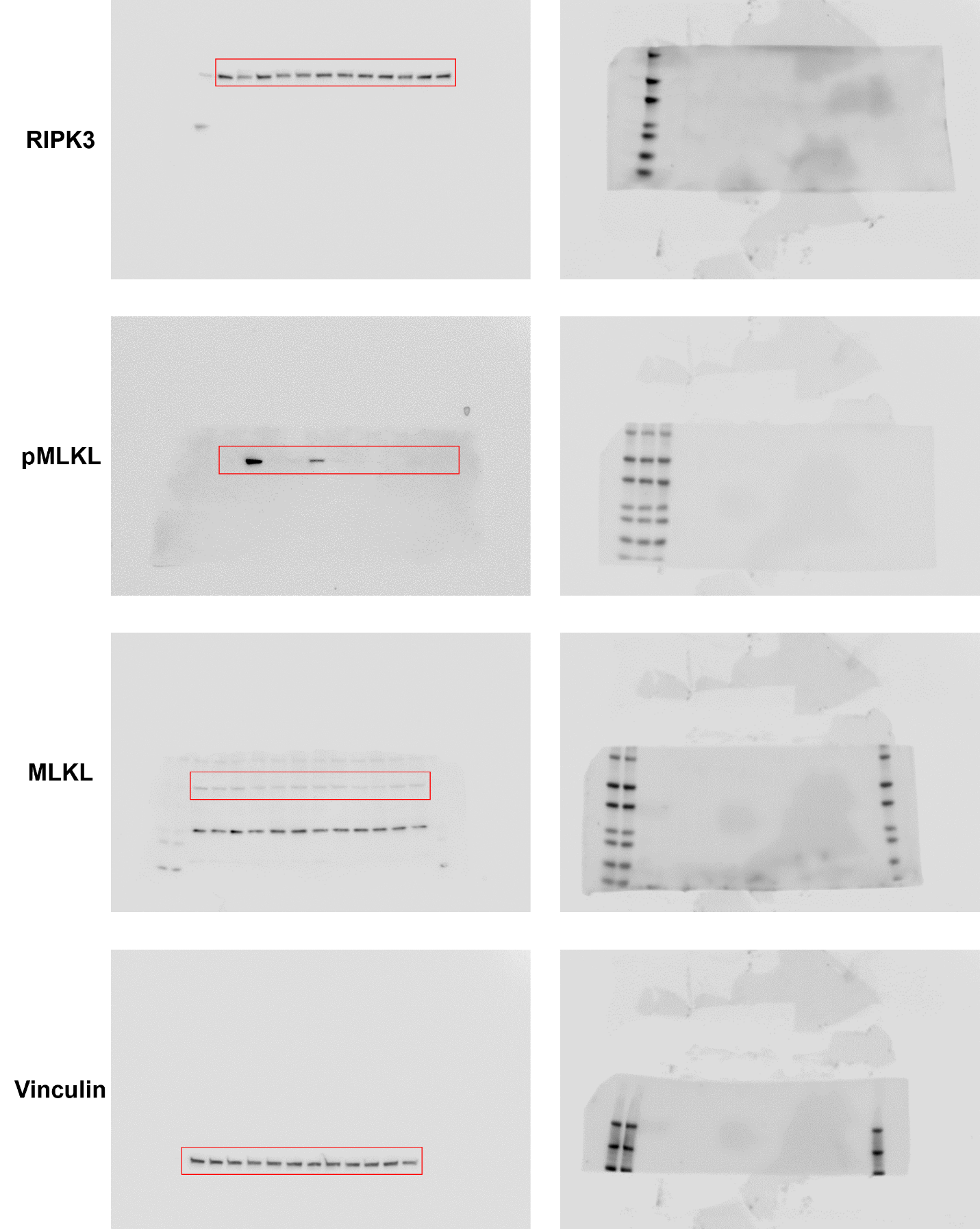


Figure S2b


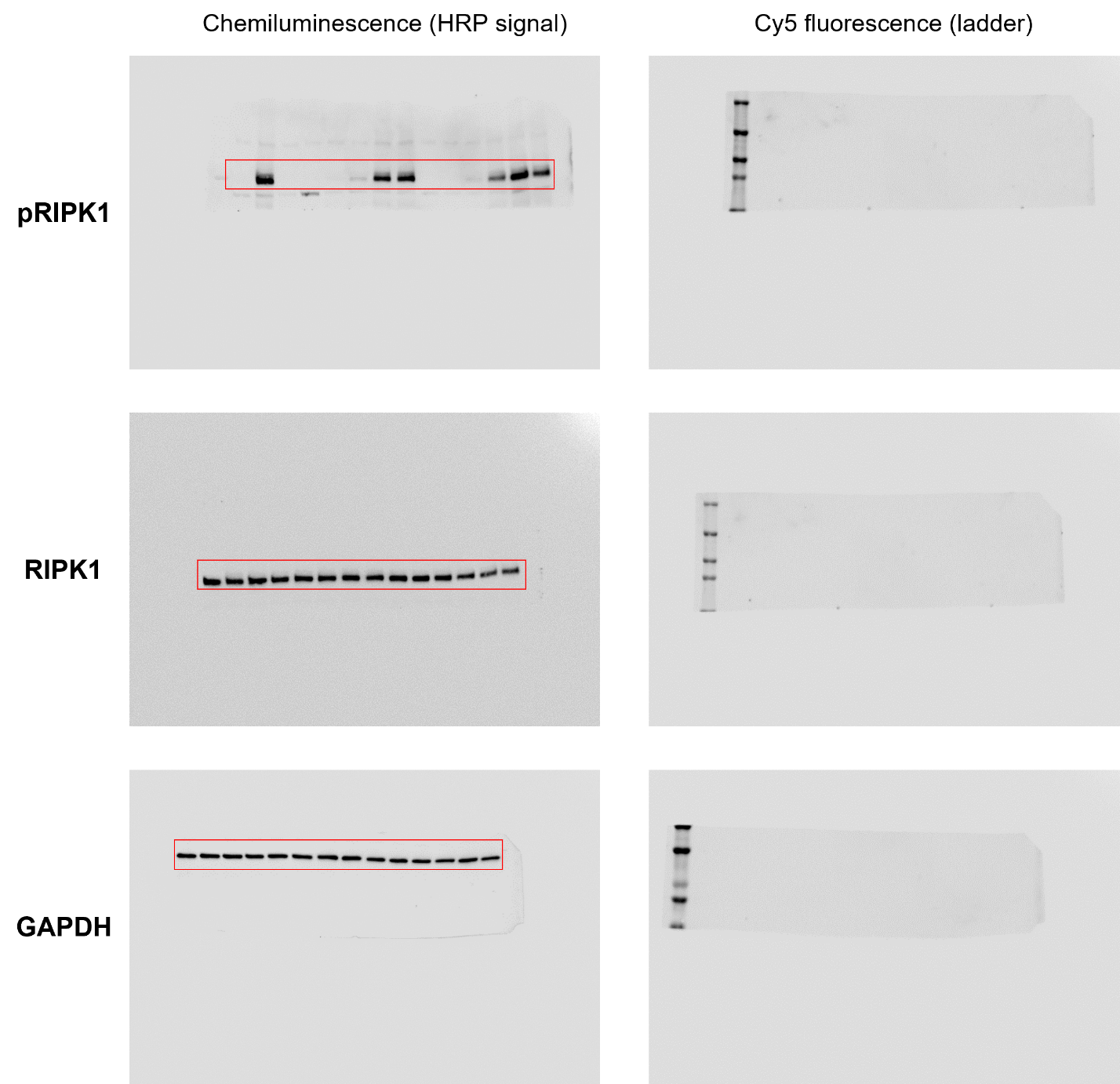


Figure 3b


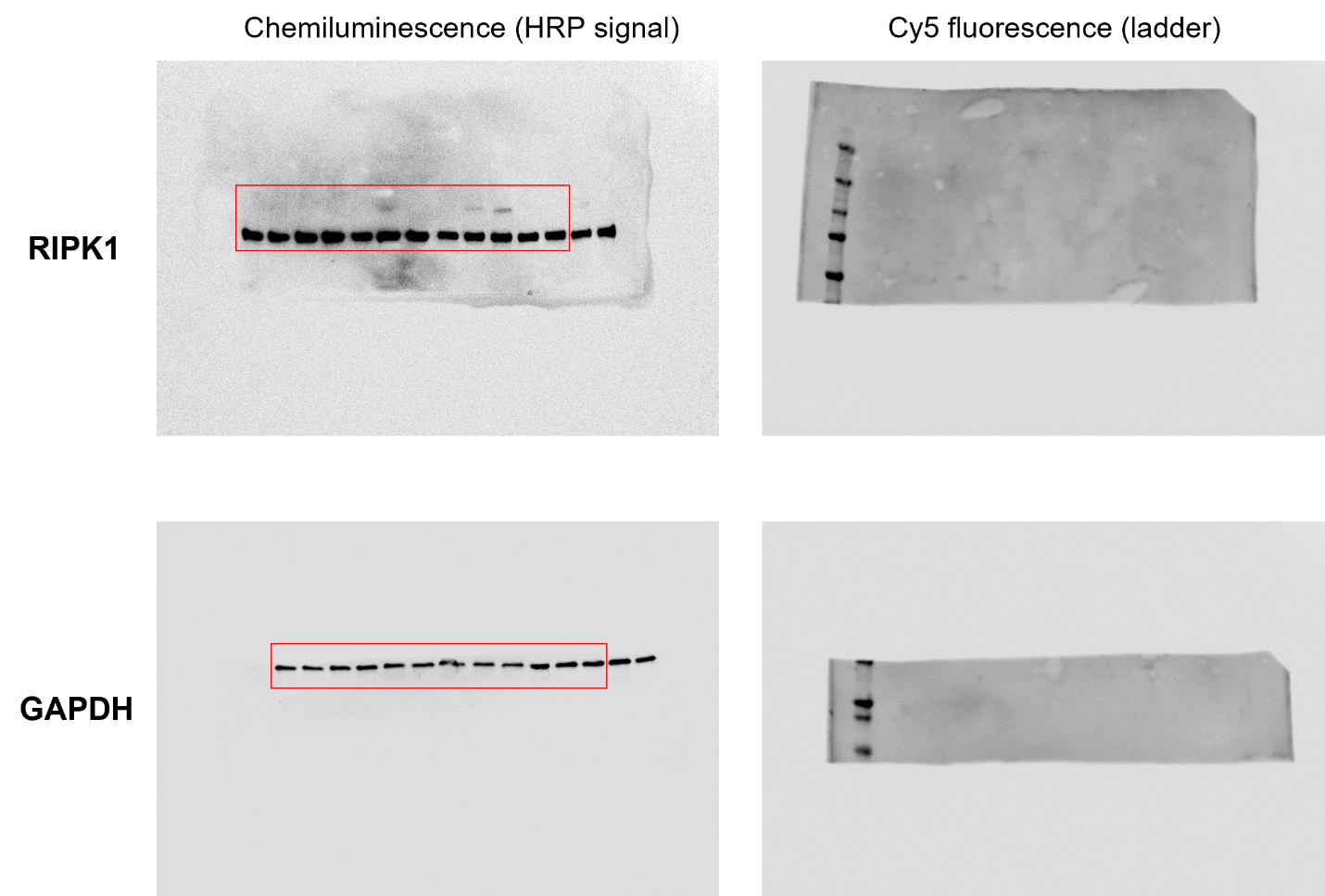


Figure 3c


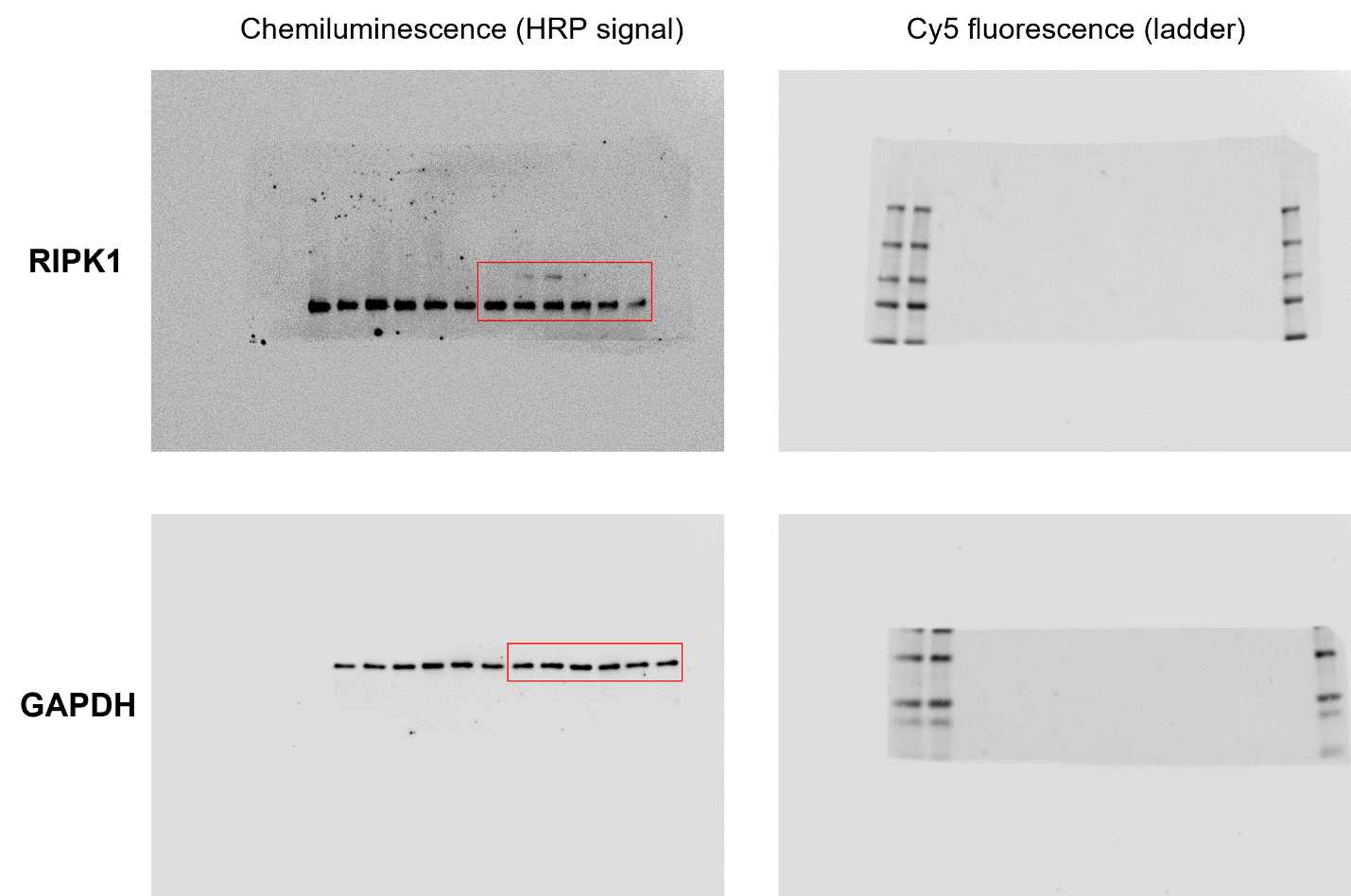


Figure 3d


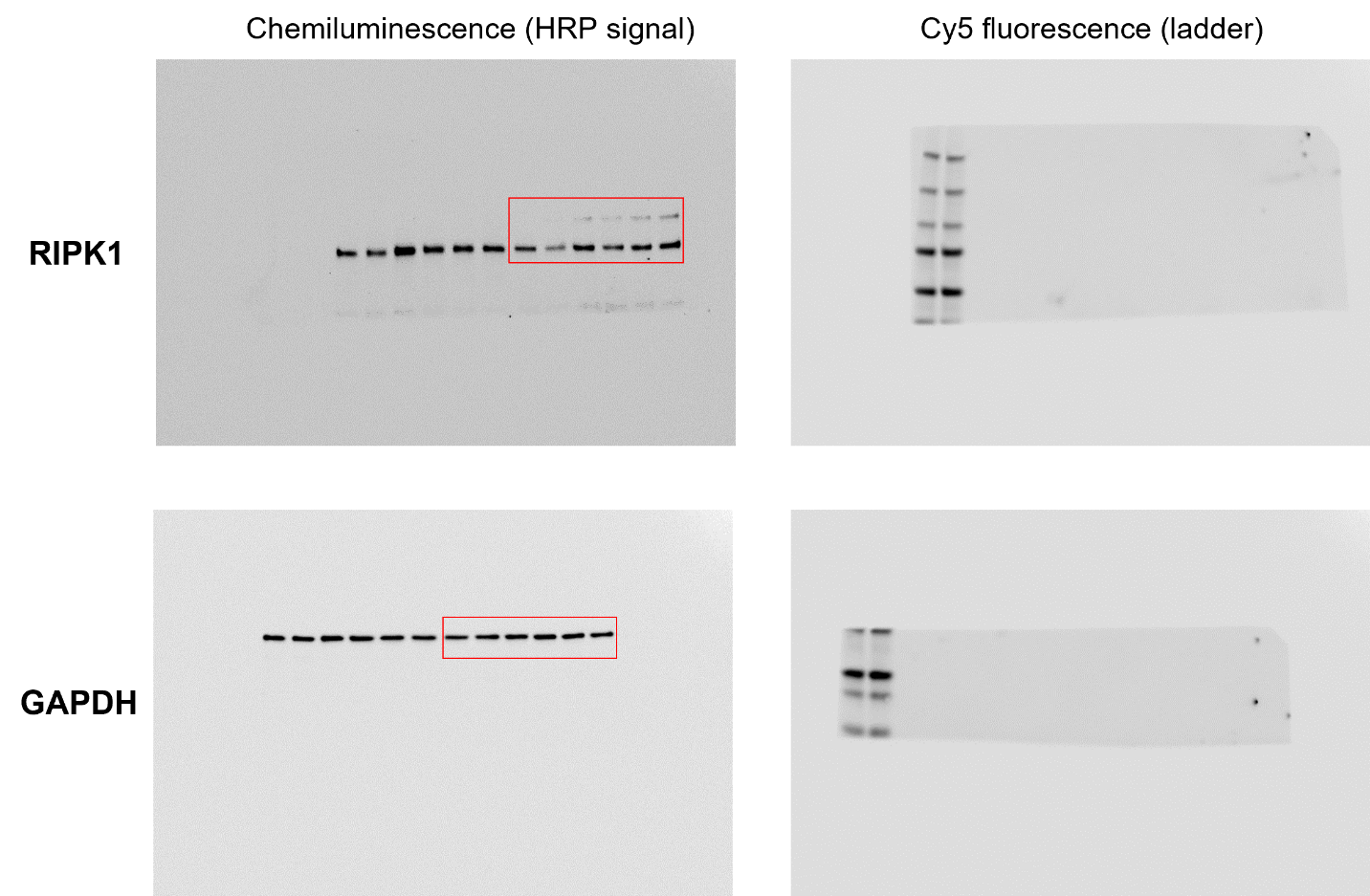


Figure S3a


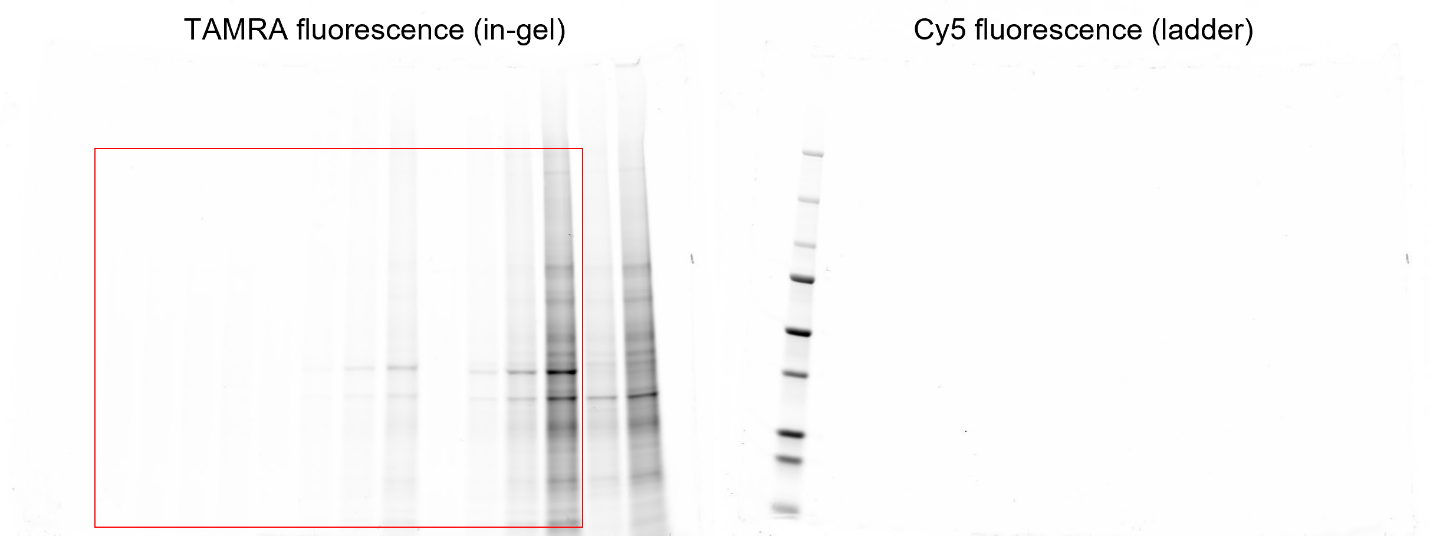


**Supplementary Methods**

**Synthesis of compounds**

**Synthesis of AZ'902**


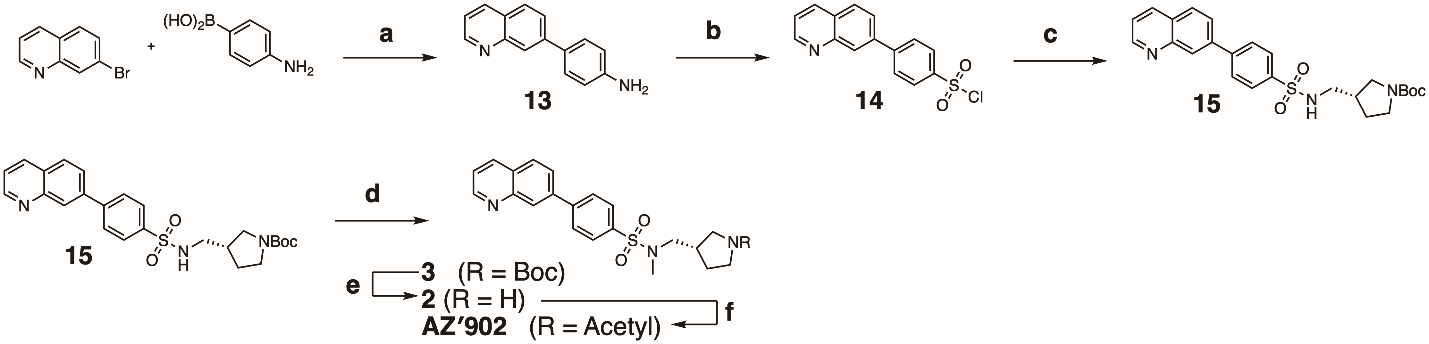


Reagents and conditions: (**a**) Pd(PPh_3_)_4_, Na_2_CO_3_, H_2_O/MeCN, 130 °C, 18 h, 93%; (**b**) (i) conc. HCl, -15 °C, (ii) NaNO_2_, H_2_O (iii) SOCl_2_, CuCl, H_2_O, -15 °C, 1.5 h, 25%; (**c**) NEt_3_, CH_2_Cl_2_, rt, 18 h, 86 %; (**d**) CH_3_I, K_2_CO_3_, DMF, rt, 4 h, 60%; (**e**) 4M HCl in dioxane, N_2_, rt, 2 h, 97%; (**f**) acetic anhydride (Ac_2_O), pyridine, CH_2_Cl_2_, rt, 2 h, 68%.

**4-(quinolin-7-yl)aniline (13)**

7-bromoquinoline (1.00 g, 4.81 mmol) and 4-(4,4,5,5-tetramethyl-1,3,2-dioxaborolan-2-yl)aniline (1.06 g, 4.82 mmol) were dissolved in MeCN (30 mL) and Na_2_CO_3_ (257 mg, 2.42 mmol) in H_2_O (15 mL) was added. Tetrakis(triphenylphosphine)palladium (166 mg, 0.143 mmol) was added, and the reaction mixture was refluxed at 130 °C overnight. The mixture was allowed to room temperature, then diluted with EtOAc (100 mL) and filtered through celite. The filtrate was concentrated and the remaining aqueous was diluted with EtOAc (100 mL) and H_2_O (100 mL) and separated. The aqueous phase was extracted with EtOAc (2 × 75 mL). Combined organics were washed with water (100 mL) and brine (100 mL), then dried over anhydrous Na_2_SO_4_, filtered and concentrated. The crude product was purified by flash column chromatography (80% EtOAc/*n*-hexane) to give brown solid (985 mg, 4.47 mmol, 93%). R*_f_* = 0.24 (80% EtOAc/*n*-hexane); ^1^H NMR (400 MHz; CDCl_3_) δ: 8.91 (1H, d, *J* = 4.2 Hz), 8.25 (1H, s), 8.14 (1H, d, *J* = 8.1 Hz), 7.84 (1H, d, *J* = 8.5 Hz), 7.79 (1H, d, *J* = 8.6 Hz), 7.60 (2H, d, *J* = 8.5 Hz), 7.35 (1H, dd, *J* = 8.2, 4.2 Hz), 6.81 (2H, d, *J* = 8.5 Hz), 3.82 (2H, s); ^13^C NMR (101 MHz; CDCl_3_) δ: 150.9, 148.9, 146.6, 142.3, 135.8, 130.5, 128.5 (2C), 128.1, 127.0, 126.0, 125.7, 120.6, 115.6 (2C); HRMS (TOF MS ES^+^) found [M+H]^+^ 221.1070, C_15_H_13_N_2_^+^ requires 221.1079.

**4-(quinolin-7-yl)benzenesulfonyl chloride (14)**

Thionyl chloride (1 mL, 13.7 mmol) was added dropwise over 30 min to H_2_O (10 mL) at 0 °C, then allowed to room temperature overnight. Copper(I) chloride (9.8 mg, 0.12 mmol) was added to the mixture and stirred 30 min at -15 °C. Hydrochloric acid (37%, 1 mL, 33 mmol) was added dropwise to 4-(quinolin-7-yl)aniline **13** (506 mg, 2.3 mmol) at -15 °C. Sodium nitrite (190 mg, 2.8 mmol) in H_2_O (2 mL) was added dropwise to the slurry over 30 min and stirred 15 min at -15 °C to generate diazonium salt. The diazonium salt was added dropwise to a thionyl chloride solution over 30 min, then stirred, and maintained the reaction mixture at -15 °C for 1.5 h. The reaction mixture was extracted with CH_2_Cl_2_ (3 × 20 mL). Combined organics were washed with brine (20 mL), dried over anhydrous Na_2_SO_4_, filtered, and concentrated to obtain pale yellow solid (175 mg, 0.58 mmol, 25%). The crude product was used without further purification. R*_f_* = 0.58 (80% EtOAc/*n*-hexane).

***Tert*-butyl (*S*)-3-(((4-(quinolin-7-yl)phenyl)sulfonamido)methyl)pyrrolidine-1-carboxylate (15)**

To the solution of *tert*-butyl (*S*)-3-(aminomethyl)pyrrolidine-1-carboxylate (142 mg, 0.709 mmol) in CH_2_Cl_2_ (5 mL) at 0 °C, triethylamine (0.27 mL, 1.9 mmol) was added. 4-(quinolin-7-yl)benzenesulfonyl chloride **14** (175 mg, 0.576 mmol) in CH_2_Cl_2_ (15 mL) was added dropwise to the mixture and stirred at 60 °C overnight for 16 h. The reaction mixture was diluted with CH_2_Cl_2_ (50 mL) and H_2_O (50 mL) and separated. Aqueous phase was further extracted with CH_2_Cl_2_ (50 mL). Combined organics were washed with water (50 mL) and brine (50 mL), then dried over anhydrous Na_2_SO_4_, filtered and concentrated under reduced pressure. The crude product was purified by flash column chromatography (80% EtOAc/n-hexane) to give a pale yellow solid (188 mg, 0.402 mmol, 86%). R*_f_* = 0.24 (80% EtOAc/n-hexane); ^1^H NMR (400 MHz; CDCl_3_) 8.98 (1H, d, *J* = 4.3 Hz), 8.34 (1H, s), 8.22 (1H, d, *J* = 8.3 Hz), 7.98 (2H, d, *J* = 8.1 Hz), 7.94 (1H, d, *J* = 8.4 Hz), 7.89 (2H, d, *J* = 8.2 Hz), 7.81 (1H, d, *J* = 8.4 Hz), 7.46 (1H, dd, *J* = 8.4, 4.3 Hz), 5.02 – 4.94 (1H, m), 3.49 (1H, dd, J = 11.0, 7.4 Hz), 3.41 (1H, t, *J* = 10.2 Hz), 3.29 (1H, t, *J* = 8.5 Hz), 3.03 (3H, t, *J* = 6.8 Hz), 2.39 (1H, m), 2.02 – 1.91 (1H, m), 1.60 (1H, d, *J* = 7.6 Hz), 1.43 (9H, s); ^13^C NMR (101 MHz; CDCl_3_) 154.6, 151.4, 148.5, 144.9, 136.0, 132.3, 128.9, 128.6, 128.3 (2C), 128.1, 128.0, 127.9 (2C), 126.0, 121.8, 79.5, 51.6, 49.4, 45.9, 39.3, 38.4, 28.6; HRMS (TOF MS ES^+^) found [M+H]^+^ 468.1947, [C_25_H_30_N_3_O_4_S]^+^ requires 468.1957.

**(*R*)-*N*-((1-acetylpyrrolidin-3-yl)methyl)-*N*-methyl-4-(quinolin-7-yl)benzenesulfonamide (1)**

To a solution of (*R*)-*N*-methyl-*N*-(pyrrolidin-3-ylmethyl)-4-(quinolin-7-yl)benzenesulfonamide (47 mg, 0.12 mmol) in anhydrous CH_2_Cl_2_ (5 mL) under N_2_ atmosphere, pyridine (30 µL, 0.37 mmol) then acetic anhydride (20 µL, 0.21 mmol) were added dropwise. The reaction was stirred for 2 h at room temperature before quenching by pouring into saturated NaHCO_3_ (20 mL) and separated. The aqueous phase was extracted with CH_2_Cl_2_ (3 × 20 mL). The combined organics were then washed with 1 M HCl (20 mL), dried over anhydrous Na_2_SO_4_, filtered and concentrated.  Crude product was purified by flash column chromatography (5% MeOH/CH_2_Cl_2_) to give pale yellow solid (36 mg, 68%). R*_f_* = 0.28 (5% MeOH in CH_2_Cl_2_); ^1^H NMR (400 MHz, DMSO-*d*_6_) δ: 9.12 (1H, d, *J* = 4.9 Hz), 8.73 (1H, d, *J* = 8.3 Hz), 8.47 (1H, s), 8.28 (1H, d, *J* = 8.6 Hz), 8.14 (3H, t, *J* = 7.6 Hz), 7.95 (2H, d, *J* = 8.4 Hz), 7.78 (1H, dd, *J* = 8.3, 4.5 Hz), 3.58 – 3.49 (1H, m), 3.45 – 3.39 (1H, m), 3.30 – 3.15 (1H, m), 3.10 – 3.02 (1H, m), 3.02 – 2.99 (1H, m), 2.99 – 2.91 (1H, m), 2.75 (3H, d, *J* = 7.7 Hz), 2.63 – 2.51 (1H, m), 2.50 – 2.45, 2.06 – 1.95 (1H, m), 1.93 (3H, s), (d, *J* = 2.2 Hz), 1.77 – 1.54 (1H, m); ^13^C NMR (101 MHz, DMSO-*d*_6_) δ: 168.1, 149.6, 143.0, 140.8, 139.2, 136.5, 129.5 (2C), 128.3 (2C), 128.1 (2C), 127.9, 126.7, 124.3, 122.2, 52.1, 49.9, 48.5, 44.1, 36.9, 35.2, 28.9; HRMS (TOF MS ES^+^) found [M+H]^+^ 424.1700, C_23_H_26_N_3_O_3_S^+^ requires 424.1695.

**(*S*)-*N*-methyl-*N*-(pyrrolidin-3-ylmethyl)-4-(quinolin-7-yl)benzenesulfonamide•HCl (2)**

To a solution of *tert*-butyl (*S*)-3-(((*N*-methyl-4-(quinolin-7-yl)phenyl)sulfonamido)methyl)pyrrolidine-1-carboxylate (86 mg, 0.18 mmol) in CH_2_Cl_2_ (0.5 mL) under nitrogen atmosphere, 4M HCl in 1,4-dioxane (0.8 mL) was added dropwise and stirred at room temperature for 2 h. The reaction mixture was concentrated under reduced pressure and triturated in diethyl ether (50 mL) to yield the product as a brown solid precipitate (66 mg, 0.17 mmol, 97%) which was used without purification. R*_f_* = 0.42 (5% MeOH/CH_2_Cl_2_); ^1^H NMR (400 MHz; MeOD) δ: 9.29 (2H, t, *J* = 7.5 Hz), 8.52 (2H, d, *J* = 10.4 Hz), 8.37 (1H, d, *J* = 8.5 Hz), 8.15 (3H, d, *J* = 8.3 Hz), 8.05 (2H, d, *J* = 8.2 Hz), 3.52 – 3.39 (2H, m), 3.39 – 3.30 (1H, m) 3.24 – 3.17 (1H, m), 3.16 – 3.07 (2H, m), 2.85 (3H, s), 2.83 – 2.74 (1H, m), 2.27 – 2.14 (1H, m), 1.92 – 1.82 (1H, m); ^13^C NMR (101 MHz; MeOD) δ: 148.4, 147.0, 146.8, 143.9, 139.7, 139.2, 131.5, 130.7, 130.2, 130.0 (2C), 129.7 (2C), 123.4, 119.7, 53.3, 46.3, 37.8, 36.0, 28.8 (2C); HRMS (TOF MS ES^+^) found [M+H]^+^ 382.1586, [C_21_H_24_N_3_O_2_S]^+^ requires 382.1589.

***Tert*-butyl(*S*)-3-(((*N*-methyl-4-(quinolin-7-yl)phenyl)sulfonamido)methyl)pyrrolidine-1-carboxylate (3)**

To the solution of *tert*-butyl (*S*)-3-(aminomethyl)pyrrolidine-1-carboxylate (142 mg, 0.71 mmol) in CH_2_Cl_2_ (5 mL) at 0 °C, triethylamine (0.27 mL, 1.9 mmol) was added. 4-(quinolin-7-yl)benzenesulfonyl chloride **14** (175 mg, 0.58 mmol) in CH_2_Cl_2_ (15 mL) was added dropwise to the mixture and stirred at 60 °C overnight. The reaction mixture was diluted with CH_2_Cl_2_ (50 mL) and H_2_O (50 mL) and separated. Aqueous phase was further extracted with CH_2_Cl_2_ (50 mL). Combined organics were washed with water (50 mL) and brine (50 mL), then dried over anhydrous Na_2_SO_4_, filtered and concentrated under reduced pressure. The crude product was purified by flash column chromatography (80% EtOAc/*n*-hexane) to give a pale yellow solid (188 mg, 0.40 mmol, 86%). R*_f_* = 0.24 (80% EtOAc/*n*-hexane); ^1^H NMR (400 MHz; CDCl_3_) δ: 8.98 (1H, d, *J* = 4.3 Hz), 8.34 (1H, s), 8.22 (1H, d, *J* = 8.3 Hz), 7.98 (2H, d, *J* = 8.1 Hz), 7.94 (1H, d, *J* = 8.4 Hz), 7.89 (2H, d, *J* = 8.2 Hz), 7.81 (1H, d, *J* = 8.4 Hz), 7.46 (1H, dd, *J* = 8.4, 4.3 Hz), 5.02 – 4.94 (1H, m), 3.49 (1H, dd, *J* = 11.0, 7.4 Hz), 3.41 (1H, t, *J* = 10.2 Hz), 3.29 (1H, t, *J* = 8.5 Hz), 3.03 (3H, t, *J* = 6.8 Hz), 2.39 (1H, m), 2.02 – 1.91 (1H, m), 1.60 (1H, d, *J* = 7.6 Hz), 1.43 (9H, s); ^13^C NMR (101 MHz; CDCl_3_) δ: 154.6, 151.4, 148.5, 144.9, 136.0, 132.3, 128.9, 128.6, 128.3 (2C), 128.1, 128.0, 127.9 (2C), 126.0, 121.8, 79.5, 51.6, 49.4, 45.9, 39.3, 38.4, 28.6; HRMS (TOF MS ES^+^) found [M+H]^+^ 468.1947, [C_25_H_30_N_3_O_4_S]^+^ requires 468.1957.

**AZ’902 (*S*)-*N*-((1-acetylpyrrolidin-3-yl)methyl)-*N*-methyl-4-(quinolin-7-yl)benzenesulfonamide**

To a solution of (*S*)-*N*-methyl-*N*-(pyrrolidin-3-ylmethyl)-4-(quinolin-7-yl)benzene sulfonamide (47 mg, 0.12 mmol) in anhydrous CH_2_Cl_2_ (5 mL) under nitrogen atmosphere, pyridine (30 µL, 0.37 mmol) then acetic anhydride (20 µL, 0.21 mmol) were added dropwise. The reaction was stirred for 2 h at room temperature before quenching by pouring into saturated NaHCO_3_ (20 mL) and separated. The aqueous phase was extracted with CH_2_Cl_2_ (3 × 20 mL). The combined organics were then washed with 1 M HCl (20 mL), dried over anhydrous Na_2_SO_4_, filtered and concentrated under reduced pressure. Crude product was purified by flash column chromatography (5% MeOH/CH_2_Cl_2_) to give product as a pale yellow solid (36 mg, 0.084 mmol, 68%). R*_f_* = 0.28 (5% MeOH/ in CH_2_Cl_2_); ^1^H NMR (400 MHz; DMSO-*d*_6_) 8.98 (1H, dd, *J* = 4.3, 1.7 Hz), 8.44 (1H, d, *J* = 8.5 Hz), 8.38 (1H, s), 8.14 (3H, dd, *J* = 8.0, 3.6 Hz), 8.03 (1H, dd, *J* = 8.5, 1.9 Hz), 7.92 (2H, d, *J*=8.1 Hz), 7.59 (1H, dd, *J* = 8.3, 4.2 Hz), 3.58 – 3.47 (1H, m), 3.47 – 3.37 (1H, m), 3.30 – 3.15 (1H, m), 3.10 – 3.03 (1H, m), 3.04 – 2.97 (1H, m), 2.94 (1H, dd, *J* = 13.3, 7.0 Hz), 2.75 (3H, d, *J* = 7.7 Hz), 2.58 (1H, d, *J* = 7.1 Hz), 2.04 – 1.97 (1H, m), 1.93 (3H, d, *J* = 2.3 Hz), 1.79 – 1.55 (1H, m); ^13^C NMR (101 MHz; DMSO-*d*_6_) 168.1, 149.6, 143.0, 140.8, 139.2, 136.5, 129.5 (2C), 128.3 (2C), 128.1 (2C), 127.9, 126.7, 124.3, 122.2, 52.1, 49.9, 48.5, 44.1, 36.9, 35.2, 28.9; HRMS (TOF MS ES^+^) found [M+H]^+^ 424.1700, [C_23_H_26_N_3_O_3_S]^+^ requires 424.1695.

**Synthesis of 7PQ analogues**


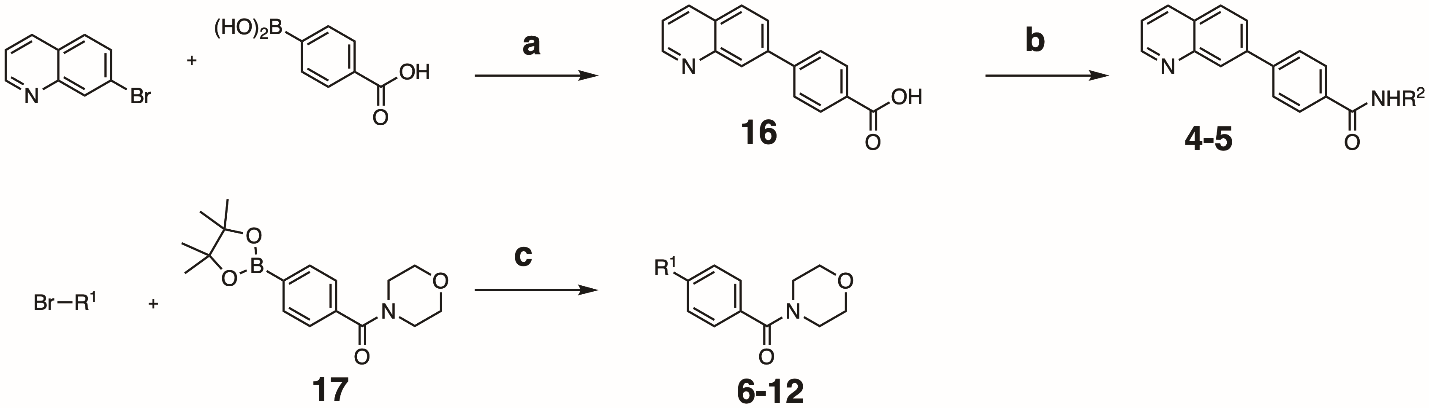


Reagents and conditions: (**a**) Pd(PPh_3_)_4_, Na_2_CO_3_, H_2_O/MeCN, 90 °C, 18 h, 78%; (**b**) R^2^-NH_2_, DCC, DMAP, CH_2_Cl_2_, rt, 18 h.; (**c**) R^1^-Br, Pd(PPh_3_)_4_, Na_2_CO_3_, H_2_O/MeCN, 130 °C, 18 h

**4-(quinolin-7-yl)benzoic acid (16)**

7-bromoquinoline (803 mg, 3.86 mmol) and 4-phenylbenzoic acid (637 mg, 3.8 mmol) were dissolved in MeCN (20 mL) and Na_2_CO_3_ (212 mg, 0.5 mmol) in H_2_O (20 mL) was added. Pd(PPh_3_)_4_ (224 mg, 0.19 mmol) was added and the reaction mixture was stirred at 90 °C overnight. The mixture was allowed to room temperature and filtered through celite. The filtrate was concentrated and the remaining aqueous was washed with CH_2_Cl_2_, acidified to pH 1 with 2 M HCl. Precipitation was collected by filtration and washed with water (2 × 20 mL) and Et_2_O (2 × 20 mL). White solid was obtained as a product (747 mg, 78%). R*_f_* = 0.10 (80% EtOAc/*n*-hexane); ^1^H NMR (400 MHz, DMSO-*d*_6_) δ: 13.08 (1H, s), 9.06 (1H, dd, *J* = 4.5 Hz), 8.63 (1H, d, *J* = 9.1 Hz), 8.41 (1H, s), 8.22 (1H, d, *J* = 8.6 Hz), 8.10 (3H, d, *J* = 8.1 Hz), 8.00 (2H, d, *J* = 8.3 Hz), 7.71 (1H, dd, *J* = 8.3, 4.5 Hz); ^13^C NMR (101 MHz, DMSO-*d*_6_) δ: 167.0, 150.0, 145.6, 143.1, 141.0, 138.3, 130.4, 130.2 (2C), 129.3, 127.7, 127.5 (2C), 126.4, 124.7, 122.0; HRMS (TOF MS ES^+^) found [M+H]^+^ 250.0873, C_16_H_12_NO_2_^+^ requires 250.0868.

**General Procedure b**

To a solution of 4-(quinolin-7-yl)benzoic acid **16** (0.40 mmol), DMAP (0.80 mmol) and DCC (0.60 mmol) in CH_2_Cl_2_ (10 mL), amine (0.48 mmol) was added and stirred at room temperature overnight. The mixture was filtered, and the filtrate was washed with water (2 × 20 mL) and brine (2 × 20 mL), then dried over anhydrous Na_2_SO_4_, filtered and concentrated. Crude product was purified by flash column chromatography.

**General Procedure c**

To a solution of aryl bromide (0.32 mmol) and 4-(morpholine-4-carbonyl)phenylboronic acid pinacol ester **17** (0.32 mmol) in MeCN (5 mL) was added a solution of Na_2_CO_3_ (0.16 mmol) in H_2_O (5 mL). Pd(PPh_3_)_4_ (0.01 mmol) was added and the reaction mixture was stirred at 130 °C overnight. The reaction mixture was cooled to room temperature, filtered through the Celite® and washed the Celite® with EtOAc (2 × 20 mL). The filtrate was concentrated under reduced pressure and the remaining aqueous was diluted with EtOAc (20 mL) and H_2_O (20 mL) and separated. The aqueous phase was extracted with EtOAc (2 × 20 mL). Combined organics were washed with water (20 mL) and brine (20 mL), then dried over anhydrous Na_2_SO_4_, filtered and concentrated under reduced pressure. Crude product was purified by flash column chromatography.

***Tert*-butyl (*R*)-3-((4-(quinolin-7-yl)benzamido)methyl)pyrrolidine-1-carboxylate (4)**

Prepared according to **General Procedure b**, the crude product was purified on silica gel (80% EtOAc/*n*-hexane) product was a white solid (78 mg, 0.18 mmol, 45%).; R*_f_* = 0.17 (90% EtOAc/*n*-hexane); ^1^H NMR (400 MHz; DMSO-*d*_6_) δ: 8.96 (1H, dd, *J* = 4.3, 1.7 Hz), 8.70 (1H, br), 8.42 (1H, d, *J* = 8.5 Hz), 8.35 (1H, s), 8.11 (1H, d, *J* = 8.5 Hz), 8.03 (1H, d, *J* =2.1 Hz), 8.00 (4H, d, *J* = 3.9 Hz), 7.56 (1H, dd, *J* = 8.3, 4.2 Hz), 3.44 – 3.35 (2H, m), 3.33 – 3.15 (2H, m), 3.02 (1H, m), 2.45 (1H, d, *J* = 6.2 Hz), 1.92 (1H, d, *J* = 6.3 Hz), 1.74 – 1.56 (2H, m), 1.39 (9H, s); ^13^C NMR (101 MHz, DMSO-*d*_6_) δ: 166.0, 153.6, 151.2, 148.0, 141.8, 140.1, 135.8, 133.8, 128.9, 128.1 (2C), 127.4, 127.1 (2C), 126.5, 125.7, 121.7, 78.2, 49.3, 49.1, 45.1, 44.9, 41.5, 28.2 (3C); HRMS (TOF MS ES^+^) found [M+H]^+^ 432.2280, [C_26_H_30_N_3_O_3_]^+^ requires 432.2287.

***Tert*-butyl (*R*)-3-(4-(quinolin-7-yl)benzamido)pyrrolidine-1-carboxylate (5)**

Prepared according to **General Procedure b**, the crude product was purified on silica gel (80% EtOAc/*n*-hexane) product was a pale yellow solid (52 mg, 0.12 mmol, 31%); R*_f_* = 0.27 (90% EtOAc/*n*-hexane); ^1^H NMR 400 MHz, DMSO-*d*_6_) δ: 8.95 (1 H, s), 8.66 (1 H, d, *J* = 6.5 Hz), 8.41 (1 H, d, *J* = 7.6 Hz), 8.35 (1 H, s), 8.10 (1 H, d, *J* = 8.1 Hz), 8.06 – 7.94 (5 H, m), 7.59 – 7.52 (1 H, m), 4.53 – 4.35 (1 H, m), 3.65 – 3.47 (2 H, m), 3.31 – 3.18 (2 H, m), 2.22 – 2.05 (1 H, m), 2.01 – 1.85 (1 H, m), 1.41 (3 H, s); ^13^C NMR (101 MHz, DMSO-*d*_6_) δ: 166.1, 153.5, 151.2, 148.0, 141.9, 140.1, 135.8, 133.6, 128.9, 128.3 (2C), 127.4, 127.0 (2C), 126.5, 125.7, 121.7, 78.3, 50.8, 49.6, 48.9, 43.9, 28.2 (3C); HRMS (TOF MS ES^+^) found [M+H]^+^ 418.2121, C_25_H_28_N_3_O_3_^+^ requires 418.2131.

**Morpholino(4-(quinolin-7-yl)phenyl)methanone (6)**

Prepared according to **General Procedure c** using 7-bromoquinoline, the crude product was purified on silica gel (5%MeOH/CH_2_Cl_2_) product was a white solid (61 mg, 0.18 mmol, 90%).; R*_f_* = 0.61 (70% EtOAc/*n*-hexane); ^1^H NMR (400 MHz; CDCl_3_) δ: 8.97 – 8.96 (1H, d, *J* = 8.0 Hz), 8.34 (1H, s), 8.21 – 8.19 (1H, d, *J* = 8.0 Hz), 7.93 – 7.79 (3H, m), 7.57 – 7.54 (2H, m), 7.45 – 7.42 (1H, m), 3.99 – 3.61 (8H, m); ^13^C NMR (101 MHz, CDCl_3_) δ: 151.1, 147.6, 146.9, 142.8, 134.5, 128.5, 128.0, 127.7, 127.5, 126.0, 124.0, 123.7, 121.9, 67.6, 61.7; HRMS (TOF MS ES^+^) found [M+H]^+^ 319.1448, [C_20_H_19_N_2_O_2_]^+^ requires 319.1447.

**(4-(isoquinolin-7-yl)phenyl)(morpholino)methanone (7)**

Prepared according to **General Procedure c**, using 7-bromoisoquinoline, the crude product was purified on silica gel (70% EtOAc/*n*-hexane) product was an off white solid (73 mg, 0.23 mmol, 72%).; R*_f_* = 0.11 (80% EtOAc/*n*-hexane); ^1^H NMR (400 MHz; DMSO-*d*_6_) δ: 9.42 (1H, s), 8.54 (1H, s), 8.49 (1H, s), 8.17 (1H, d, *J* = 8.6 Hz), 8.09 (1H, d, *J* = 8.6 Hz), 7.93 (2H, d, *J* = 8.0 Hz), 7.89 (1H, d, *J* = 5.6 Hz), 7.58 (2H, d, *J* = 7.9 Hz), 3.63 (8H, br); ^13^C NMR (101 MHz, DMSO-*d*_6_) δ: 168.8, 152.8, 143.0, 140.4, 138.1, 135.0, 134.6, 129.6, 128.0 (2C), 127.9, 127.4, 127.1 (2C), 125.4, 120.2, 66.1 (4C); HRMS (TOF MS ES^+^) found [M+H]^+^ 319.1443, [C_20_H_19_N_2_O_2_]^+^ requires 319.1447.

**(4-(isoquinolin-6-yl)phenyl)(morpholino)methanone (8)**

Prepared according to **General Procedure c**, using 6-bromoisoquinoline, the crude product was purified on silica gel (70% EtOAc/*n*-hexane) product was an off white solid (73 mg, 0.23 mmol, 73%).; R*_f_* = 0.11 (80% EtOAc/*n*-hexane); ^1^H NMR (400 MHz; DMSO-*d*_6_) δ: 9.36 (1H, s), 8.54 (1H, s), 8.32 (1H, s), 8.23 (1H, d, *J* = 8.6 Hz), 8.05 (1H, d, *J* = 8.6 Hz), 7.92 (3H, t, *J* = 8.6 Hz), 7.58 (2H, d, *J* = 7.8 Hz), 3.63 (8H, br); ^13^C NMR (101 MHz, DMSO-*d*_6_) δ: 168.7, 152.1, 143.3, 141.1, 140.3, 135.6, 135.3, 128.5, 128.0 (2C), 127.4 (2C), 126.9, 126.6, 124.2, 120.8, 66.1 (4C); HRMS (TOF MS ES^+^) found [M+H]^+^ 319.1443, [C_20_H_19_N_2_O_2_]^+^ requires 319.1447.

**Morpholino(4-(quinolin-6-yl)phenyl)methanone (9)**

Prepared according to **General Procedure c**, using 6-bromoquinoline, the crude product was purified on silica gel (70% EtOAc/*n*-hexane) product was an off white solid (73 mg, 0.23 mmol, 73%).; R*_f_* = 0.16 (80% EtOAc/*n*-hexane); ^1^H NMR (400 MHz, DMSO-*d*_6_) δ: 8.92 (1H, d, *J* = 2.6 Hz), 8.45 (1H, d, *J* = 8.0 Hz), 8.35 (1H, s), 8.12 (2H, s), 7.92 (2H, d, *J* = 8.0 Hz), 7.61 – 7.55 (3H, m), 3.63 (8H, br,); ^13^C NMR (101 MHz; DMSO-*d*_6_) δ: 168.8, 150.9, 147.3, 140.4, 137.1, 136.5, 134.9, 129.6, 128.5, 128.2, 127.9 (2C), 127.1 (2C), 125.9, 122.0, 66.1 (4C); HRMS (TOF MS ES^+^) found [M+H]^+^ 319.1433, [C_20_H_19_N_2_O_2_]^+^ requires 319.1447.

**Morpholino(4-(quinolin-3-yl)phenyl)methanone (10)**

Prepared according to **General Procedure c**, using 3-bromoquinoline, the crude product was purified on silica gel (70% EtOAc/*n*-hexane) product was an off white solid (82 mg, 0.26 mmol, 82%).; R*_f_* = 0.22 (80% EtOAc/*n*-hexane); ^1^H NMR (400 MHz, DMSO-*d*_6_) δ: 9.29 (1H, s), 8.72 (1H, s), 8.07 (2H, d, *J* = 8.2 Hz), 7.98 (2H, d, *J* = 7.9 Hz), 7.80 (1H, t, *J* = 7.7 Hz), 7.67 (1H, t, *J* = 7.6 Hz), 7.60 (2H, d, *J* = 7.8 Hz), 3.64 (8H, br); ^13^C NMR (101 MHz, DMSO-*d*_6_) δ: 168.7, 149.3, 147.0, 138.2, 135.2, 133.3, 132.0, 129.8, 128.7, 128.5, 128.0 (2C), 127.6, 127.2 (2C), 127.1, 66.1 (4C); HRMS (TOF MS ES^+^) found [M+H]^+^ 319.1439, [C_20_H_19_N_2_O_2_]^+^ requires 319.1447.

**Morpholino(4-(quinolin-2-yl)phenyl)methanone (11)**

Prepared according to **General Procedure c**, using 2-bromoquinoline, the crude product was purified on silica gel (70% EtOAc/*n*-hexane) product was a white solid (86 mg, 0.27 mmol, 85%).; R*_f_* = 0.28 (80% EtOAc/*n*-hexane); ^1^H NMR (400 MHz; DMSO-*d*_6_) δ: 8.49 (1H, d, *J* = 8.6 Hz), 8.35 (2H, d, *J* = 7.8 Hz), 8.20 (1H, d, *J* = 8.7 Hz), 8.09 (1H, d, *J* = 8.5 Hz), 8.02 (1H, d, *J* = 8.1 Hz), 7.80 (1H, t, *J* = 7.7 Hz), 7.65 – 7.56 (3H, m), 3.85 – 3.37 (8H, m); ^13^C NMR (101 MHz, DMSO-*d*_6_) δ: 168.8, 155.3, 147.5, 139.6, 137.4, 136.5, 130.1, 129.2, 128.8, 127.9, 127.7, 127.3, 127.1, 126.7, 118.8, 114.9, 66.1 (4C); HRMS (TOF MS ES^+^) found [M+H]^+^ 319.1440, [C_20_H_19_N_2_O_2_]^+^ requires 319.1447.

**Morpholino(4-(quinoxalin-6-yl)phenyl)methanone (12)**

Prepared according to **General Procedure c**, using 6-bromoquinoxaline, the crude product was purified on silica gel (80% EtOAc/*n*-hexane) product was a white solid (65 mg, 0.20 mmol, 70%).; R*_f_* = 0.19 (100% EtOAc); ^1^H NMR (400 MHz, DMSO-*d*_6_) δ: 8.99 (2H, d, *J* = 12.7 Hz), 8.40 (1H, s), 8.25 (1H, dd, *J* = 8.7, 2.1 Hz), 8.20 (1H, d, *J* = 8.7 Hz), 7.98 (2H, d, *J* = 8.0 Hz), 7.59 (2H, d, *J* = 8.0 Hz), 3.64 (8H, br); ^13^C NMR (101 MHz, DMSO-*d*_6_) δ: 168.7, 146.4, 145.9, 142.5, 141.8, 140.9, 139.7, 135.4, 129.9, 129.4, 128.0 (2C), 127.5 (2C), 126.5, 66.1 (4C); HRMS (TOF MS ES^+^) found [M+H]^+^ 320.1405, [C_19_H_18_N_3_O_2_]^+^ requires 320.1399.

**Synthesis of the affinity-based probe 7PQYnD**


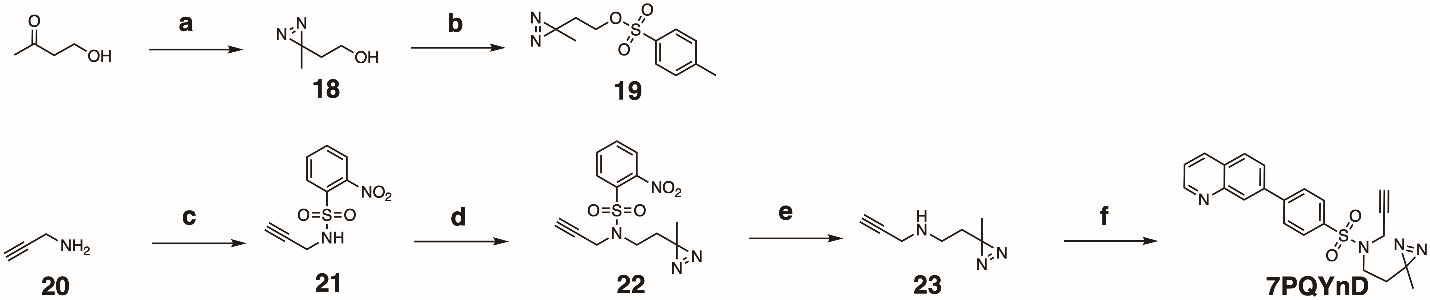


 Reagents and conditions: (**a**) (i) NH_3_, MeOH, 5 h, -78 °C, (ii) hydroxylamine-*O*-sulfonic acid (HOSA), 16 h, -78 °C to rt, (iii) I_2_, DIPEA, MeOH, 1 h, 0 °C, 36%; (**b**) *p*-toluenesulfonyl chloride (*p*-TsCl), pyridine, 3 h, 0 °C to rt, 65%; (**c**) 2-nitrobenzenesulfonyl chloride, NEt_3_, CH_2_Cl_2_, 3 h, 0 °C to rt, N_2_, 88%; (**d**) **21**, K_2_CO_3_, DMF, 5 h, 80 °C, 78%; (**e**) LiOH^.^H_2_O, 3-mercaptopropionic acid (HS(CH_2_)_2_COOH), DMF, 5 h, rt, 75%; (**f**) **14**, NEt_3_, CH_2_Cl_2_, 0 °C to rt, 2.5 h, 48%.

**2-(3-methyl-3H-diazirin-3-yl)ethan-1-ol (18)**

Ammonia (50 mL) was condensed at –78 °C into a 250 mL round bottom flask fitted with a cold finger of dry ice-acetone and an HCl (6 M) trap. A solution of 4-hydroxybutan-2-one (95%, 6.52 g, 70.3 mmol, 1 eq.) in anhydrous MeOH (10 mL) was added and the reaction stirred at -78 °C for 5 h. A solution of hydroxylamine-*O*-sulphonic acid (HOSA, 10.9 g, 96.5 mmol, 1.4 eq.) in anhydrous MeOH (70 mL) was added dropwise at –78 °C and the reaction stirred and allowed to warm to rt for 16 h. The reaction mixture was then filtered, and the solids washed with anhydrous MeOH (2 × 20 mL). The filtrate was concentrated *in vacuo* (water bath 30 °C) and the residue re-dissolved in anhydrous MeOH (50 mL), cooled to 0 °C, and *N,N*-diisopropylethylamine (DIPEA, 15.0 mL, 87.2 mmol, 1.2 eq.) was added. I_2_ was added portion-wise to the stirred solution until a dark brown color persisted for 1 h. The reaction mixture was diluted with Et_2_O (150 mL) and HCl (1 M, 150 mL) and separated. The aqueous layer was extracted with Et_2_O (2 × 100 mL) and the combined organics washed successively with HCl (1 M, 150 mL), Na_2_S_2_O_3_ (10% (w/v) aq., 150 mL) and brine (150 mL), then dried over anhydrous MgSO_4_, filtered and concentrated *in vacuo* to afford product as a pale yellow oil (2.45 g, 24.5 mmol, 36%) that was used without further purification: R*_f_* = 0.53 (10% MeOH/CH_2_Cl_2_); ^1^H NMR (400 MHz, CDCl_3_) 3.52 (2H, t, *J* = 6.3 Hz), 1.62 (2H, t, *J* = 6.3 Hz), 1.06 (3H, s); HRMS (ESI^+^) found [M+H]^+^ 101.0720, [C_4_H_9_N_2_O]^+^ requires 101.0715.

**2-(3-methyl-3H-diazirin-3-yl)ethyl 4-methylbenzenesulfonate (19)**

2-(3-methyl-3*H*-diazirin-3-yl)ethan-1-ol **18** (1.00 g, 10.0 mmol, 1 eq.) was dissolved in anhydrous pyridine (8 mL) and cooled to 0 °C. *p-*toluenesulfonyl chloride (2.87 g, 15.0 mmol, 1.5 eq.) was added portion-wise and the resulting solution stirred for 3 h at rt. The reaction mixture was diluted with CH_2_Cl_2_ (150 mL) and HCl (1 M, 150 mL), separated, and the organic phase washed successively with HCl (1 M, 75 mL), sat. aq. NaHCO_3_ (150 mL) and brine (150 mL), then dried over anhydrous MgSO_4_, filtered and concentrated *in vacuo*. The crude product was purified by automated flash column chromatography (8–66% EtOAc in *n*-hexane) to afford product as a colorless oil (1.65 g, 6.50 mmol, 65%): R*_f_* = 0.57 (CH_2_Cl_2_); ^1^H NMR (400 MHz, CDCl_3_) 7.85 – 7.77 (2H, m), 7.36 (2H, dd, *J* = 8.6, 0.7 Hz), 3.95 (2H, t, *J* = 6.4 Hz), 2.45 (3H, s), 1.67 (2H, t, *J* = 6.4 Hz), 1.00 (3H, s); HRMS (MS TOF ESI^+^) found [M+H]^+^ 255.0814, [C_11_H_15_N_2_O_3_S]^+^ requires 255.0803.

**2-nitro-*N-*(prop-2-yn-1-yl)benzenesulfonamide (21)**

Propargylamine **20** (1.00 g, 18.2 mmol, 1.06 eq.) and triethylamine (2.52 mL, 18.2 mmol, 1.06 eq.) were dissolved in CH_2_Cl_2_ (15 mL) and cooled to 0 °C under nitrogen atmosphere. 2-Nitrobenzenesulfonyl chloride (3.80 g, 17.1 mmol, 1 eq.) was added and the reaction stirred at rt for 3 h. The reaction mixture was diluted with CH_2_Cl_2_ (50 mL) and HCl (2 M, 50 mL) and separated. The aqueous layer was further extracted with CH_2_Cl_2_ (50 mL) and the combined organics were washed successively with HCl (2 M, 75 mL), H_2_O (75 mL), and brine (75 mL), then dried over anhydrous Na_2_SO_4_, filtered and concentrated *in vacuo* to afford product as an pale orange solid (3.64 g, 15.0 mmol, 88%) which was used without further purification: R*_f_* = 0.46 (50% EtOAc/n-hexane); ^1^H NMR (400 MHz, CDCl_3_) 8.23 – 8.16 (1H, m), 7.95 – 7.88 (1H, m), 7.80 – 7.73 (2H, m), 5.71 (1H, t, *J* = 6.4 Hz), 4.01 (2H, dd, *J* = 6.3, 2.5 Hz), 1.97 (1H t, *J* = 2.5 Hz); HRMS (TOF MS ESI^-^) found [M-H]^-^ 239.0130, [C9H7N2O4S]^-^ requires 239.0127.

***N*-(2-(3-methyl-3H-diazirin-3-yl)ethyl)-2-nitro-*N*-(prop-2-yn-1- yl)benzenesulfonamide (22)**

K_2_CO_3_ (2.69 g, 19.5 mmol, 3.33 eq.) and 2-nitro-*N*-(prop-2-yn-1-yl)benzenesulfonamide **21** (1.56 g, 6.50 mmol, 1 eq.) were added sequentially to a stirred solution of 2-(3-methyl-3H-diazirin-3-yl)ethyl 4-methylbenzenesulfonate 14 (1.65 g, 6.50 mmol, 1 eq.) in dimethylformamide (DMF, 13 mL) and the reaction stirred at 80 °C for 3 h. The reaction mixture was diluted with EtOAc (75 mL) and H_2_O (75 mL) and separated. The aqueous phase was further extracted with EtOAc (2 × 50 mL) and the combined organics were washed successively with LiCl (5% (w/v) aq., 2 × 100 mL) and brine (100 mL), then dried over anhydrous MgSO_4_, filtered and concentrated *in vacuo*. The crude product was purified by automated flash column chromatography (20–100% EtOAc in n-hexane) to afford product as a pale yellow oil (1.64 g, 5.09 mmol, 78%): R*_f_* = 0.22 (33% EtOAc/n-hexane); ^1^H NMR (400 MHz, CDCl_3_) 8.08 – 7.98 (1H, m), 7.79 – 7.65 (2H, m), 7.65 – 7.59 (1H, m), 4.17 (2H, d, *J* = 2.5 Hz), 3.46 – 3.37 (2H, m), 2.18 (1H t, *J* = 2.5 Hz), 1.66 – 1.56 (2H, m), 1.05 (3H, s); HRMS (TOF MS ESI^+^) found [M+H]^+^ 323.0815, [C_13_H_15_N_4_O_4_S]^+^ requires 323.0814.

***N*-(2-(3-methyl-3H-diazirin-3-yl)ethyl)prop-2-yn-1-amine (23)**

Lithium hydroxide monohydrate (857 mg, 20.4 mmol, 4 eq.) and 3-mercaptopropanoic acid (0.900 mL, 10.3 mmol, 2 eq.) were added to a stirred solution of *N*-(2-(3-methyl-3*H*-diazirin-3-yl)ethyl)-2-nitro-*N*- (prop-2-yn-1-yl)benzenesulfonamide **22** (1.64 g, 5.09 mmol, 1 eq.) in DMF (12.5 mL) and the reaction stirred at rt for 5 h. The reaction mixture was diluted with EtOAc (100 mL) and H_2_O (100 mL) and separated. The aqueous phase was further extracted with EtOAc (2 × 100 mL) and the combined organics were washed successively with LiCl (5% (w/v) aq., 200 mL), sat. aq. NaHCO_3_ (200 mL) and brine (100 mL), then dried over anhydrous MgSO_4_, filtered and concentrated *in vacuo*. The crude product was purified by flash column chromatography (50% EtOAc/*n*-hexane) to afford product as a yellow oil (520 mg, 3.80 mmol, 75%): R*_f_* = 0.22 (50% EtOAc/*n*-hexane); ^1^H NMR (400 MHz, CDCl_3_) 3.37 (2H, d, *J* = 2.4 Hz), 2.54 (2H, t, *J* = 7.1 Hz), 2.20 (1H, t, *J* = 2.4 Hz), 1.55 (2H, t, *J* = 7.1 Hz), 1.31 (1H, s), 1.02 (3H, s); HRMS (TOF MS ESI^+^) found [M+H]^+^ 138.1025, [C_7_H_12_N_3_]^+^ requires 138.1031.

***N*-(2-(3-methyl-3H-diazirin-3-yl)ethyl)-*N*-(prop-2-yn-1-yl)-4-(quinolin-7-yl)benzenesulfonamide (7PQYnD)**

Triethylamine (0.18 mL, 1.30 mmol, 2.1 eq.) was added to a stirred solution of *N*-(2-(3-methyl-3*H*- diazirin-3-yl)ethyl)prop-2-yn-1-amine 19 (100 mg, 0.730 mmol, 1.2 eq.) in CH_2_Cl_2_ (2 mL) and the solution cooled to 0 °C. Crude 4-(quinolin-7-yl)benzenesulfonyl chloride **14** (185 mg, 0.609 mmol, 1 eq.) was added and the reaction stirred at rt for 2.5 h. The reaction mixture was diluted with CH_2_Cl_2_ (20 mL) and H_2_O (20 mL) and separated. The aqueous phase was further extracted with CH_2_Cl_2_ (20 mL) and the combined organics were washed successively with H_2_O (20 mL) and brine (20 mL), then dried over anhydrous MgSO_4_, filtered and concentrated *in vacuo*. The crude product was purified by automated flash column chromatography (10–100% EtOAc in *n*-hexane) to afford product as an off-white solid (118 mg, 0.292 mmol, 48%). ^1^H NMR (400 MHz, CDCl_3_) 8.98 (1H, d, *J* = 3.4 Hz), 8.40 – 8.34 (1H, m), 8.27 – 8.19 (1H, m), 8.00 – 7.96 (2H, m), 7.95 (1H d, *J* = 8.1 Hz), 7.92 – 7.87 (2H, m), 7.83 (1H, dd, *J* = 8.5, 1.8 Hz), 7.47 (1H, dd, *J* = 8.3, 4.2 Hz), 4.18 (2H, d, *J* = 2.5 Hz), 3.32 – 3.22 (2H, m), 2.05 (1H t, *J* = 2.5 Hz), 1.69 – 1.60 (2H, m), 1.09 (3H, s); ^13^C NMR (101 MHz, CDCl_3_) 151.2, 148.3, 145.0, 140.4, 137.7, 138.4, 136.2, 128.9, 128.6 (2C), 128.1 (2C), 127.9, 126.0, 121.8, 76.2, 74.5, 42.0, 36.8, 33.5, 24.2, 19.6; HRMS (TOF MS ESI^+^) found [M+H]^+^ 405.1381, [C_22_H_21_N_4_O_2_S]^+^ requires 405.1385.

**Synthesis of AZ'320**


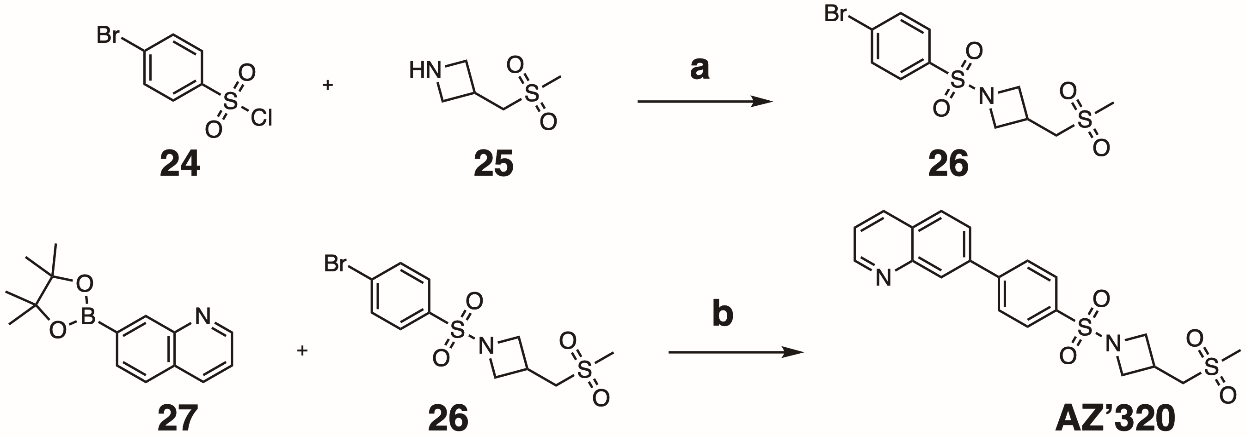


Synthetical procedure to prepare compound **AZ'320** Reagents and conditions: (**a**) TEA, N_2_, r.t., 16 h, 93%; (**b**) PdCl_2_(PPh_3_)_2_, XPhos, K_2_CO_3_, TBAB, N_2_, 95 ºC, overnight, 71%.

**1-((4-bromophenyl)sulfonyl)-3-((methylsulfonyl)methyl)azetidine (26)**

A solution of 3-((methylsulfonyl)methyl)azetidine **25** (104 mg, 0.407 mmol, 1 eq.), 4-bromobenzene-1-sulfonyl chloride **24** (100 mg, 46.4% m/m, 0.310 mmol, 0.76 eq.), and triethylamine (0.17 mL, 1.221 mmol, 3 eq.) in DCM (5 mL) was stirred at room temperature for 16 h. After completion, H_2_O (20 mL) was added, and the mixture was extracted with EtOAc (3 x 10 mL). The organic layer was dried over Na_2_SO_4_, filtered and the solvent removed under reduced pressure to obtain the product as a white solid (106 mg, 0.289 mmol, 93%). ^1^H NMR (300 MHz, CDCl_3_) δ(ppm): 7.73 (2H, d, *J* = 8.9 Hz), 7.68 (2H, d, *J* = 8.8 Hz), 3.99 (2H, dd, *J* = 8.4, 8.4 Hz), 3.70 (2H, dd, *J* = 8.4, 5.9 Hz), 3.19 (2H, d, *J* = 7.5 Hz), 3.14 – 2.94 (1H, m), 2.88 (3H, s).

**7-(4-((3-((methylsulfonyl)methyl)azetidin-1-yl)sulfonyl)phenyl)quinoline (AZ’320)**

A solution of the 1-((4-bromophenyl)sulfonyl)-3-((methylsulfonyl)methyl)azetidine **26** (52 mg, 0.204 mmol, 1.5 eq.), 7-quinolinyl boronic acid pinacol ester **27** (50 mg, 0.136 mmol, 1 eq.), PdCl_2_(PPh_3_)_2_ (9.5 mg, 13.6 µmol, 0.1 eq.) and XPhos (6.5 mg, 13.6 µmol, 0.1 eq.) in dioxane (1.75 mL) was stirred for 0.5 h at 95ºC. A tetrabutylammonium bromide solution (1.36 mL, 0.136 mmol, 1 eq.) was added and the mixture was stirred for 5 min, followed by the addition of a K_2_CO_3_ solution (0.170 mL, 0.679 mmol, 5 eq.). The reaction mixture was stirred overnight at 95 ºC. After completion, the mixture was diluted in H_2_O (20 mL) and extracted with EtOAc (4 x 20 mL). The catalyst was removed by vacuum filtration through a pad of celite. The filtrate was dried with anhydrous Na_2_SO_4_, and the solvent removed under reduced pressure. The crude product was purified by flash chromatography on silica gel to obtain the product as a white solid (40 mg, 96.6 µmol, 71%). ^1^H NMR (300 MHz, DMSO-*d*_6_) δ: 8.97 (1H, dd, *J* = 4.2, 1.8 Hz), 8.42 (1H, dd, *J* = 7.5, 1.7 Hz), 8.41 (1H, s), 8.19 (2H, d, *J* = 8.3 Hz), 8.13 (1H, d, *J* = 8.6 Hz), 8.03 (1H, dd, *J* = 8.5, 1.8 Hz), 7.95 (1H, d, *J* = 8.4 Hz), 7.57 (1H, dd, *J* = 8.3, 4.2 Hz), 4.00 – 3.87 (2H, m), 3.63 (2H, dd, *J* = 8.5, 6.4 Hz), 3.27 (2H, d, *J* = 7.5 Hz), 3.02 – 2.93 (1H, m), 2.91 (3H, s). ^13^C NMR (75 MHz, DMSO-*d*_6_) δ: 151.4, 147.9, 144.1, 139.2, 135.8, 133.1, 129.1, 128.9, 128.2, 127.7, 127.1, 125.6, 122.0, 56.0, 55.2, 40.4, 22.8. HRMS (TOF MS ESI^+^) found [M+H]^+^ 417.0937, [C_20_H_20_N_2_O_4_S]^+^ requires 417.0939. HPLC: Retention time 2.37 minutes, 100%.

**Kinase panel screen**

The kinase data was provided by Thermofisher SelectScreen Biochemical Kinase Profiling service using the Z-lyte technology. Each compound was profiled at 1mM against a representative panel of 25 kinases.

**Click reaction with Azide-TAMRA**

I2.1 cells were seeded in sterile 6-well plates at a density of 2×10^6^ cells per well and incubated at 37 °C overnight. 1000× stocks of the desired concentration of compound in DMSO were pre-prepared. Cells were treated with either the parent compound or DMSO with a final concentration of 0.1% DMSO and incubated for 1 h before treating with probe and further incubating for 3 h. TNF (final concentration 575 pM) was added to induce necroptosis and the cells incubated for 1 h before irradiation with UV light (365 nm) at various time points or kept in the dark before being harvested.

Cells were transferred to a microcentrifuge tube, centrifuged at 200 x g for 5 min and media removed. Cells were washed with PBS and lysed with 100 µL of lysis buffer (as detailed before) on ice for 30 min. The lysate was collected by centrifugation at 17,000 x g, 4 °C for 5 min and the lysate transferred to a new microcentrifuge tube. Protein concentration was determined using the DC Protein Assay (Bio-Rad) in a 96 well plate as per the manufacturer’s instructions and the concentration adjusted to 1-2 mg/mL using lysis buffer.

The “Click mixture” was prepared by combining a final concentration of 100 μM AzTAMRA, 1 mM CuSO_4_, 1 mM TCEP and 100 μM TBTA, and incubated for 2 min at rt. 6 μL of the click mixture was added to every 100 μL of lysate. The reaction mixtures were shaken at rt for 1 h before being quenched with EDTA (500 mM EDTA in H_2_O, to a final concentration of 5 mM). Proteins were precipitated in ACN (4 vol) and briefly vortexed. The mixture was centrifuged at 10,000 x g, 4 °C for 5 min and the supernatant was gently removed. The pellet was washed with 80% (v/v) EtOH (10 vol), centrifuged at 16,000 x g, 4 °C for 2 min, the EtOH removed, and the process repeated for a total of three washes. The pellet was resuspended in 1% SDS in PBS, prior to diluting with PBS to a final concentration of 1-2 mg/mL of protein in 0.2% SDS.

Samples were prepared by adding 4 μL of 4× loading buffer to 10 µL of the protein samples and boiling at 95 °C for 10 min. 14 µL of sample was loaded onto 4-15% Mini-PROTEAN® TGX™ precast gel (Bio-rad, 456-1086), and run in 1× running buffer (0.25 M Tris, 0.2 M glycine, 0.1% (w/v) SDS) for 1.15 h at 130 V. In-gel fluorescence was detected using a Typhoon™ FLA 9500 biomolecular imager (750 V, 100 μm pixels).

**Details of X-ray crystal structures**

Table S1: Crystallographic Statistics for RIPK1-compound complexes

| Data collection | AZ’902 | AZ’320 |
| --- | --- | --- |
| PDB code | 9GTG | 9GTY |
| beamline | BIOMAX-Lund | BIOMAX-Lund |
| wavelength (Å) | 0.976 | 0.976 |
| space group | P2_1_2_1_2_1_ |  |
| cell parameters (Å) | a=47.08, b=96.49, c=129.0 | a=47.70, b=93.48, c=125.53 |
| resolution (Å) | 77.27−2.25 (2.40.−2.26) | 62.77-2.15 (2.33-2.15) |
| redundancy | 6.7 (7.0) | 6.6 (7.0) |
| Completeness (%) | 85.6 (26.4) | 93.9 (73.0) |
| I/σI | 9.0 (1.3) | 13.3 (1.6) |
| R_merge_ (%) | 10.9 (145.9) | 7.2 (214.5) |
| refinement |  |  |
| resolution (Å) | 77.27−2.25 | 62.77-2.15 |
| no. of reflections | 23243 | 23604 |
| percentage of Rfree | 4.8 | 5.0 |
| Rwork/Rfree (%) | 22.0/29.4 | 24.9/29.4 |
| rms deviations |  |  |
| bond lengths | 0.007 | 0.008 |
| bond angles | 1.497 | 0.980 |
| Ramachandran plot (%) |  |  |
| Favoured regions | 90.7 | 93.2 |
| outlier | 1.8 | 2.0 |

**HPLC Chromatograms**

Analytial HPLC was performed on an Agilent 1260 Infinity Series equipped with a column was a Poroshell HPH-C18 3.0x50mm with a flow rate of 0.5 mL/min and an injection volume of 2.00 µL. The system solvent gradients started at 5% MeCN in water ending at 95% MeCN in water after 7 minutes with a 0.1% formic acid additive (**method 1**). For **AZʹ320** (HPLC) analysis was carried out on an Alliance HPLC 2695 system (Waters^®^, Ireland), equipped with an autosampler and photodiode array detector 2996 (Waters^®^, Ireland). A Waters^®^ SunFire^TM^ C18 5 μm (2.1 x 100 mm) reverse phase column was used with a constant flow rate of 0.3 mL min^-1^ and a gradient method of 30 min from 70:30 H_2_O (with 0.1% formic acid):MeCN to 5:95 H_2_O (with 0.1% formic acid):MeCN (**method 2**).

**AZʹ902** (Rt = 4.120 min; 95.0%) (**method 1**)
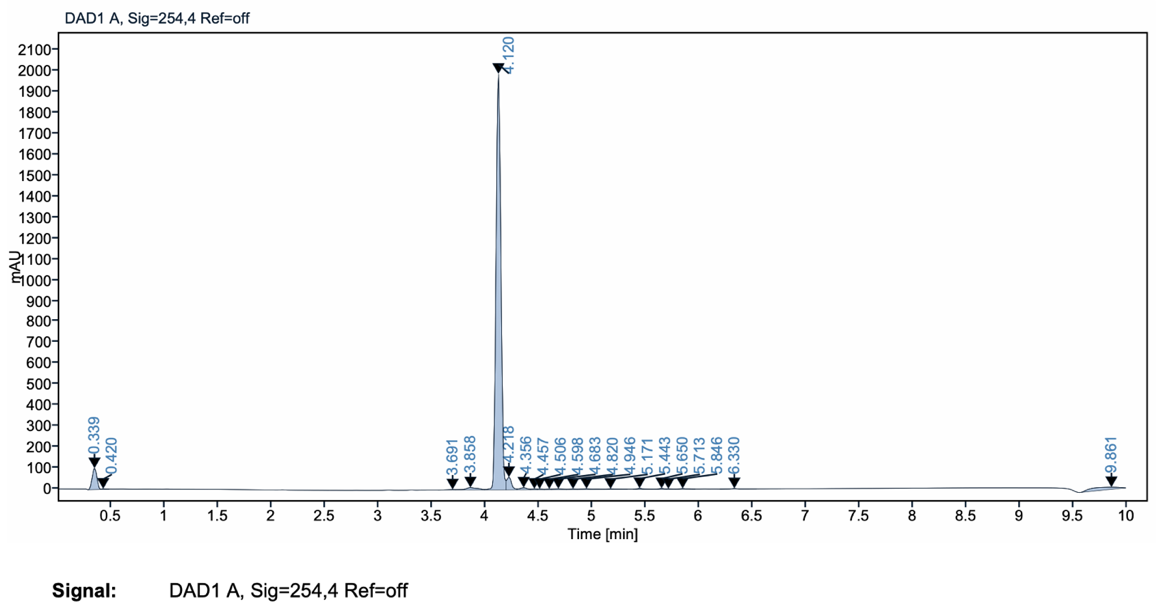


**AZʹ320** (Rt = 2.367 min; 99.9%) (**method 2**)


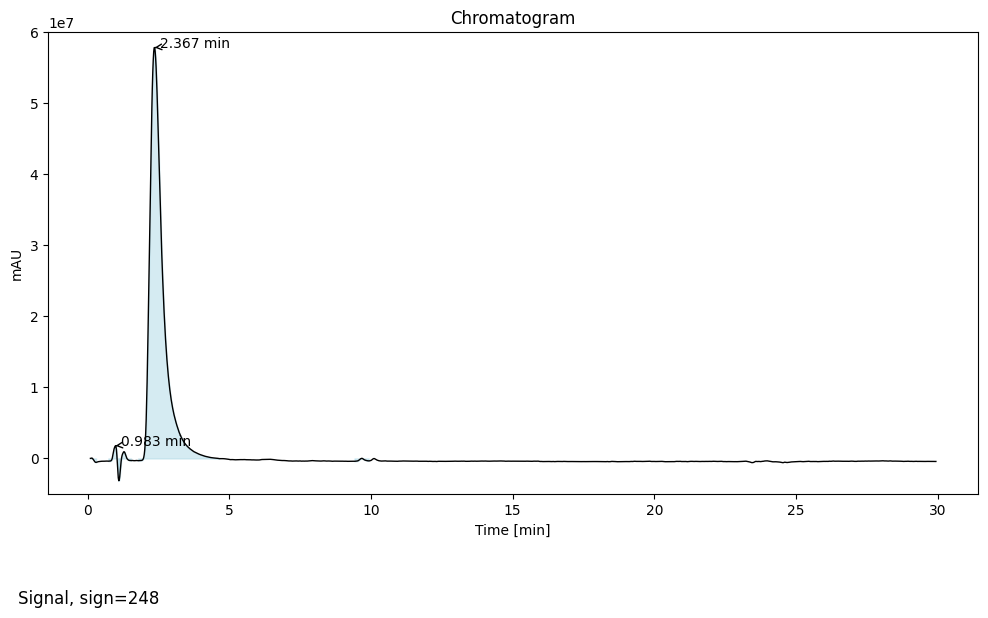


**7PQYnD** (Rt = 5.360 min; 99.1%) (**method 1**)


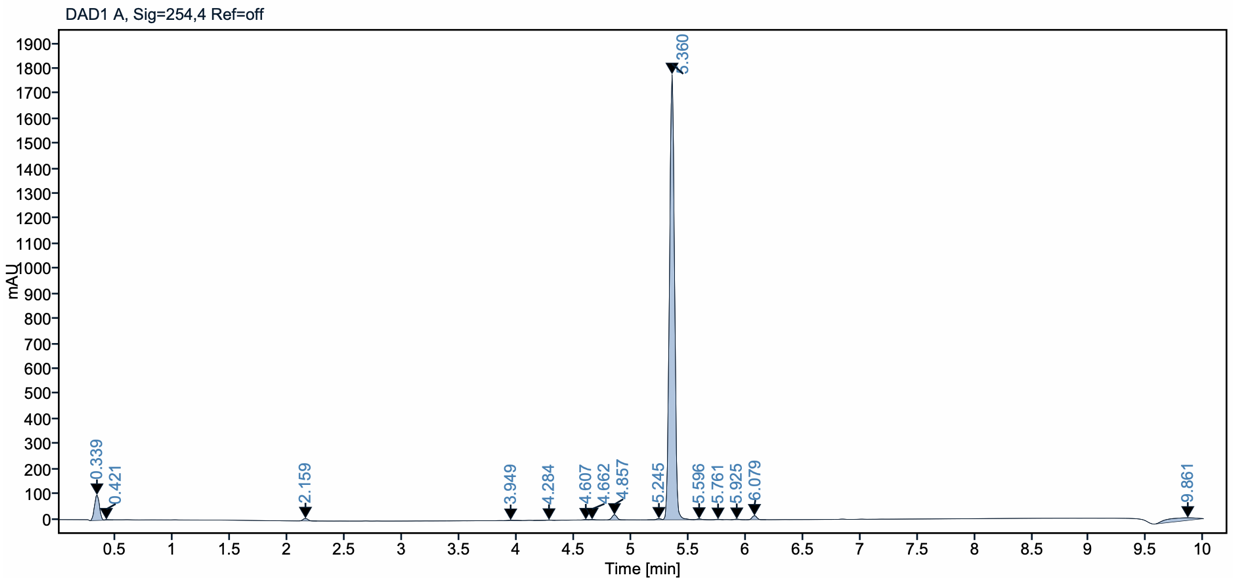


**Chiral HPLC detection of AZʹ902 Enantiomers**

A racemic sample of **AZ'902** and **1** was dissolved in neat MeOH to yield a final sample concentration of ~1.5 mg/mL. Analytical HPLC runs were conducted using an Agilent 1260 Infinity II LC with DAD and MSD XT, equipped with a CHIRALPAK IG-U column (5 μm particle size, column size: 3.0 mm x 100 mm L; column temperature: 25 ºC; flow rate: 0.6 mL/min; injection volume: 1 μL; solvent: MeCN:water = 45:55 isocratic; pressure: 420 bar). The chromatogram seen below was thus obtained. The peaks at 7.5 min and 8.4 min represent **AZ'902** and **1**, respectively.

**
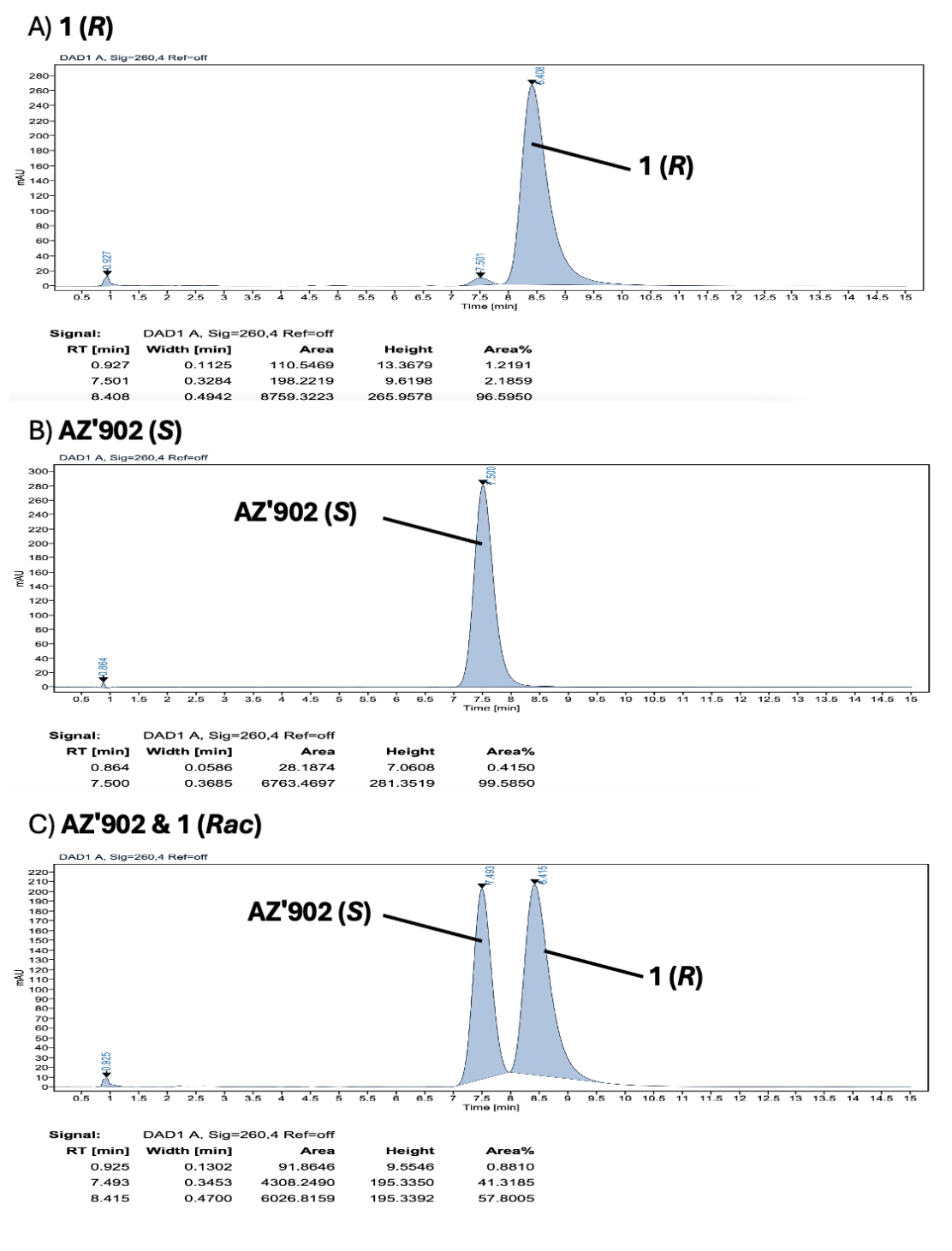
**

Stack of Chiral HPLC chromatograms of enantiomers **AZ’902** and **1**, racemic mixture at 260 nm

**NMR Spectra of Synthesized Compounds**


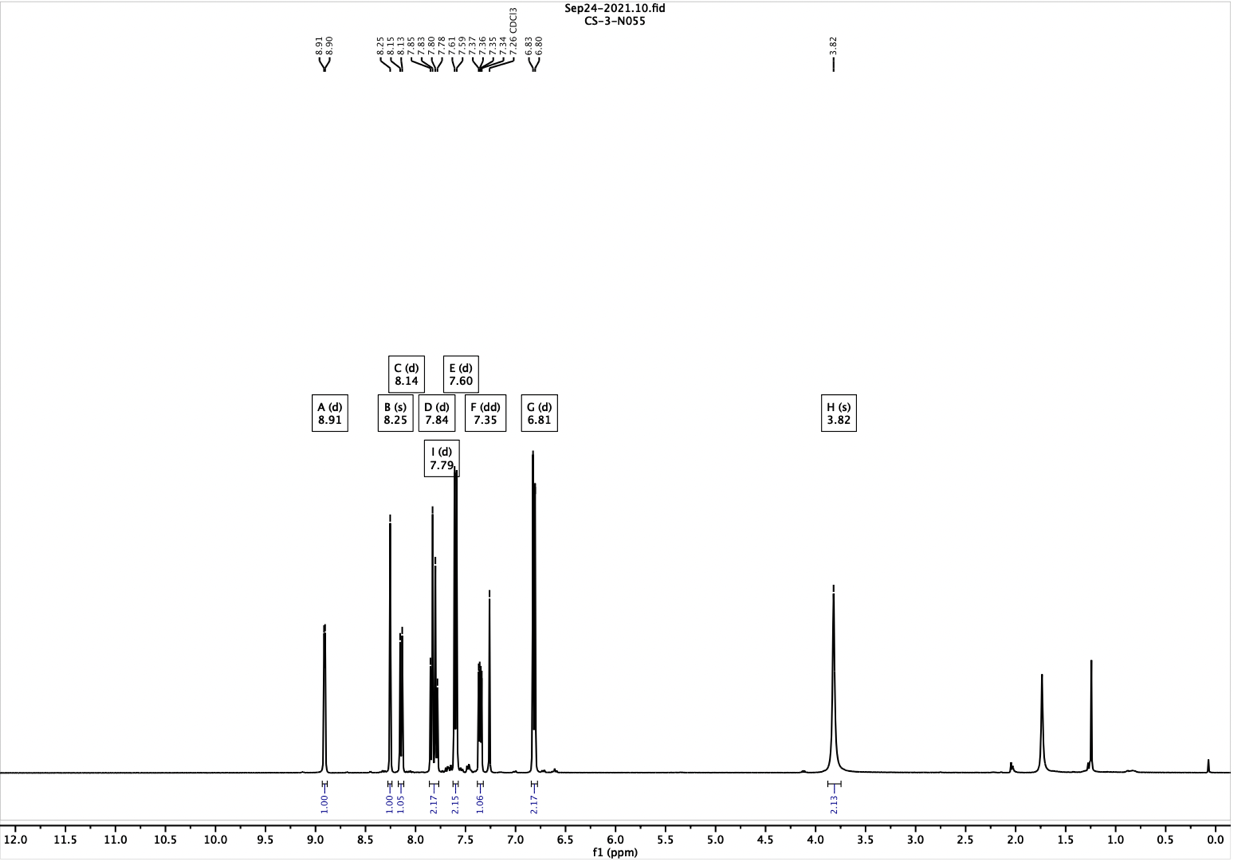


^1^H NMR spectrum of **13** in DMSO-*d*_6_ at 400 MHz.


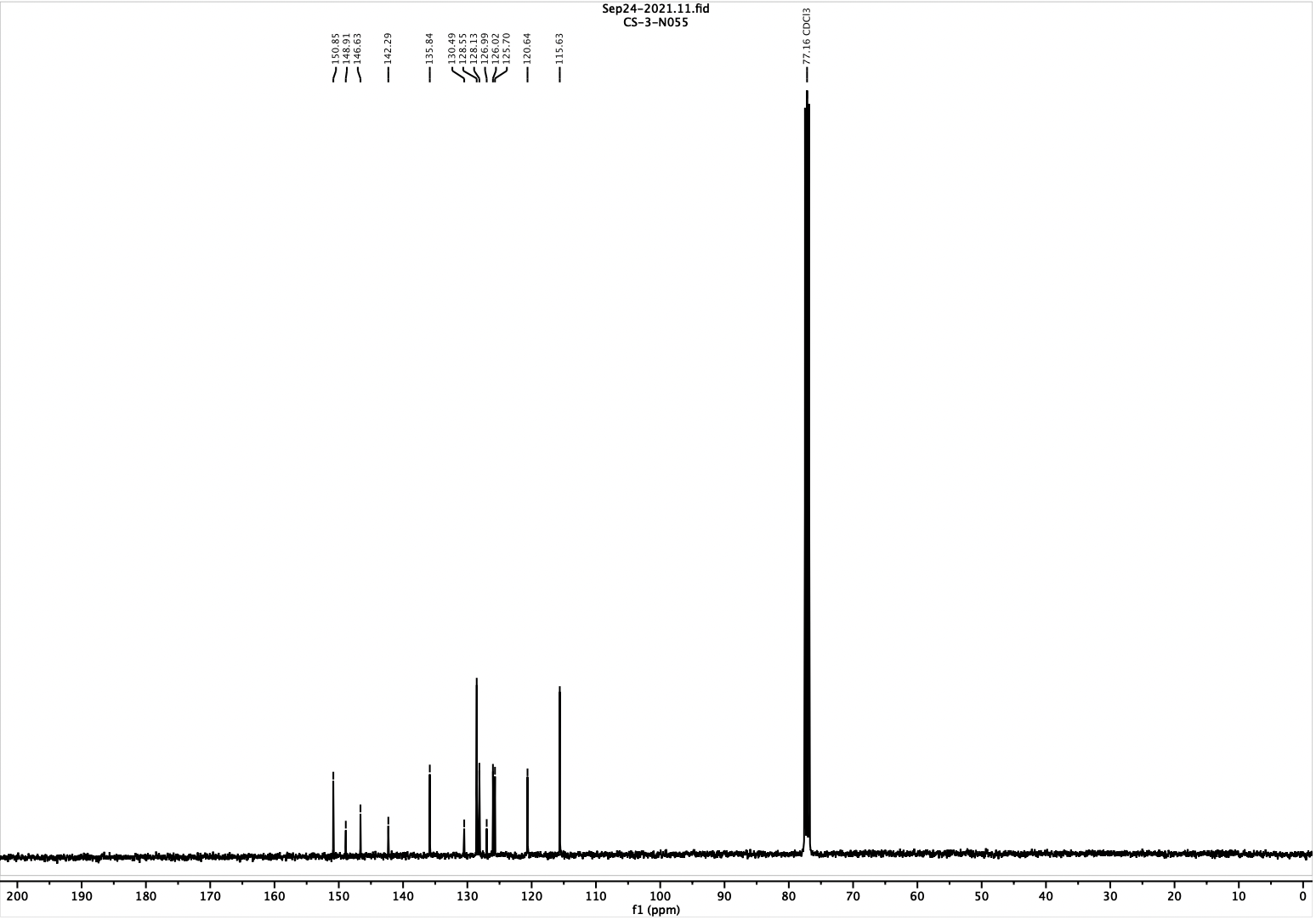


^13^C NMR spectrum of **13** in DMSO-*d*_6_ at 101 MHz.


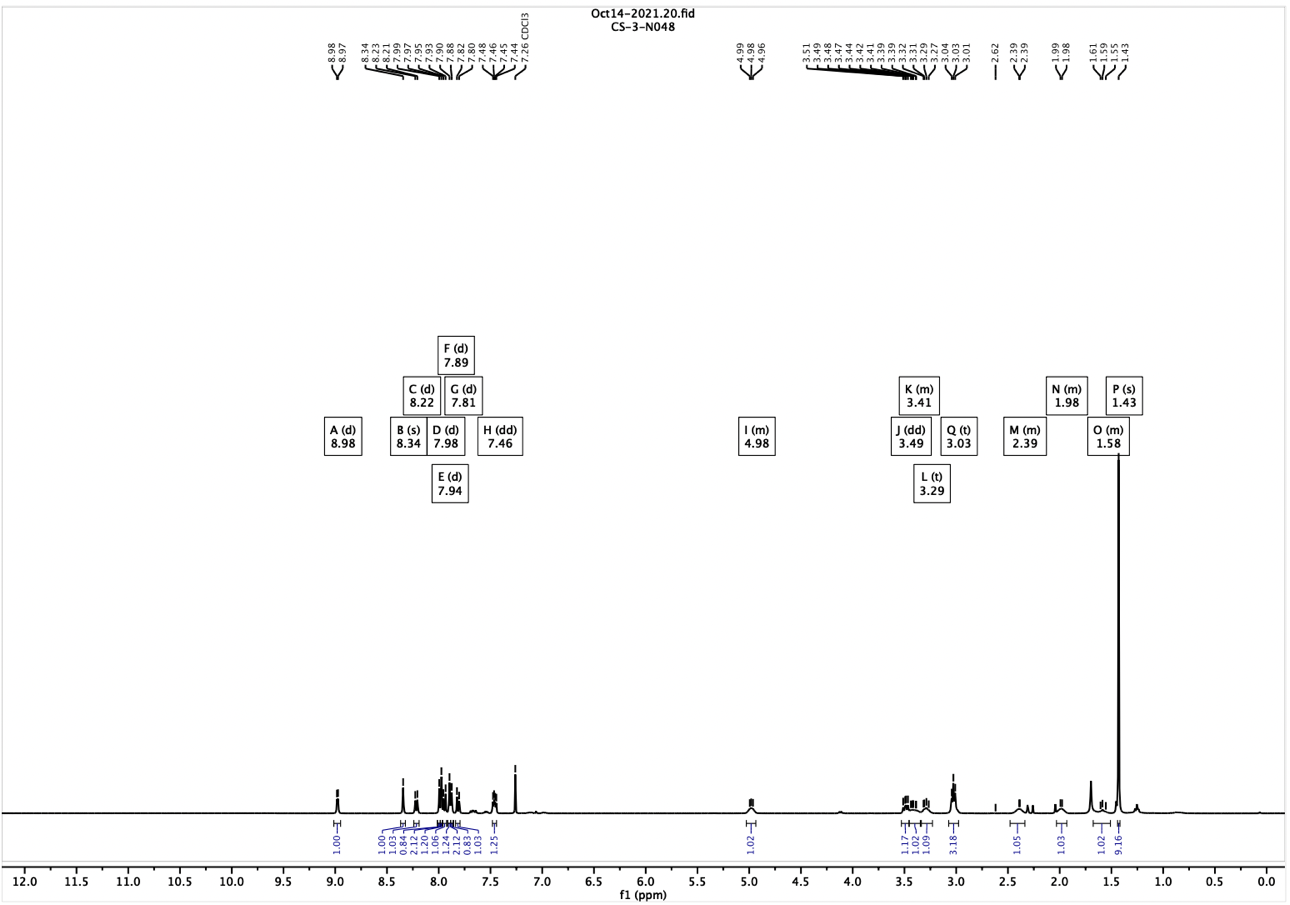


^1^H NMR spectrum of **15** in CDCl_3_ at 400 MHz.

^
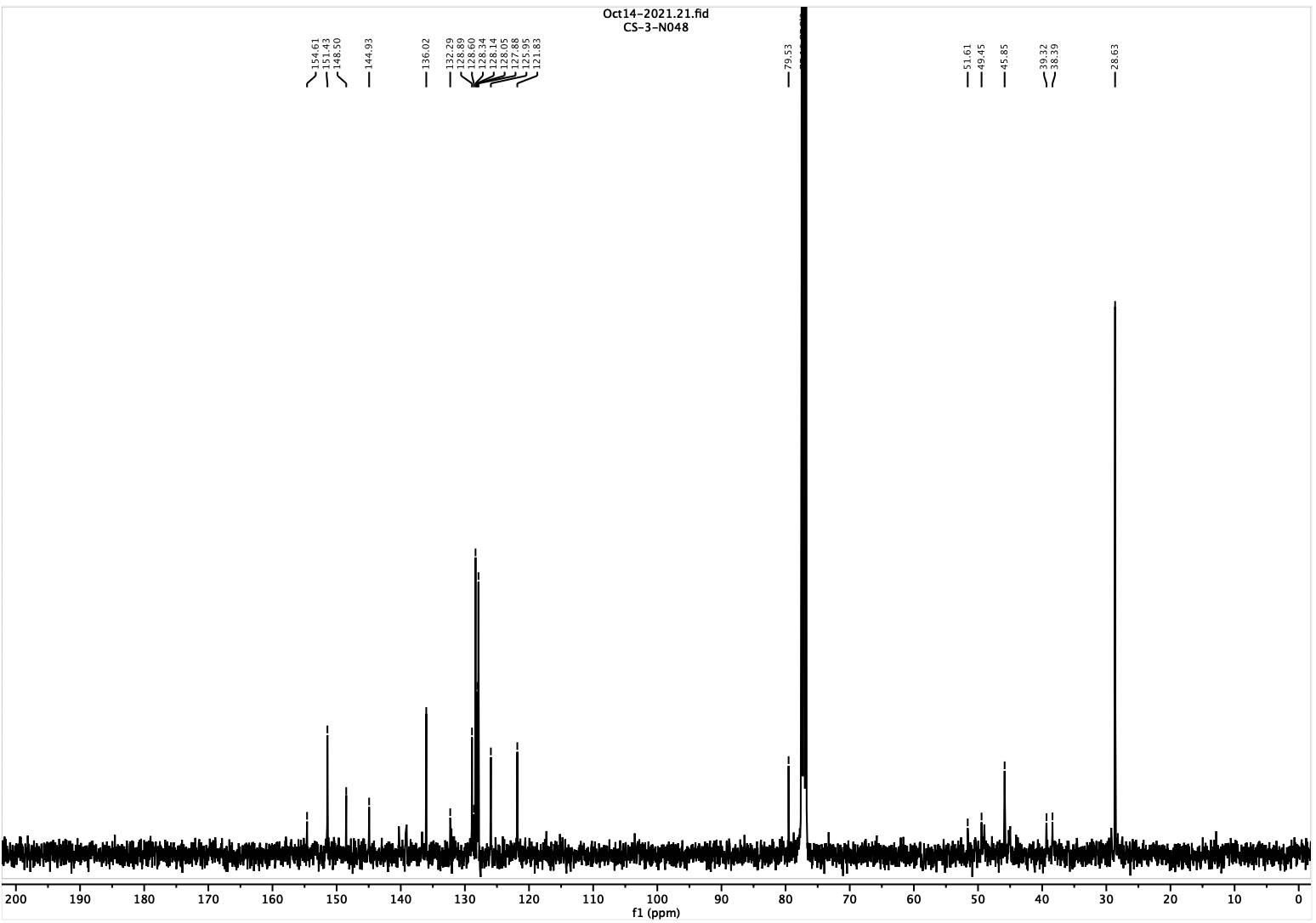
^

^13^C NMR spectrum of **15** in CDCl_3_ at 101 MHz.

^
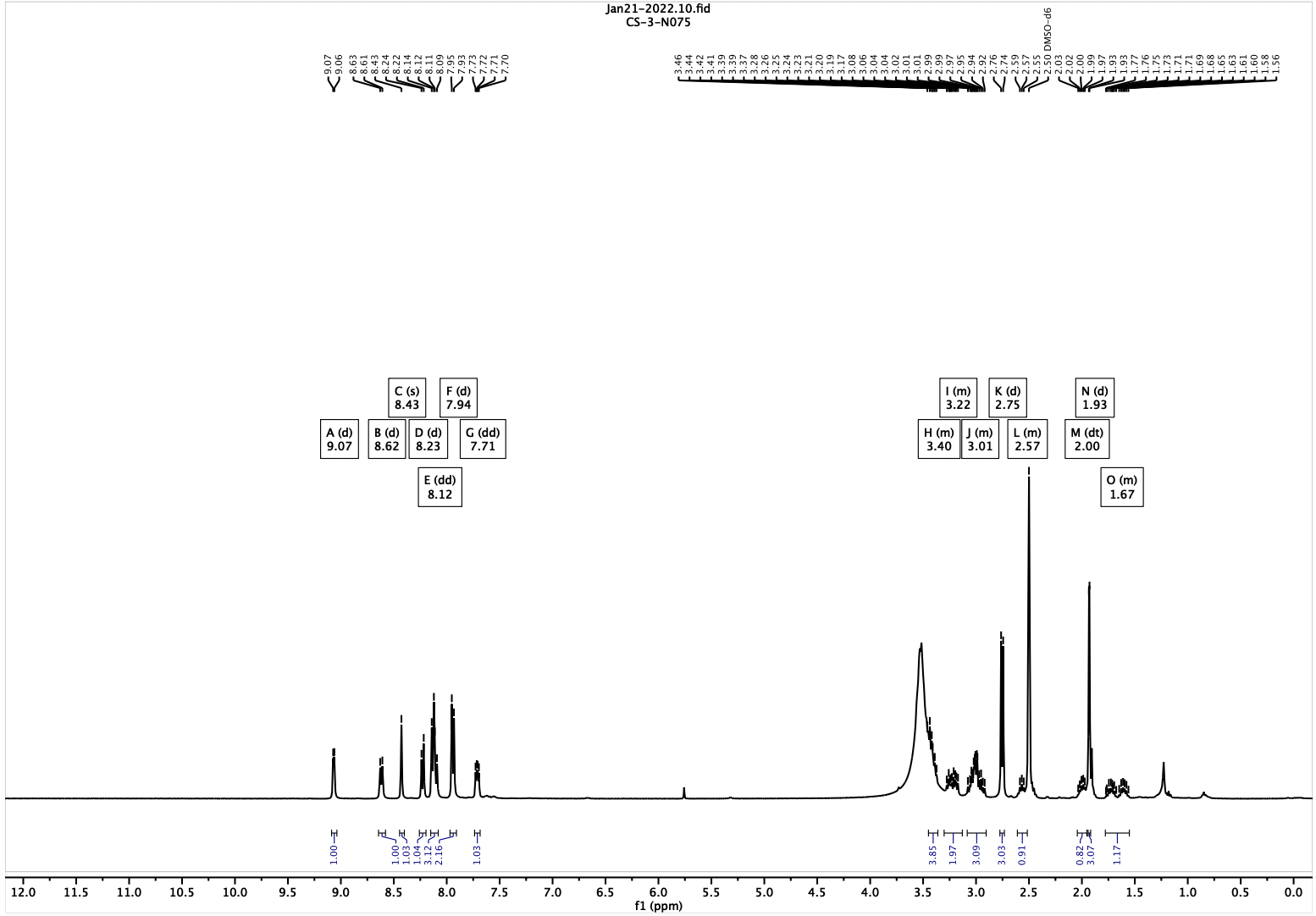
^

^1^H NMR spectrum of **1** in DMSO-*d*_6_ at 400 MHz.


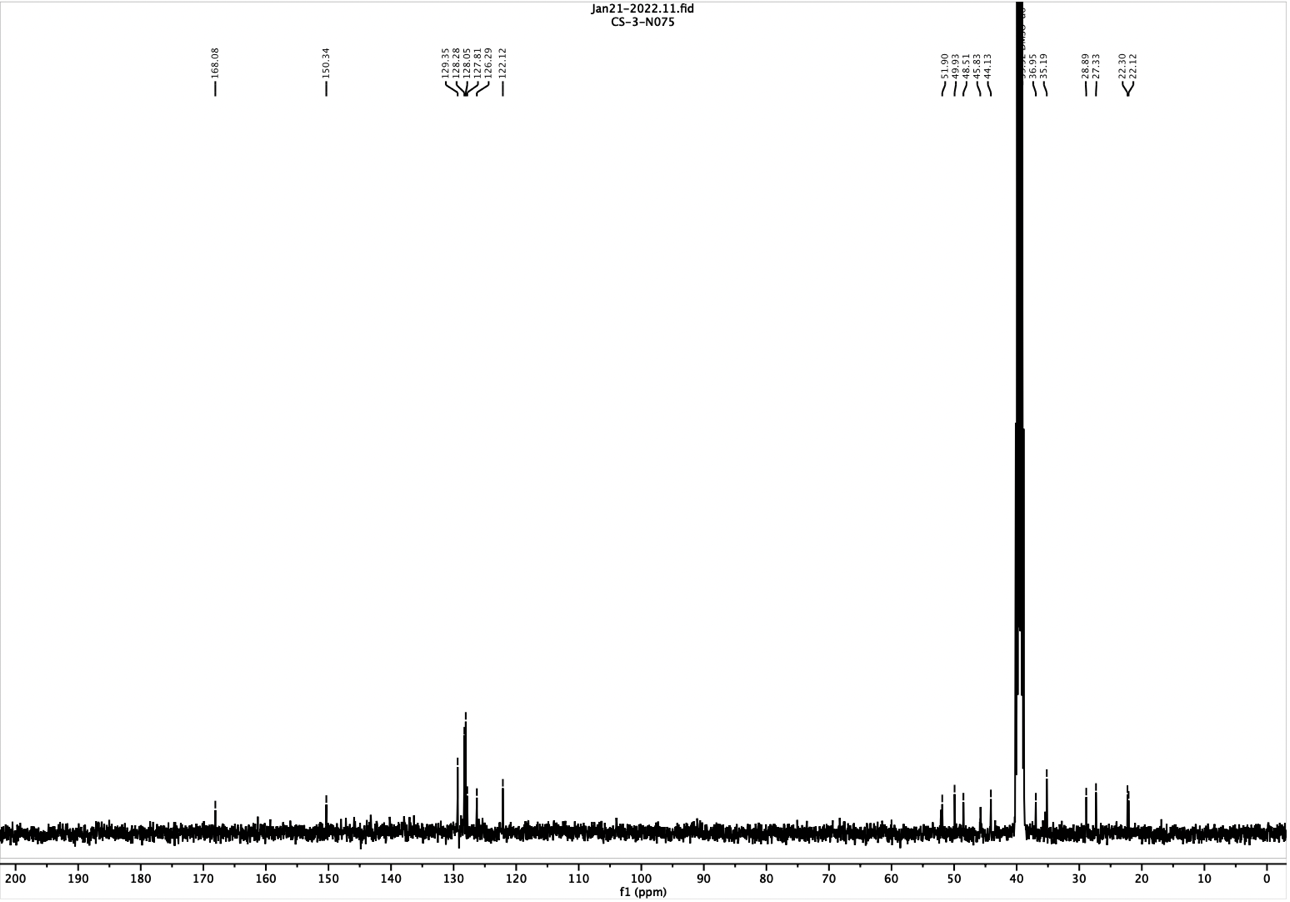


^13^C NMR spectrum of **1** in DMSO-*d*_6_ at 101 MHz.

**
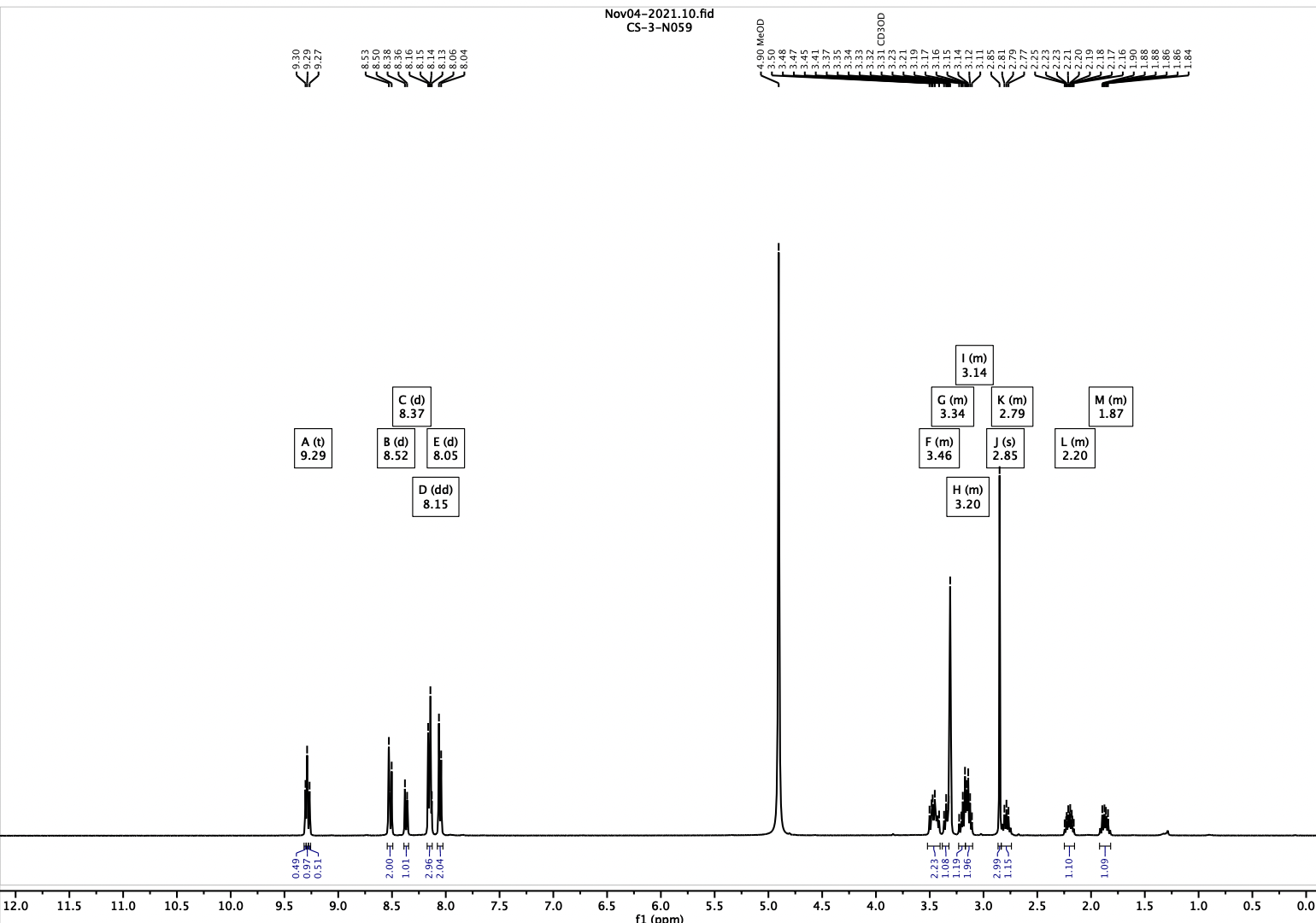
**

^1^H NMR spectrum of **2** in MeOD at 400 MHz.

**
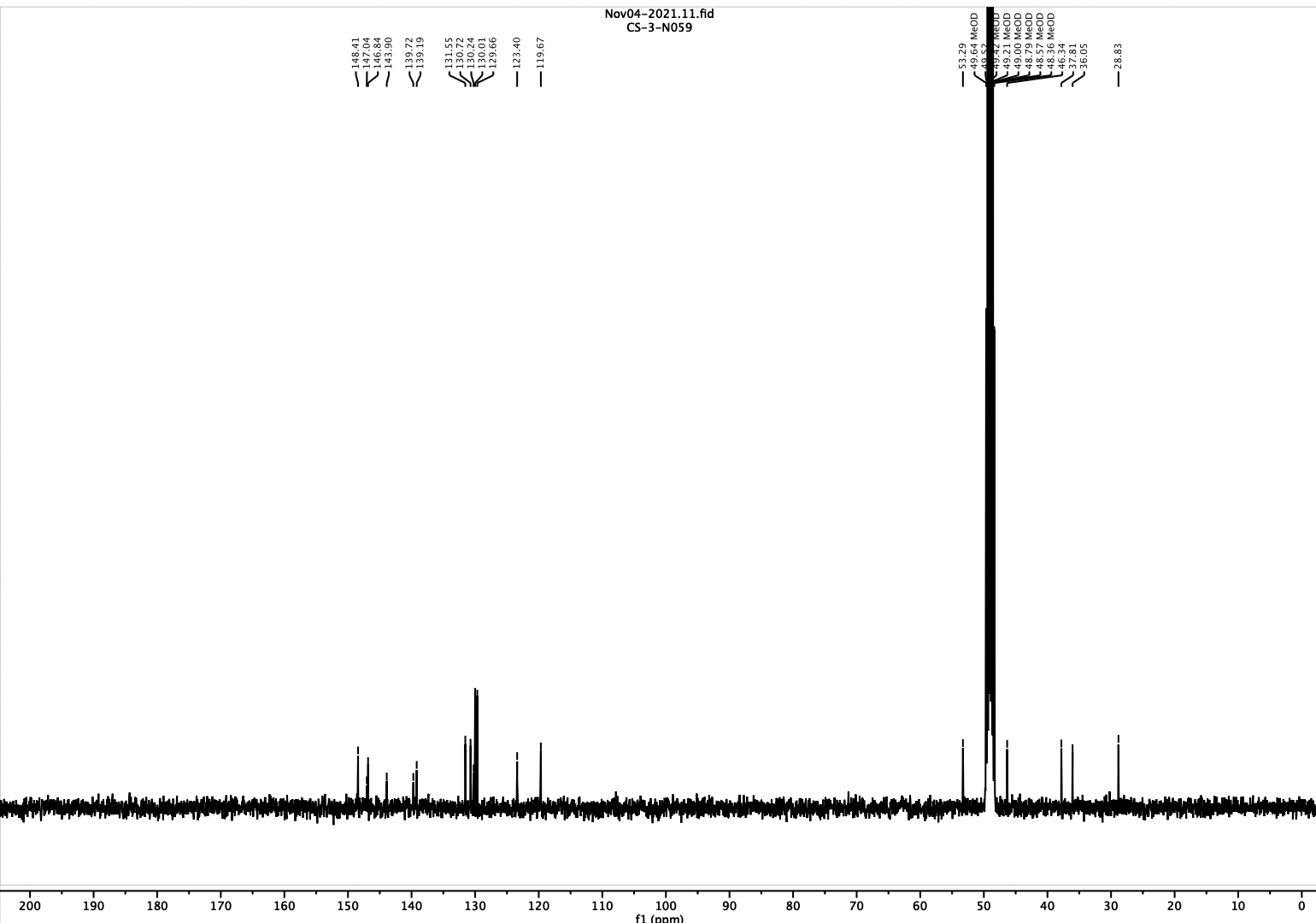
**

^13^C NMR spectrum of **2** in MeOD at 101 MHz.

**
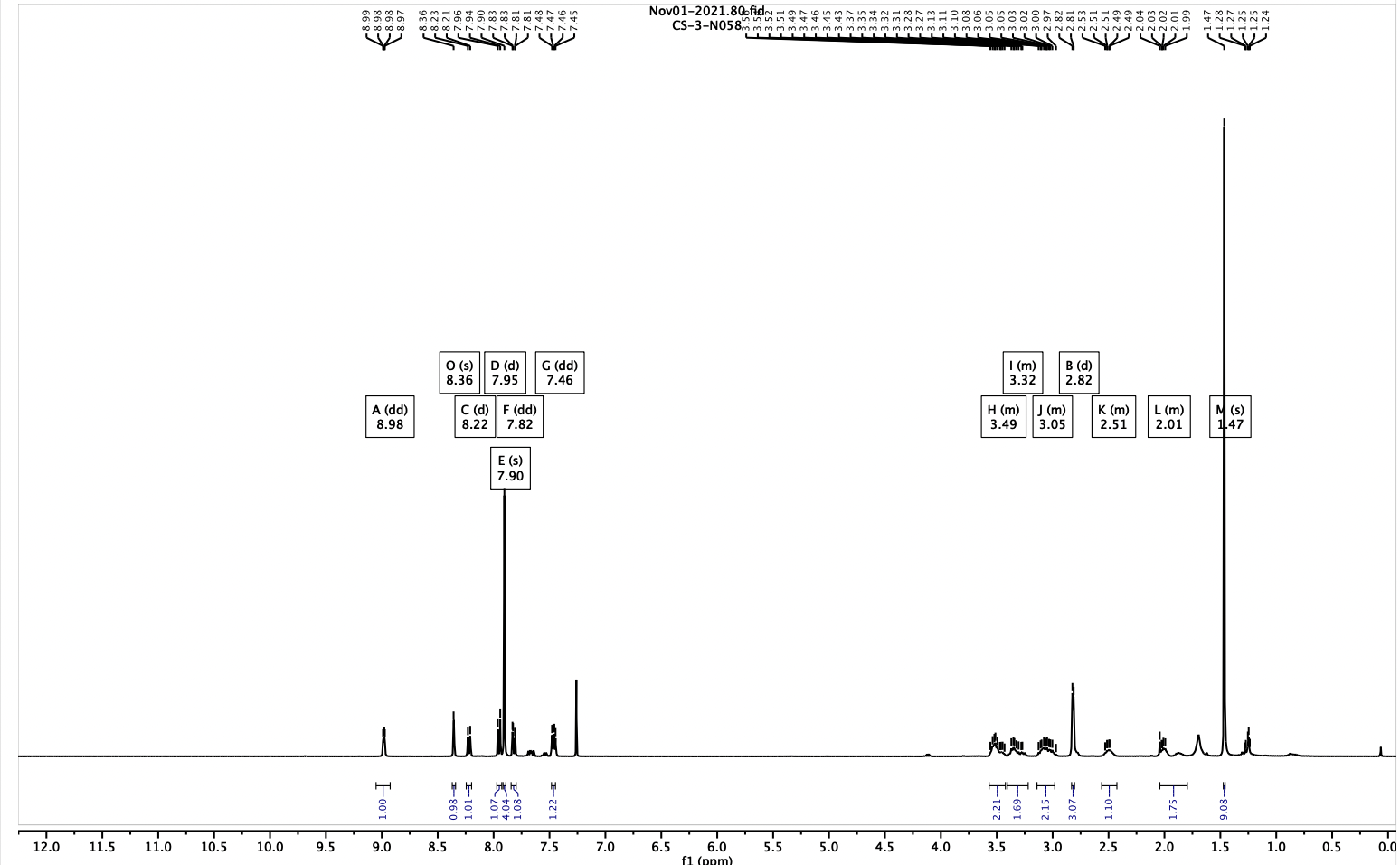
**

^1^H NMR spectrum of **3** in CDCl_3_ at 400 MHz.

**
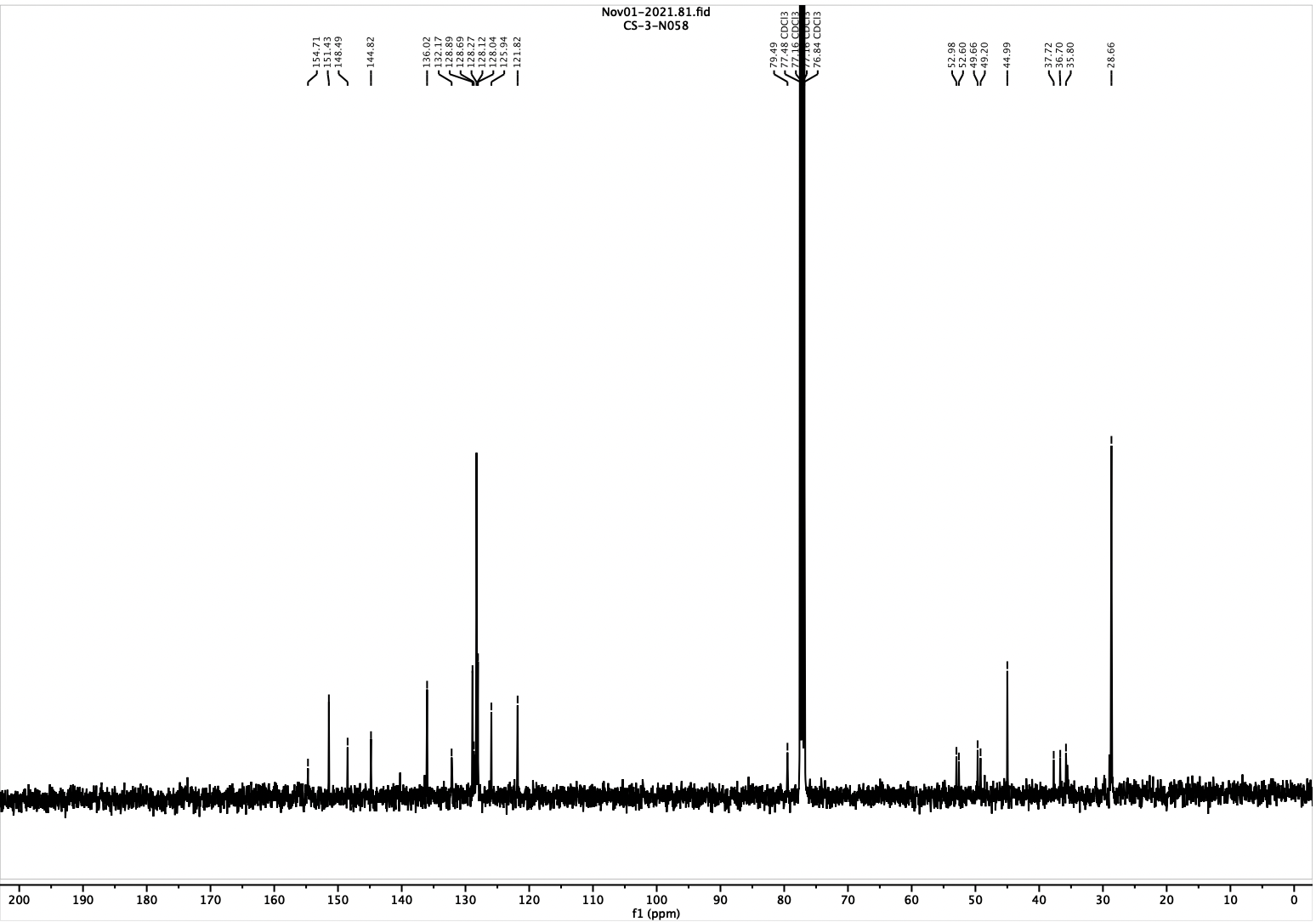
**

^13^C NMR spectrum of **3** in CDCl_3_ at 101 MHz.

^
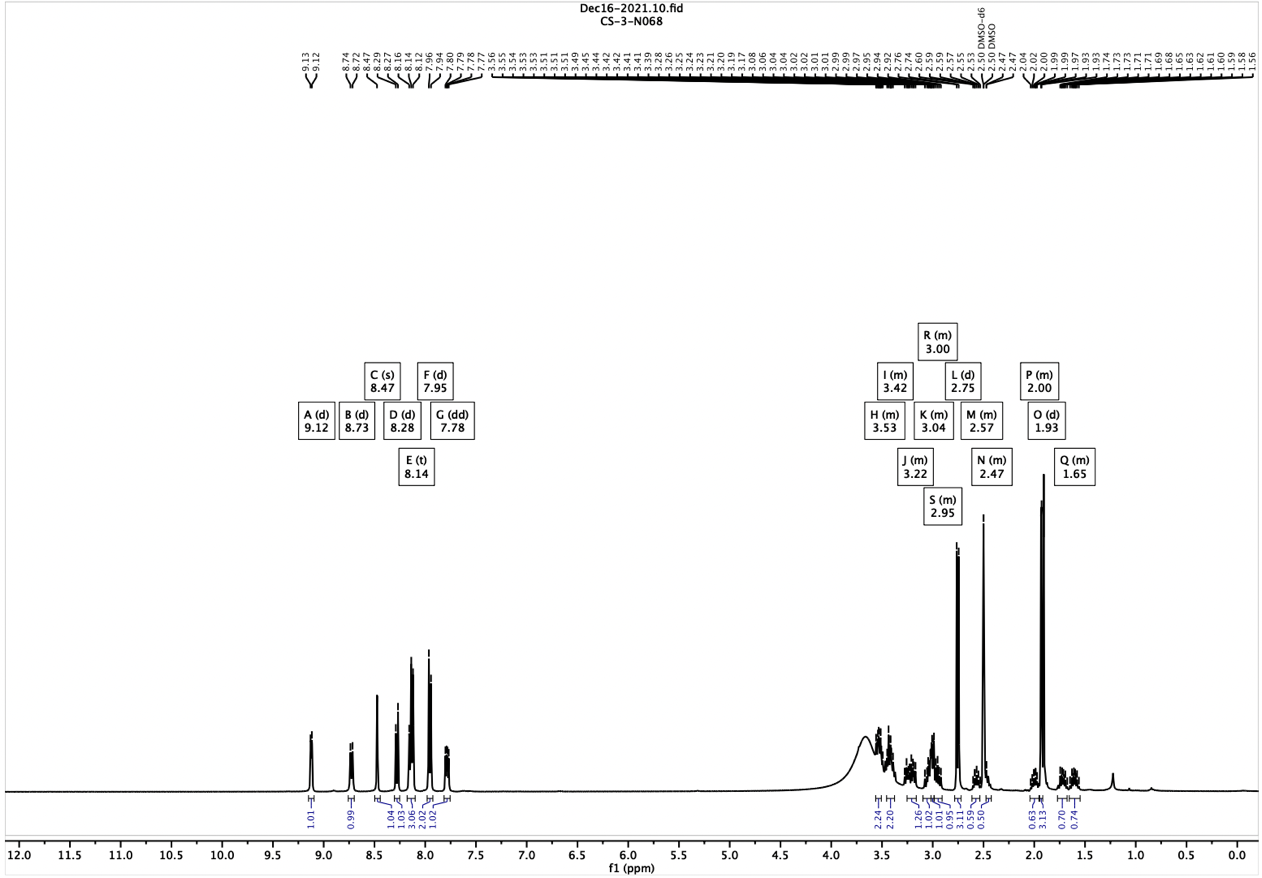
^

^1^H NMR spectrum of **AZ'902** in DMSO-*d*_6_ at 400 MHz.


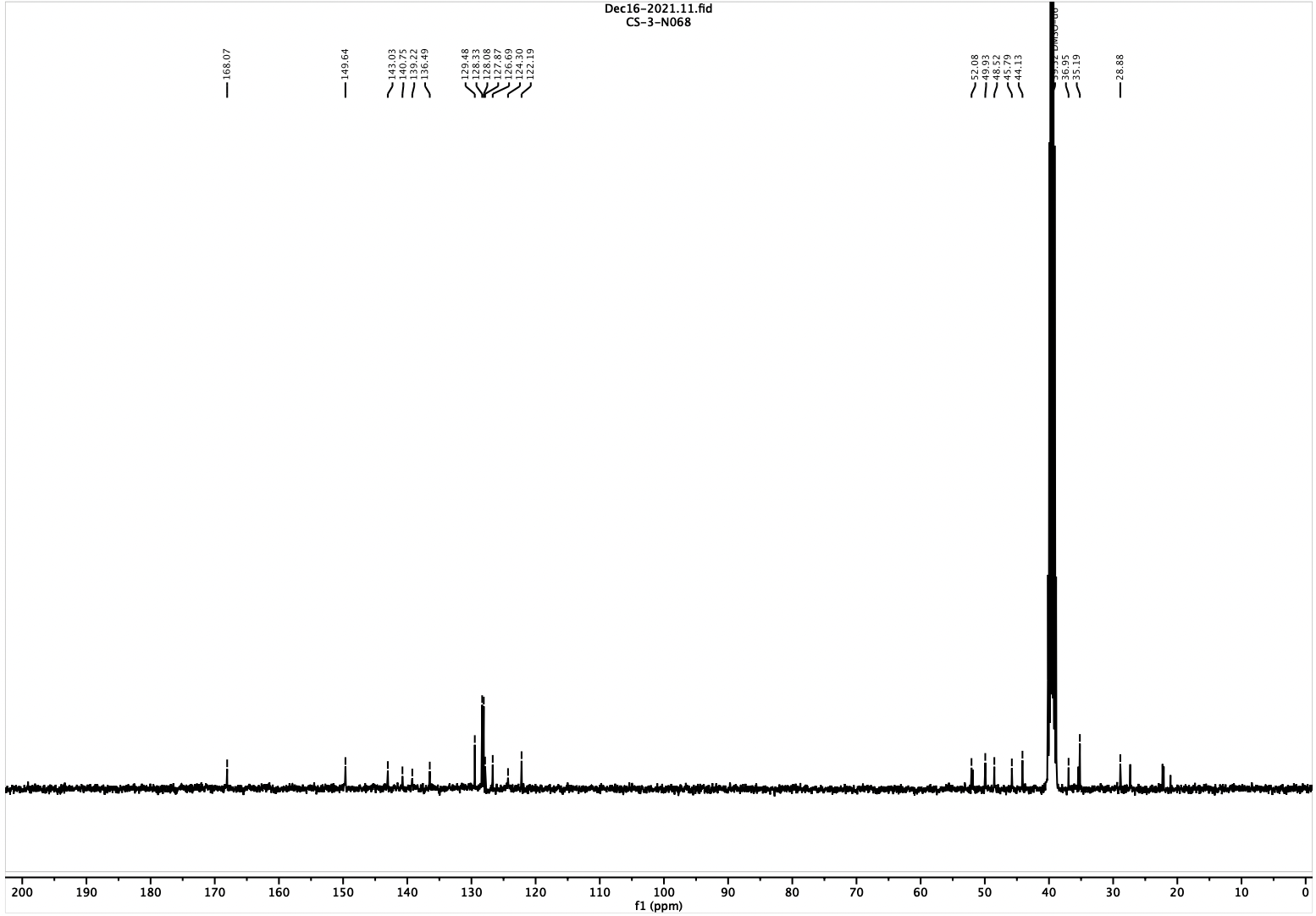


^13^C NMR spectrum of **AZ'902** in DMSO-*d*_6_ at 101 MHz.

**
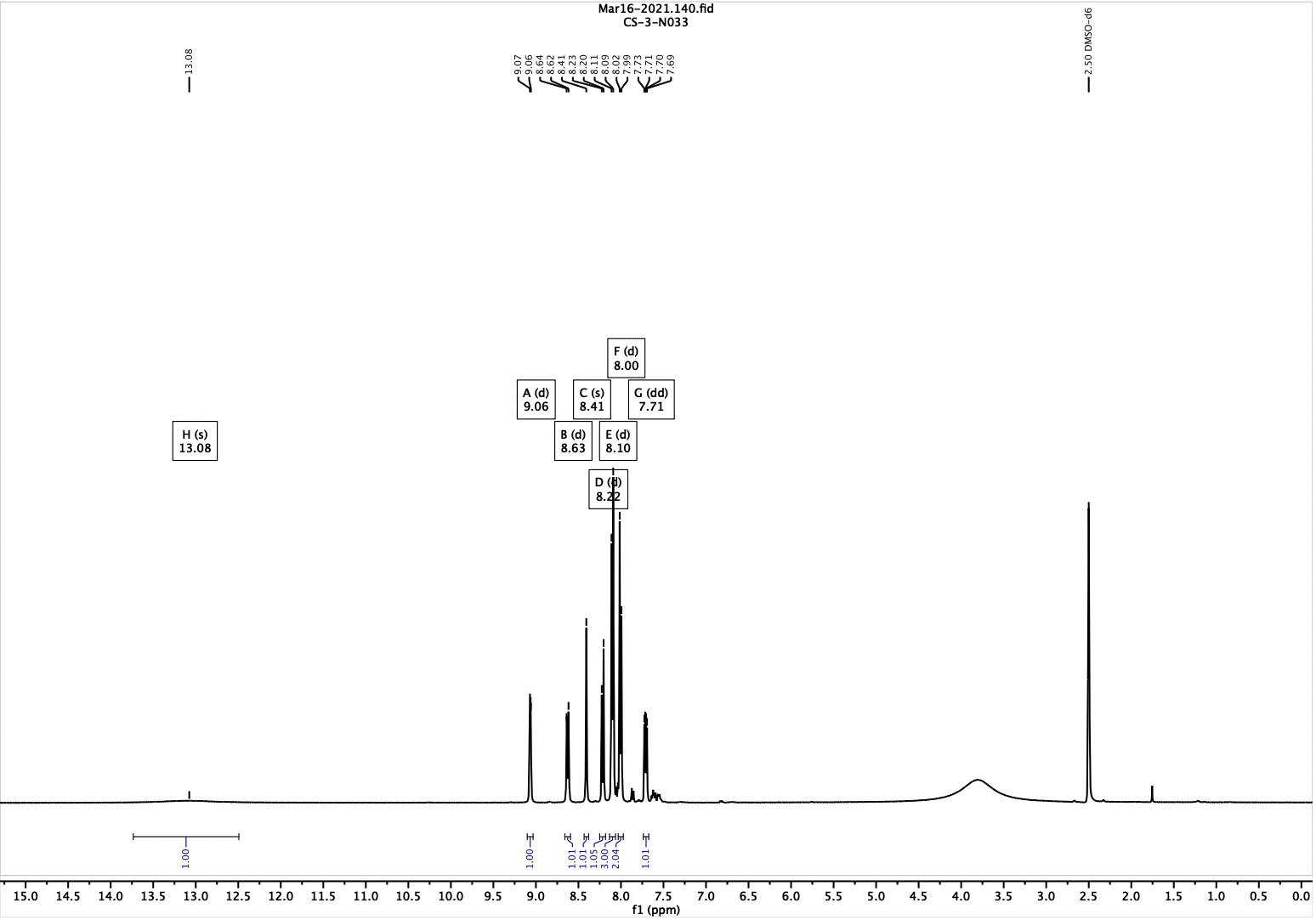
**

^1^H NMR spectrum of **16** in DMSO-*d*_6_ at 400 MHz.


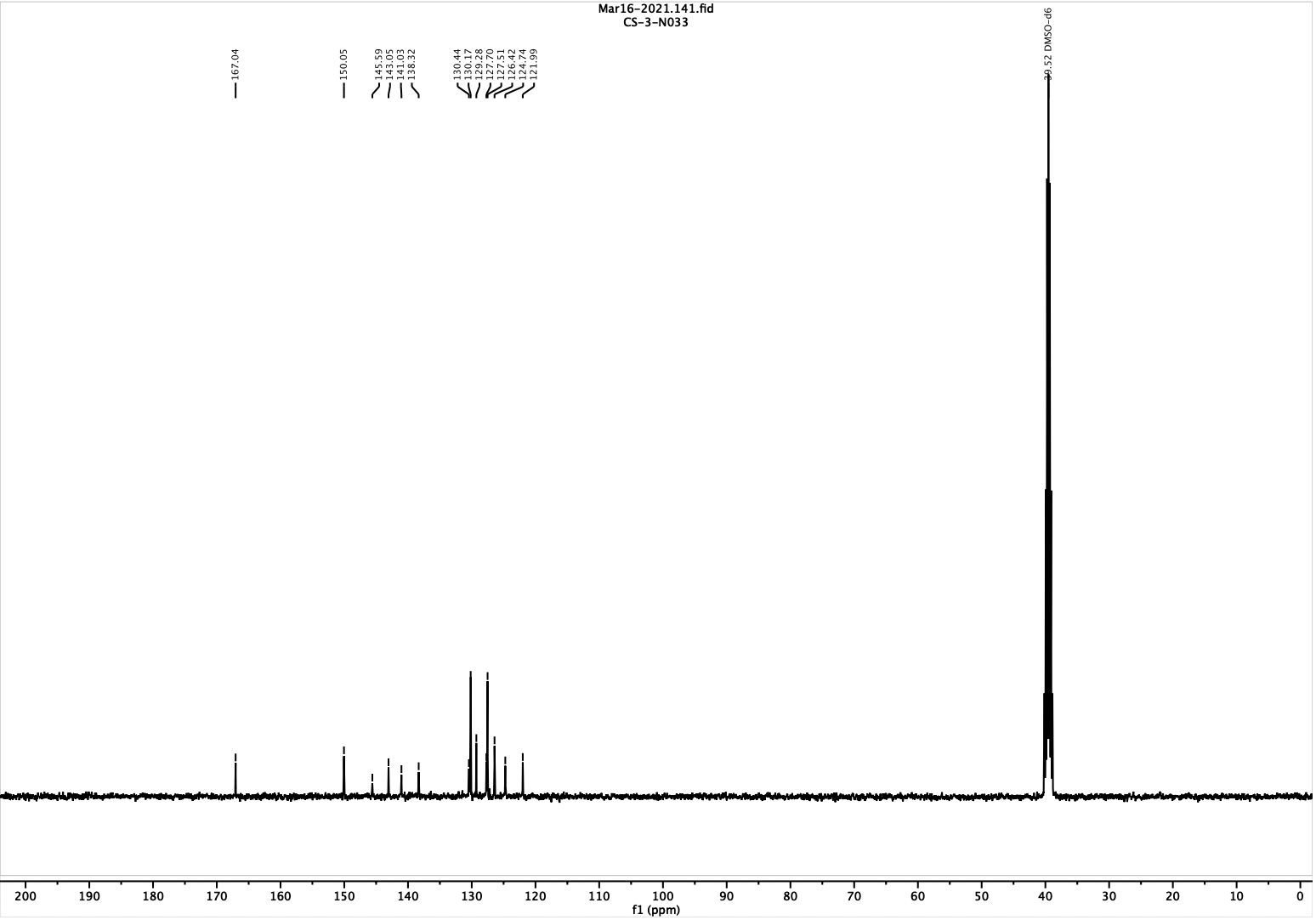


^13^C NMR spectrum of **16** in DMSO-*d*_6_ at 101 MHz.

**
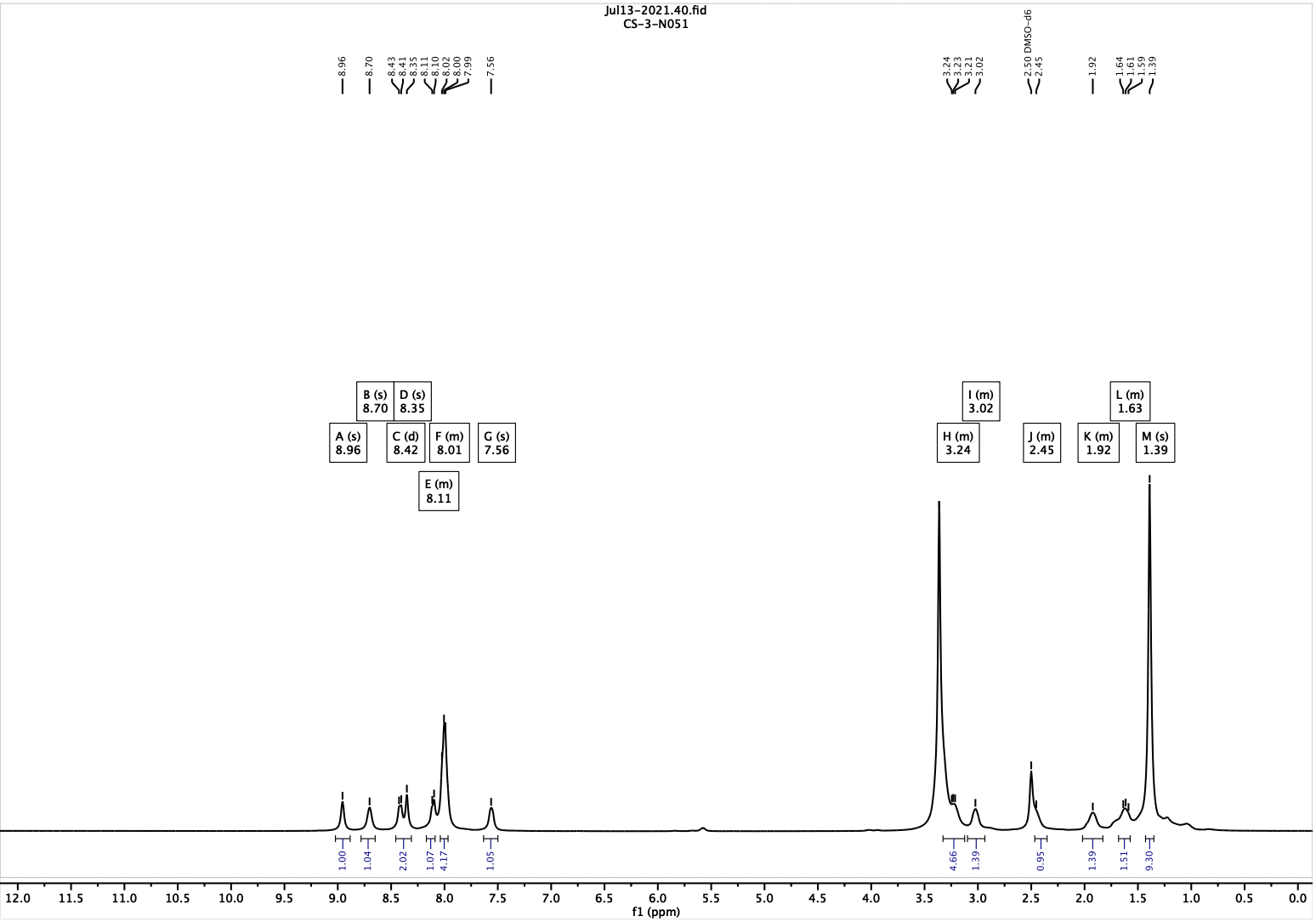
**

^1^H NMR spectrum of **4** in DMSO-*d*_6_ at 400 MHz.


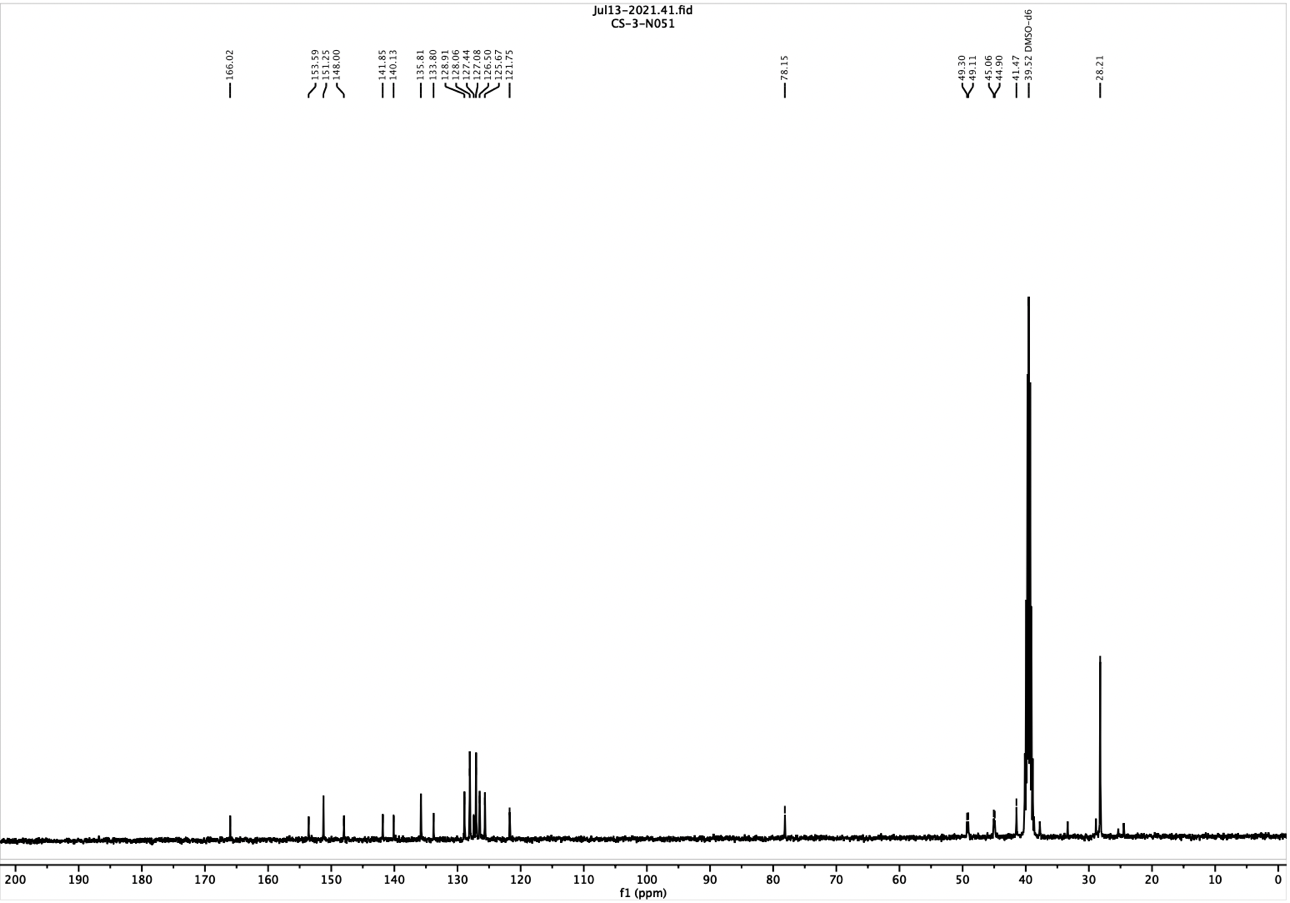


^13^C NMR spectrum of **4** in DMSO-*d*_6_ at 101 MHz.

**
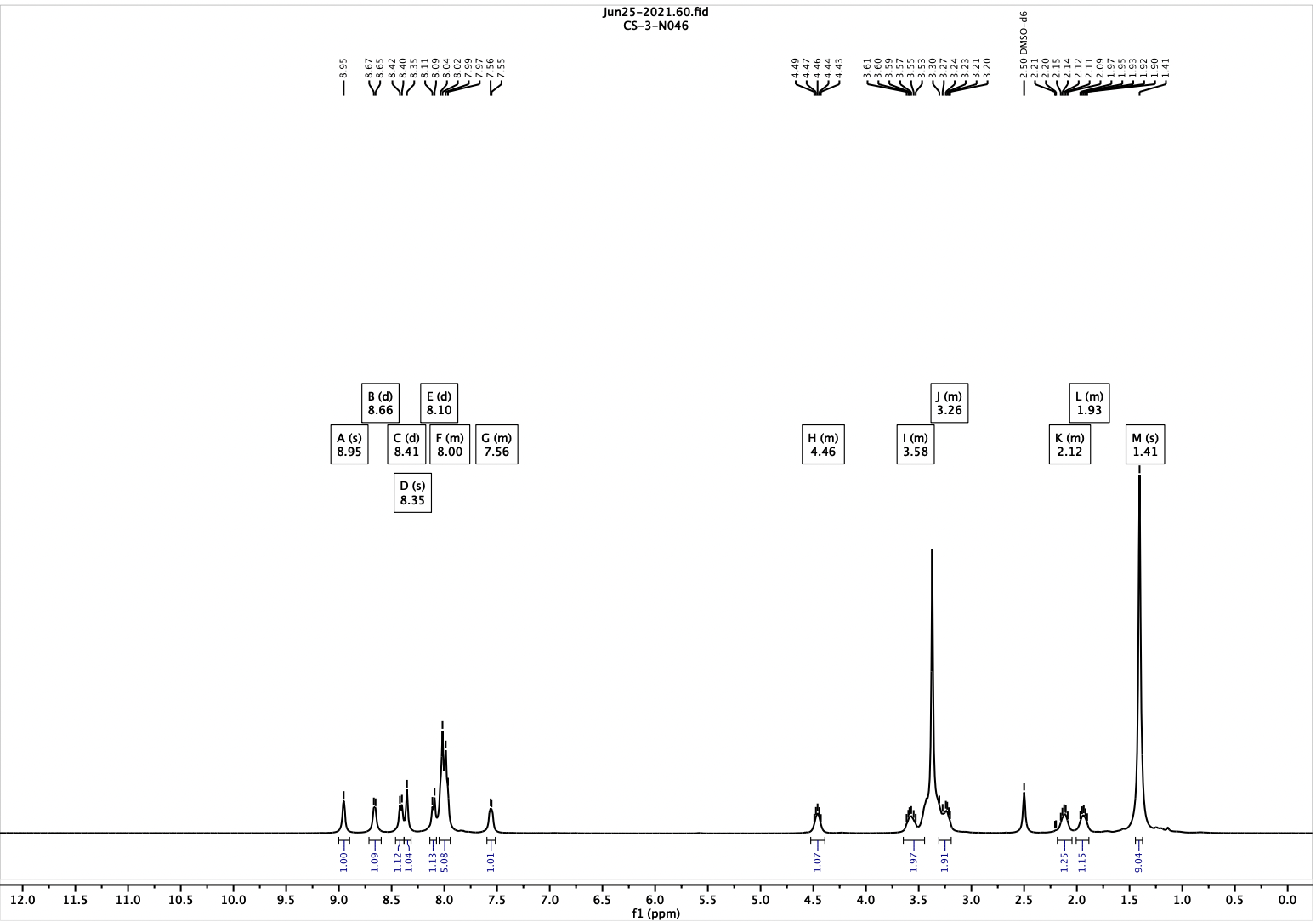
**

^1^H NMR spectrum of **5** in DMSO-*d*_6_ at 400 MHz.


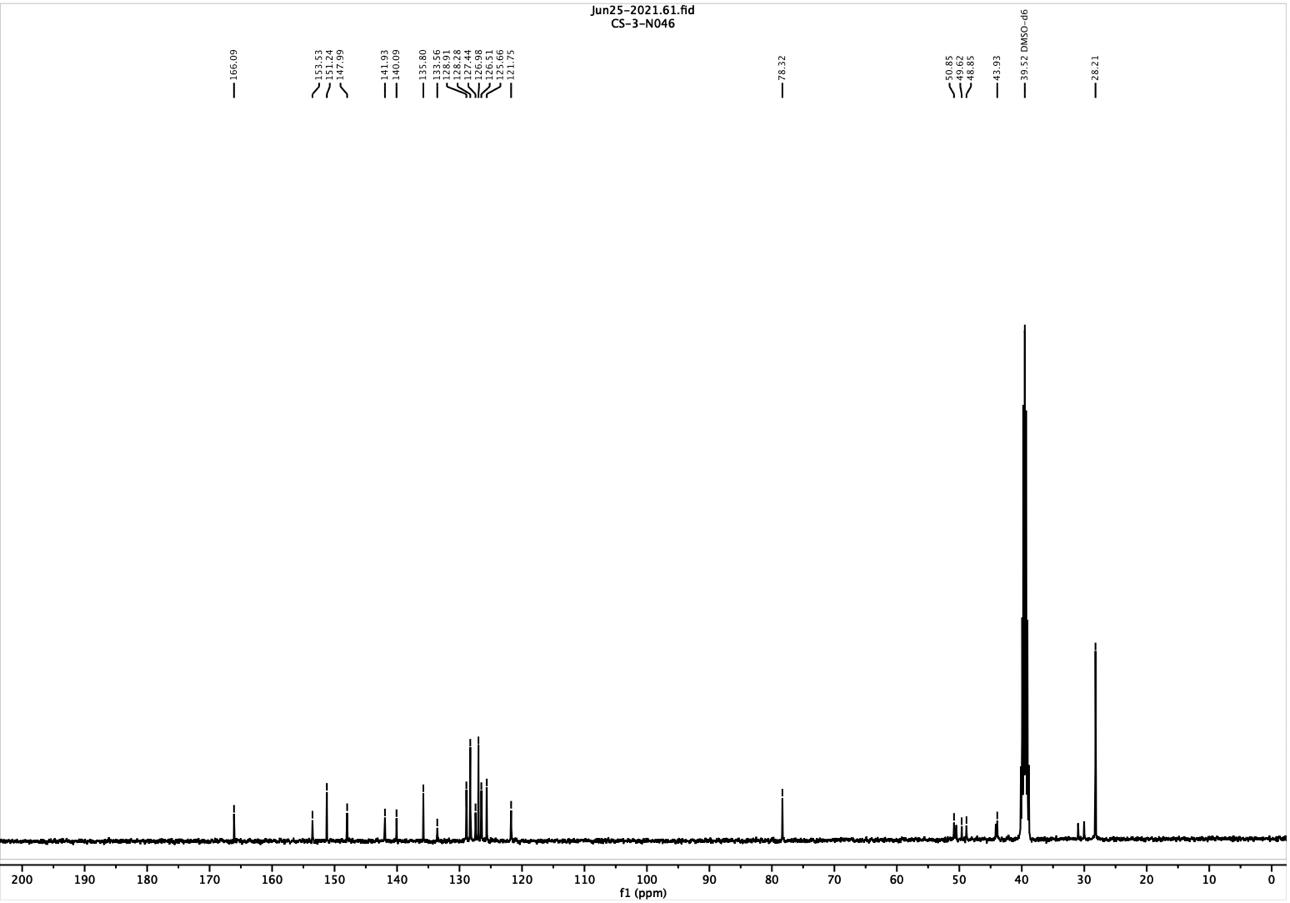


^13^C NMR spectrum of **5** in DMSO-*d*_6_ at 101 MHz.

**
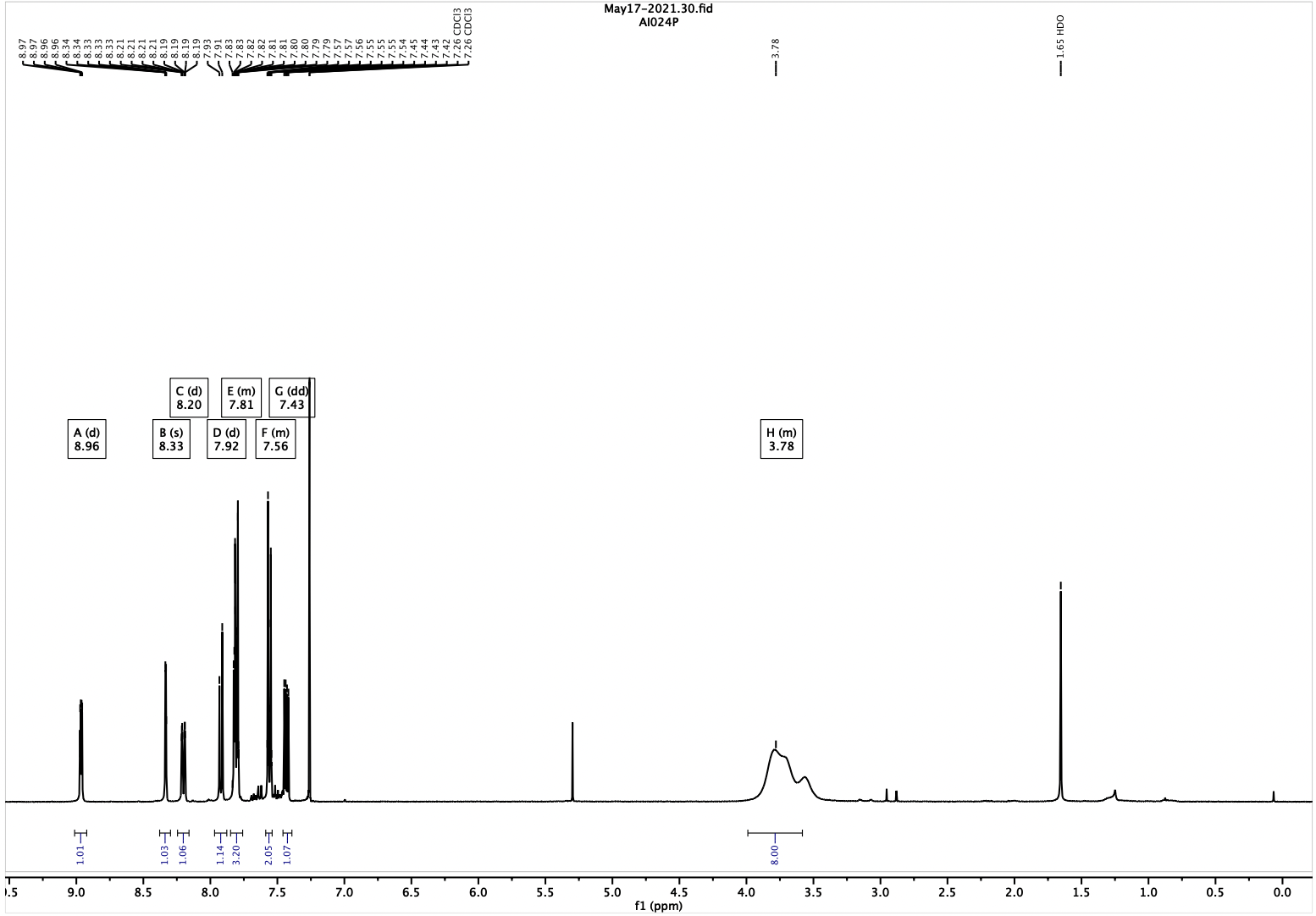
**

^1^H NMR spectrum of **6** in CDCl_3_ at 400 MHz.


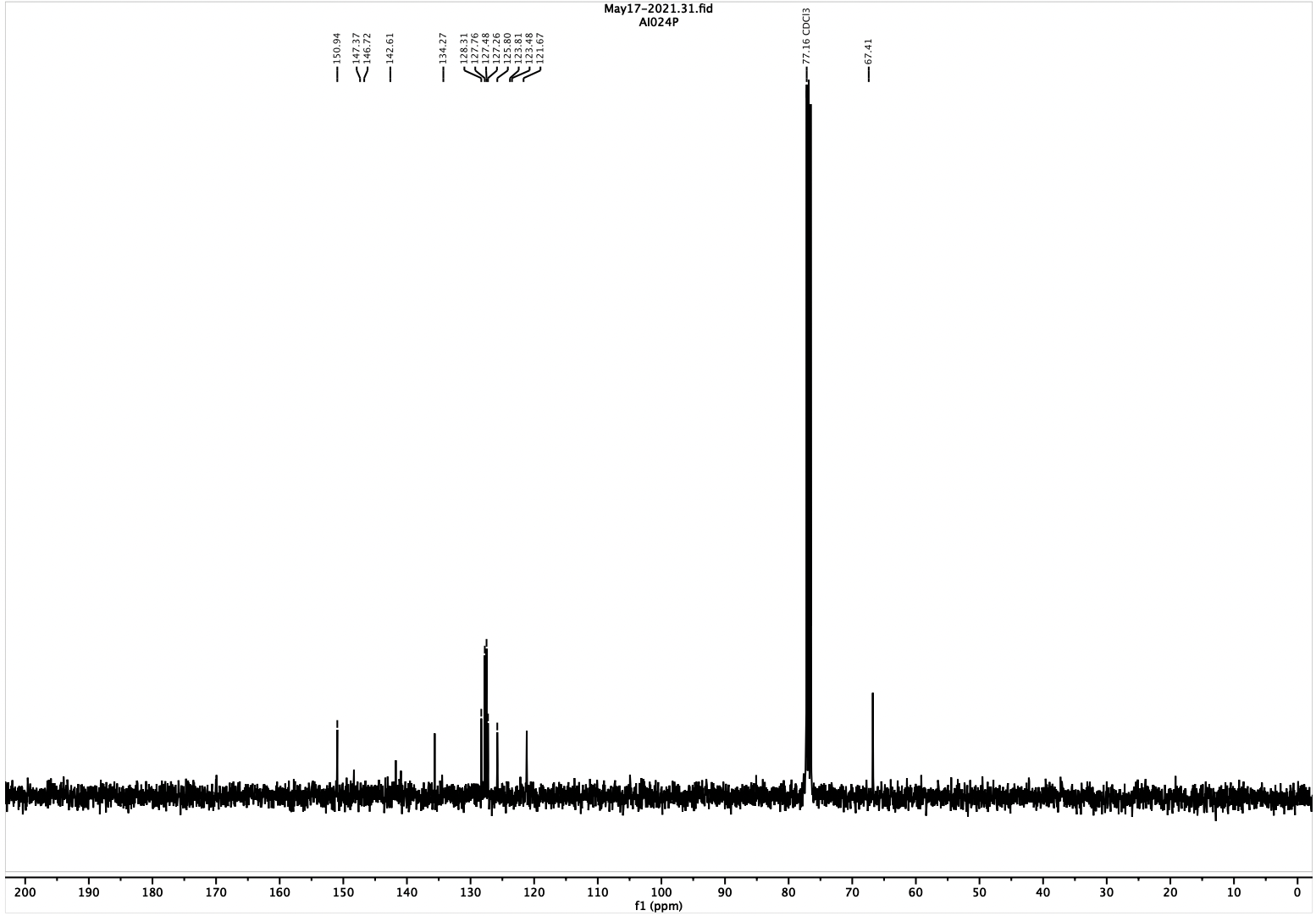


^13^C NMR spectrum of **6** in CDCl_3_ at 101 MHz.

**
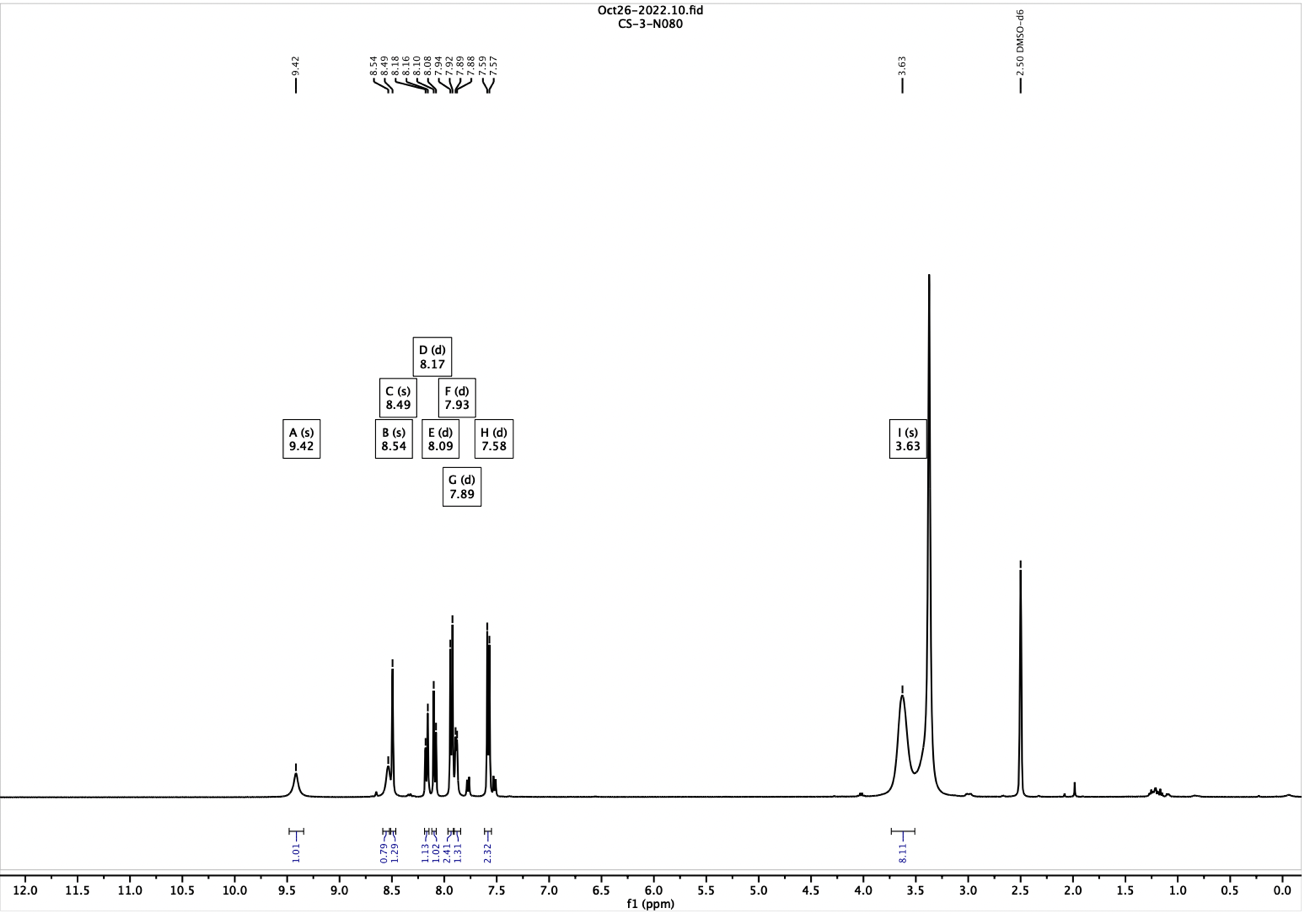
**

^1^H NMR spectrum of **7** in DMSO-*d*_6_ at 400 MHz.


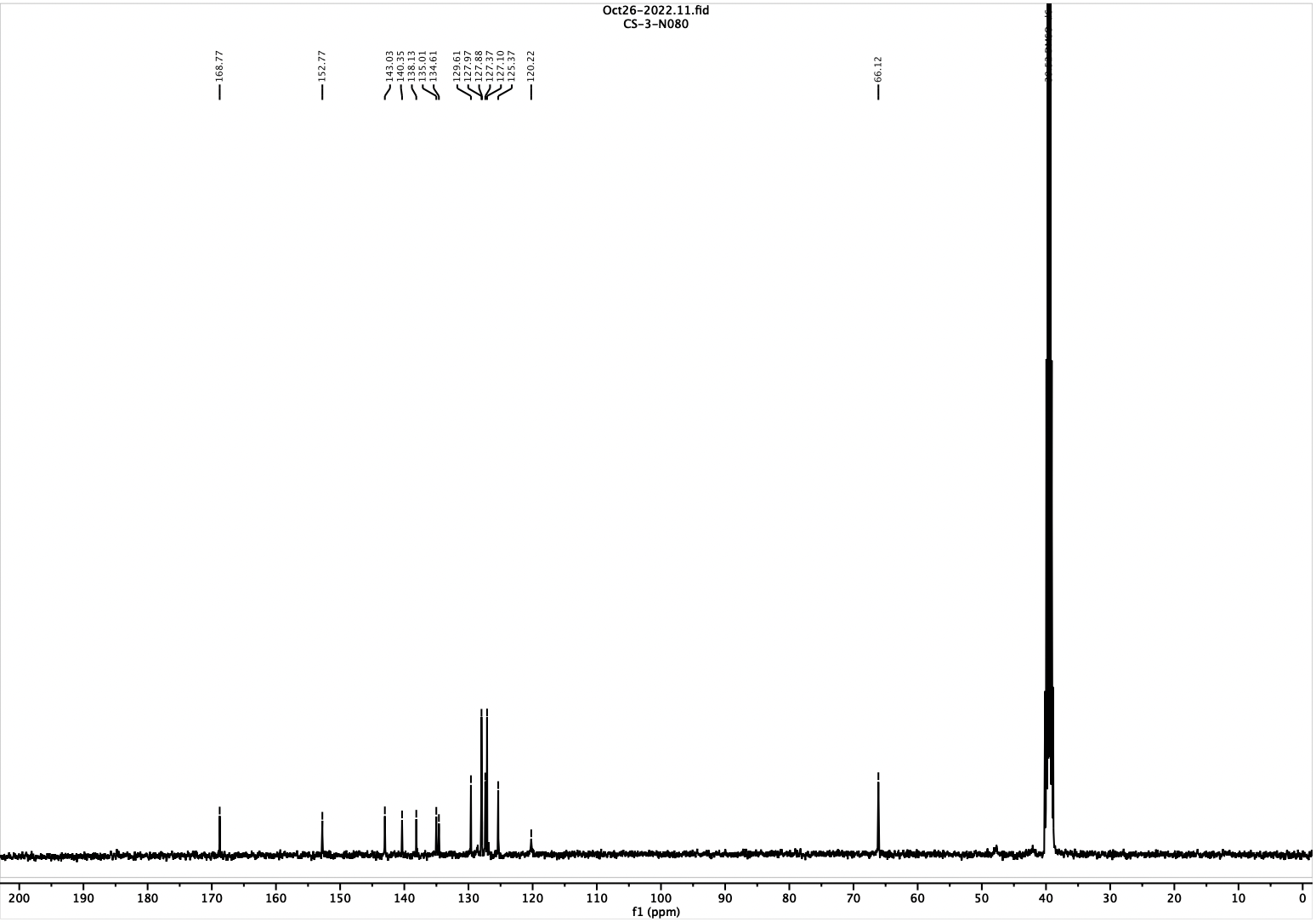


^13^C NMR spectrum of **7** in DMSO-*d*_6_ at 101 MHz.

**
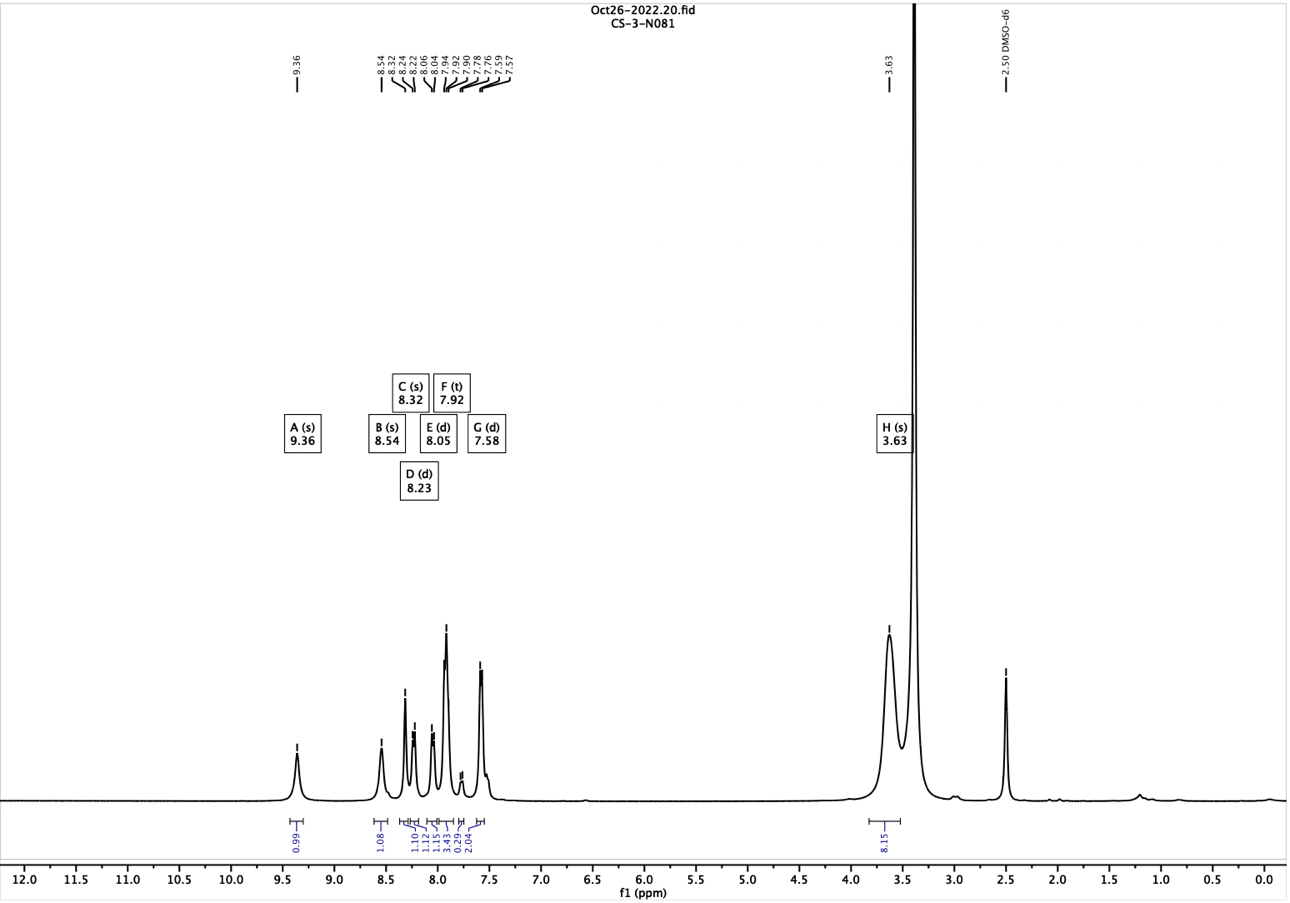
**

^1^H NMR spectrum of **8** in DMSO-*d*_6_ at 400 MHz.


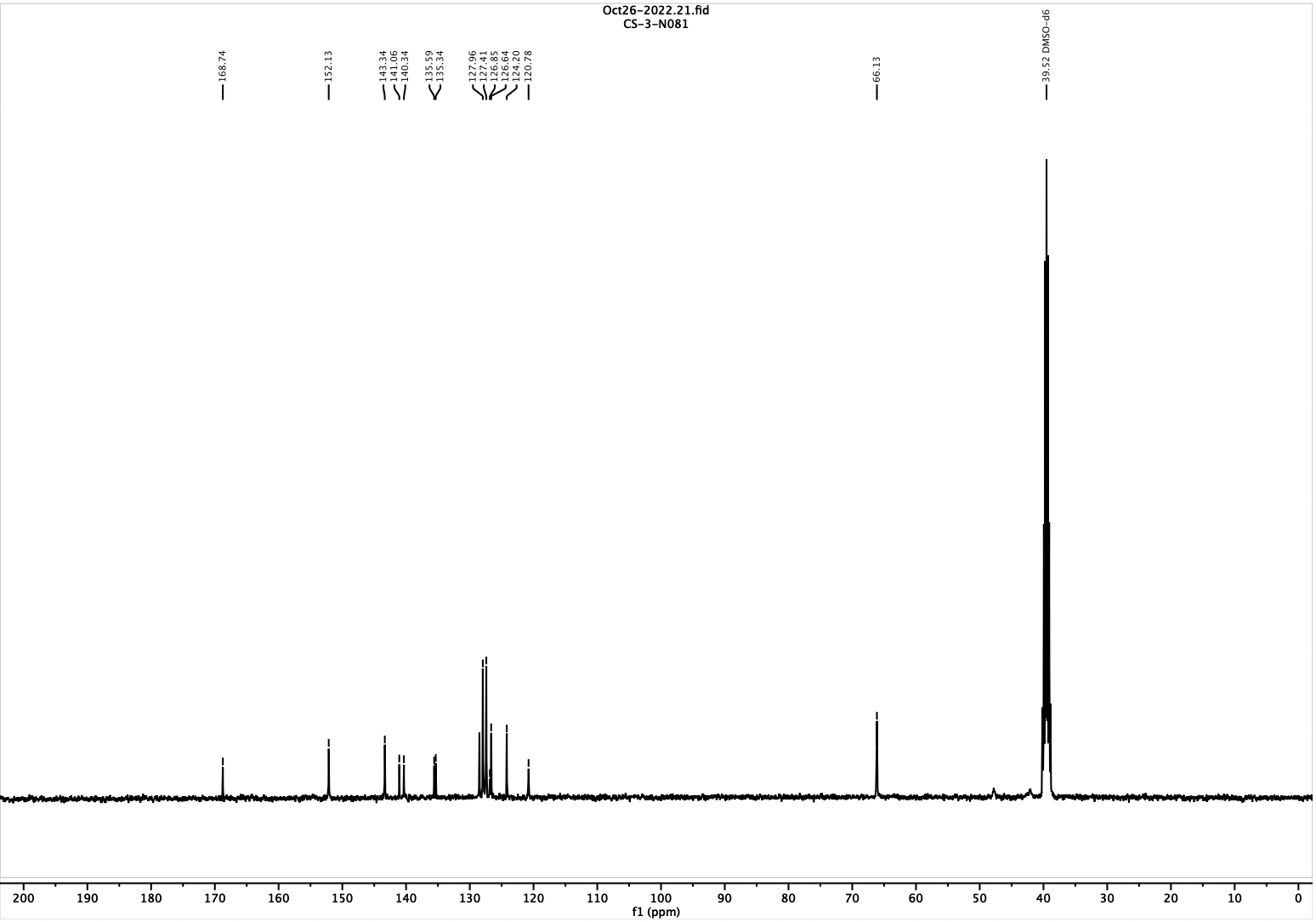


^13^C NMR spectrum of **8** in DMSO-*d*_6_ at 101 MHz.


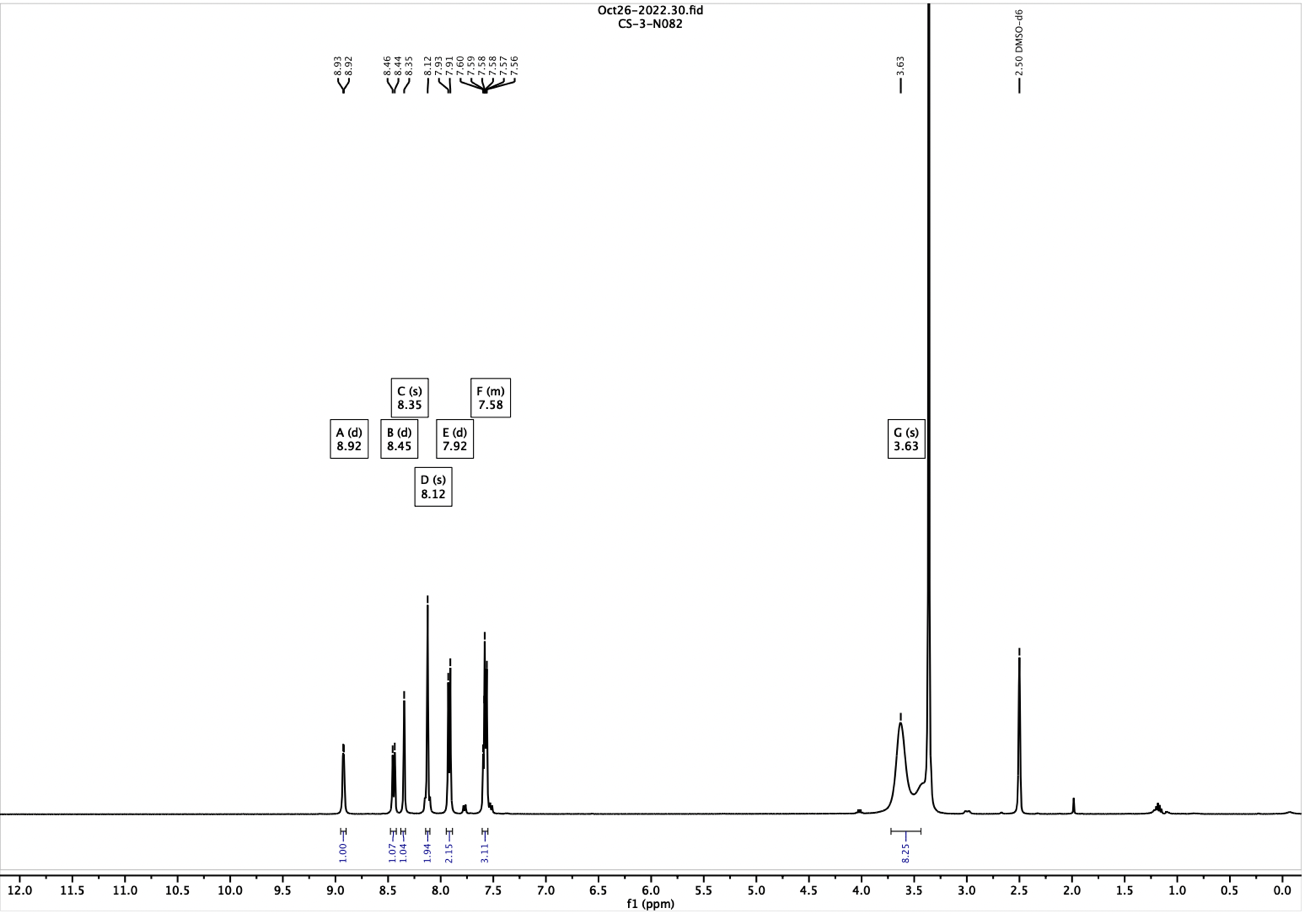


^1^H NMR spectrum of **9** in DMSO-*d*_6_ at 400 MHz.


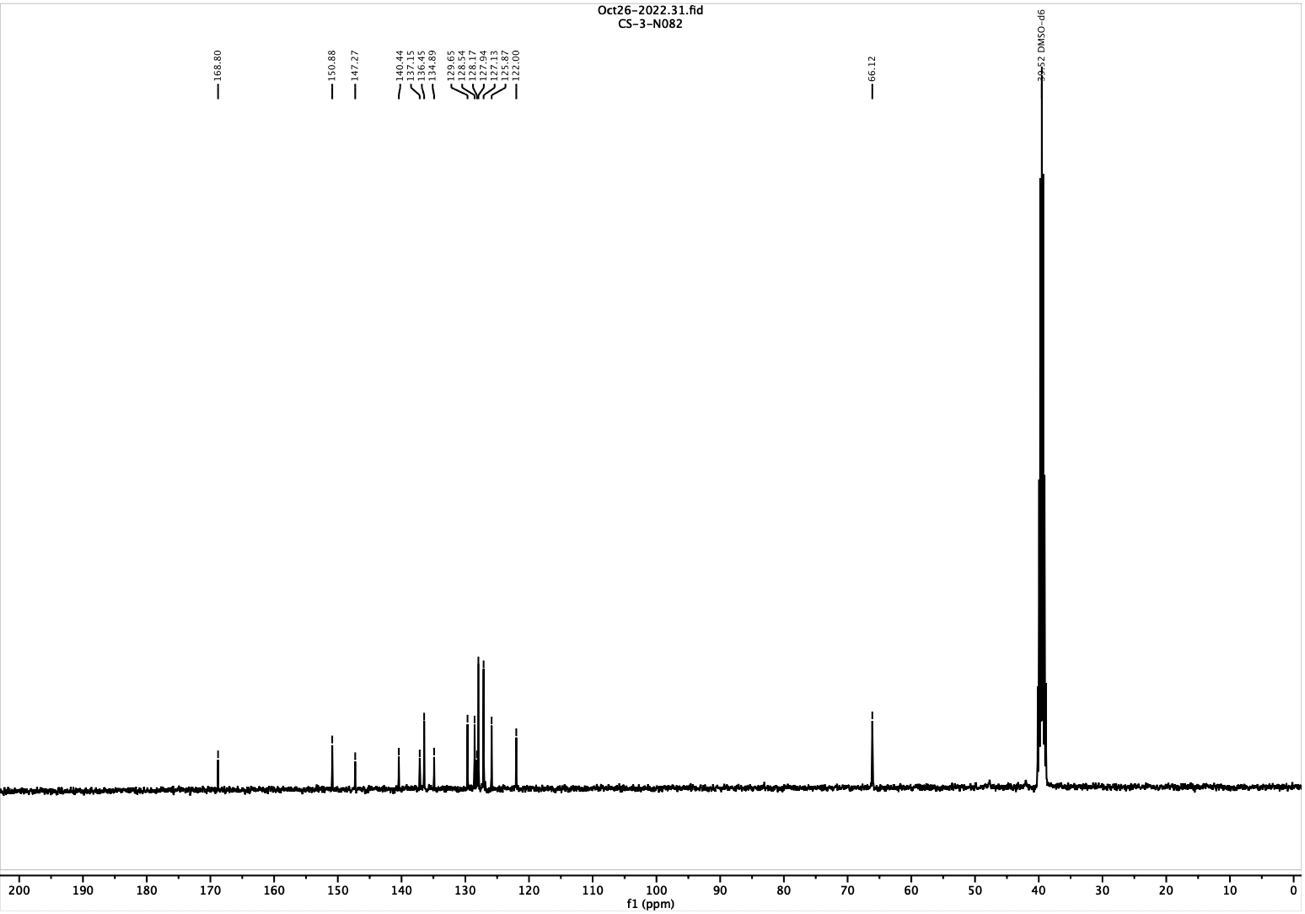


^13^C NMR spectrum of **9** in DMSO-*d*_6_ at 101 MHz.


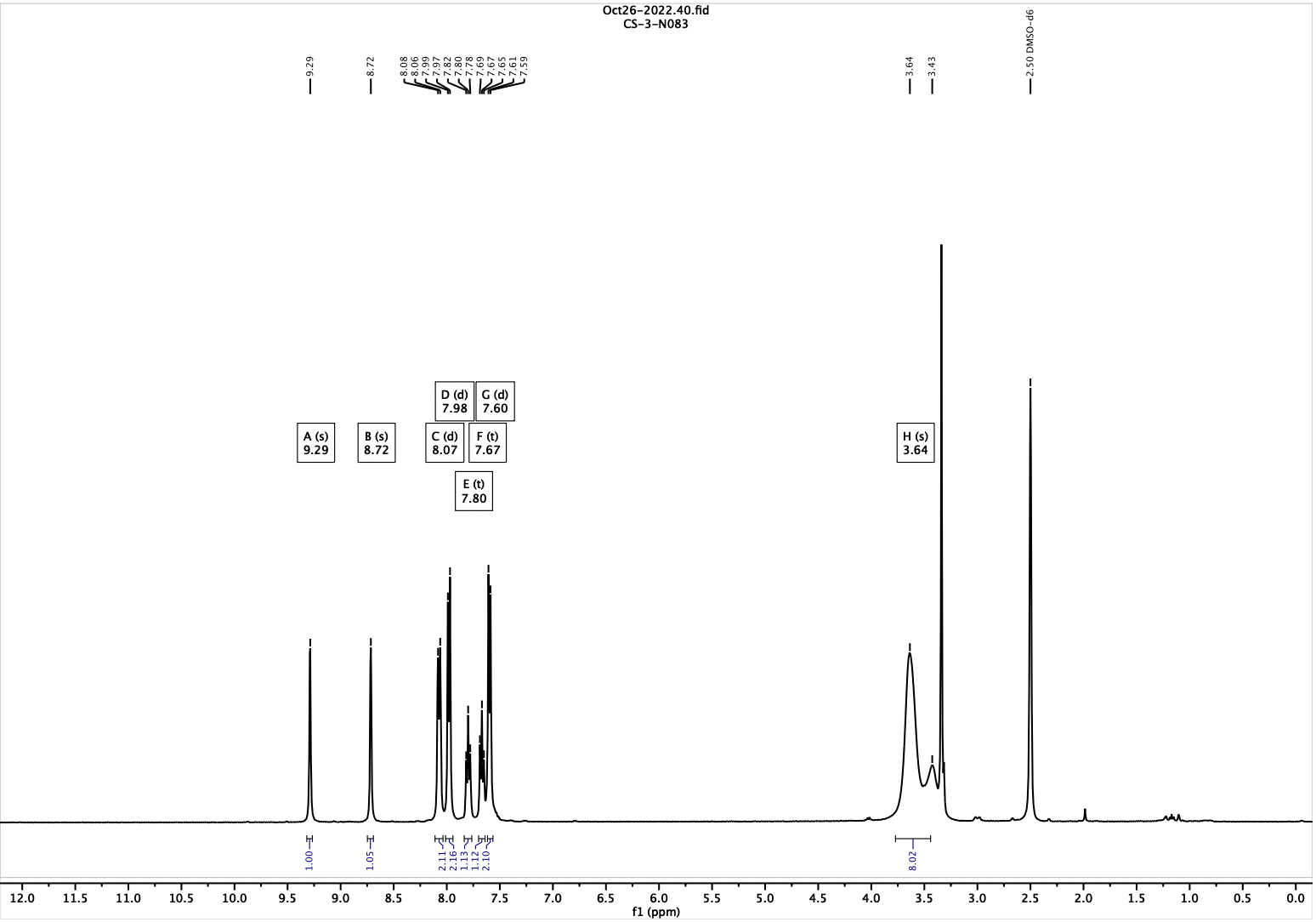


^1^H NMR spectrum of **10** in DMSO-*d*_6_ at 400 MHz.


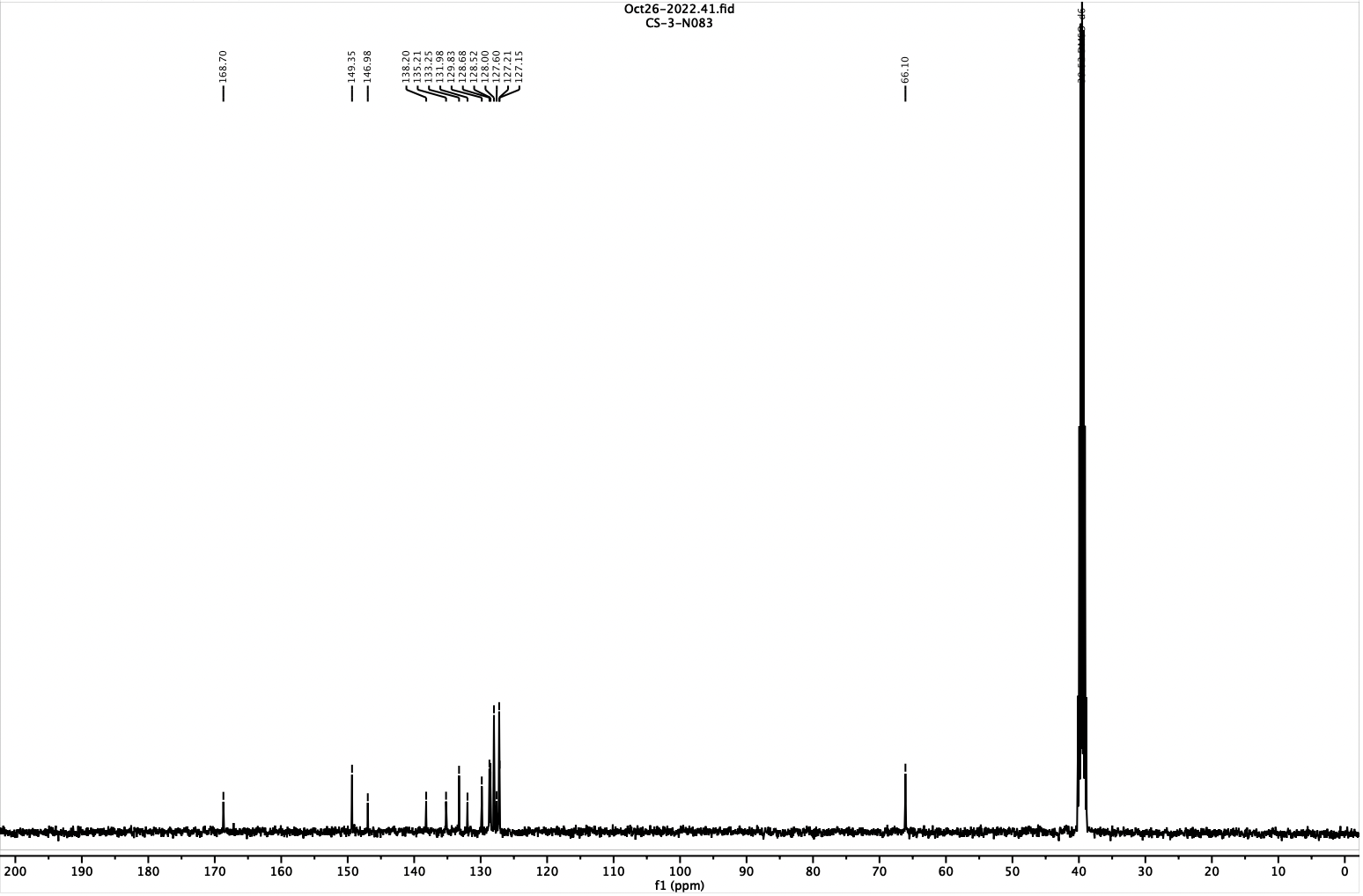


^13^C NMR spectrum of **10** in DMSO-*d*_6_ at 101 MHz.

**
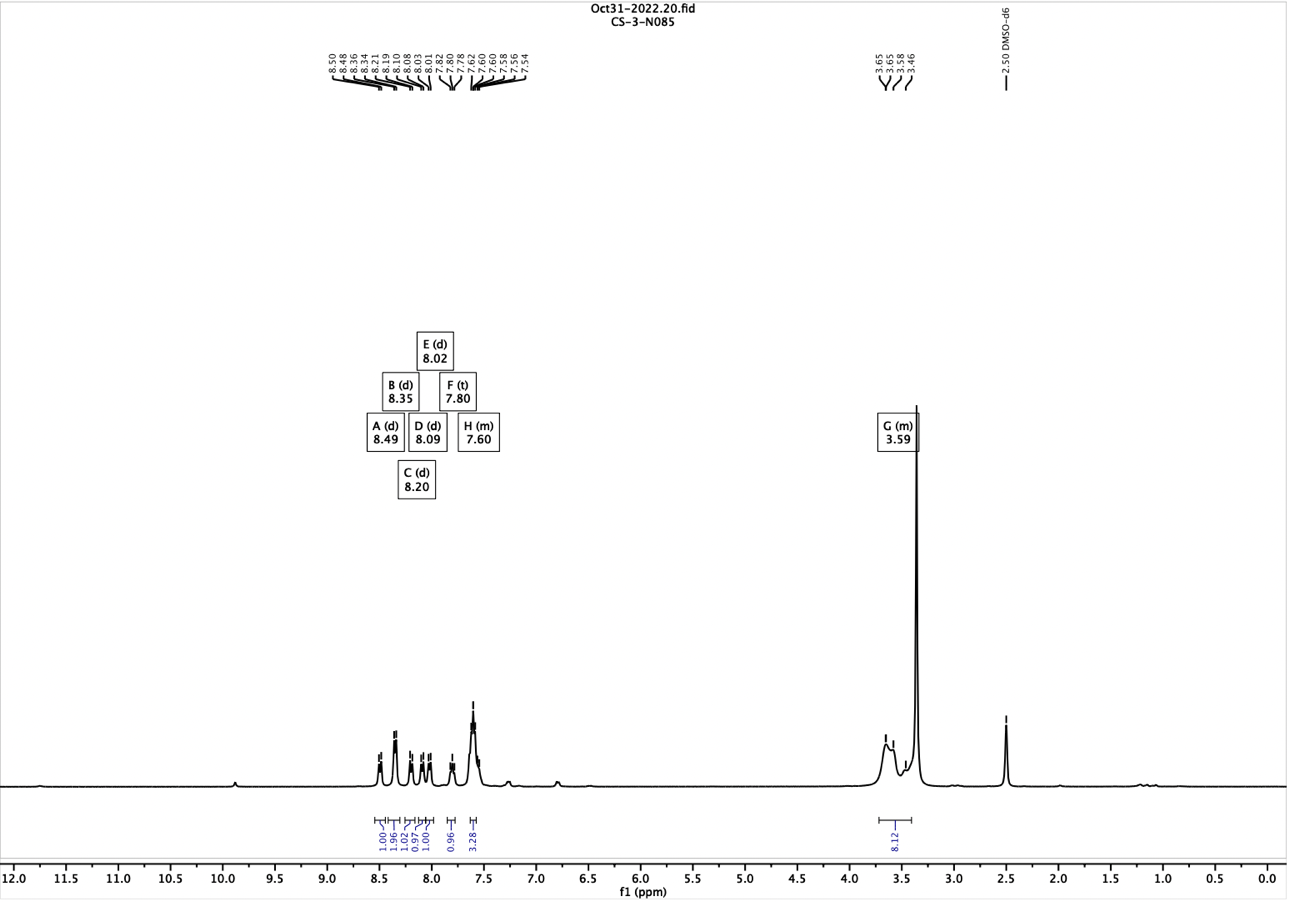
**

^1^H NMR spectrum of **11** in DMSO-*d*_6_ at 400 MHz.


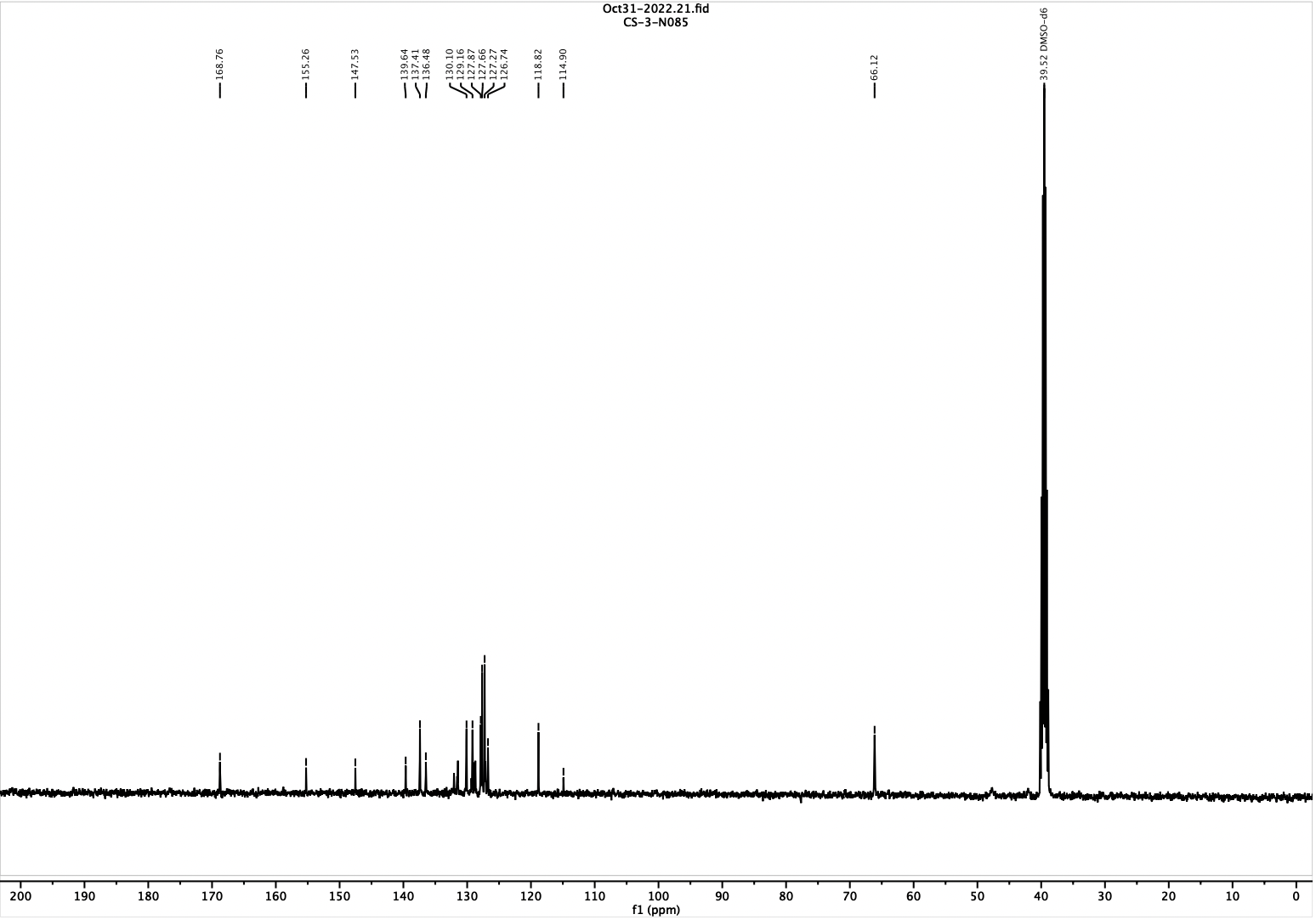


^13^C NMR spectrum of **11** in DMSO-*d*_6_ at 101 MHz.

**
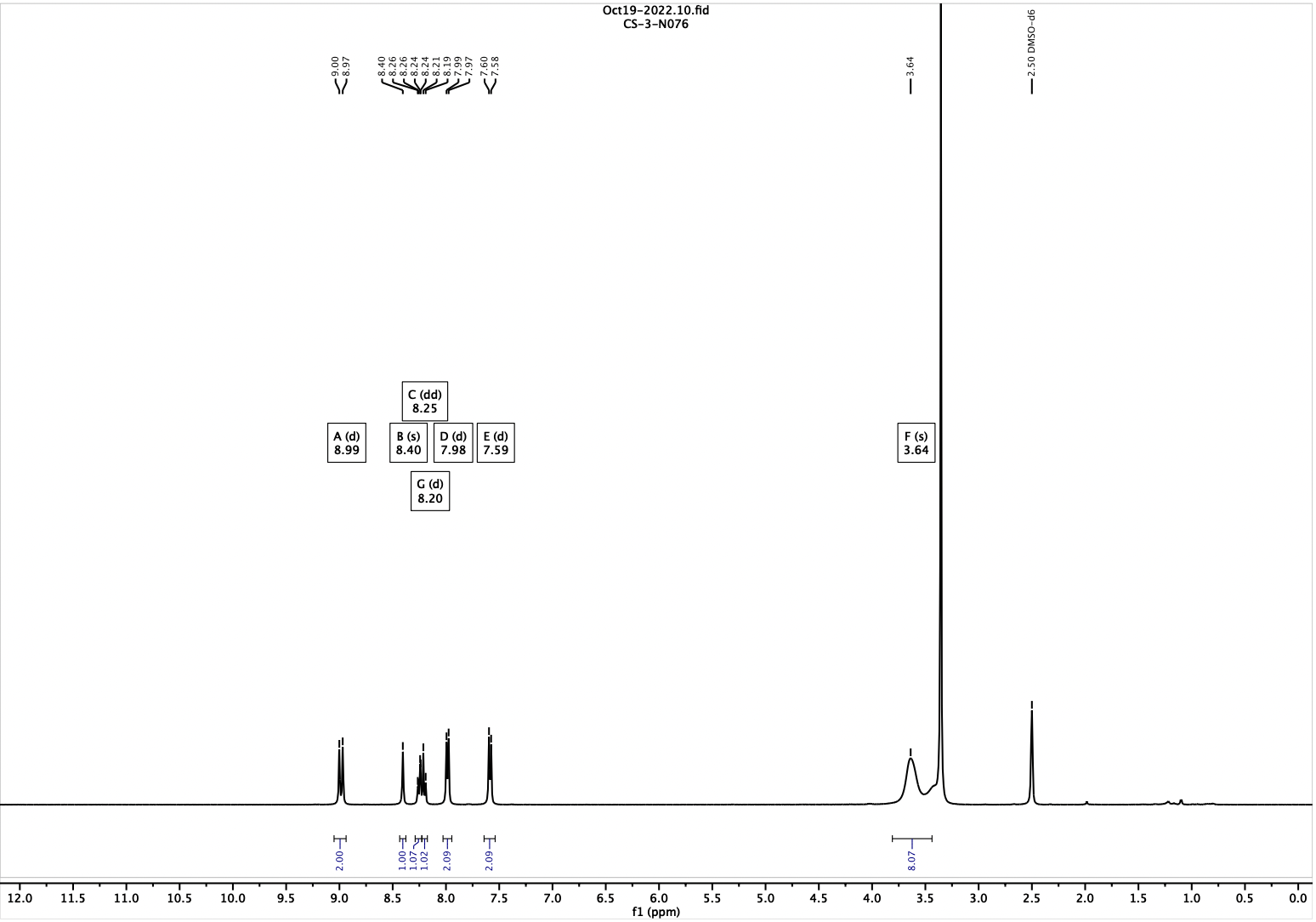
**

^1^H NMR spectrum of **12** in DMSO-*d*_6_ at 400 MHz.


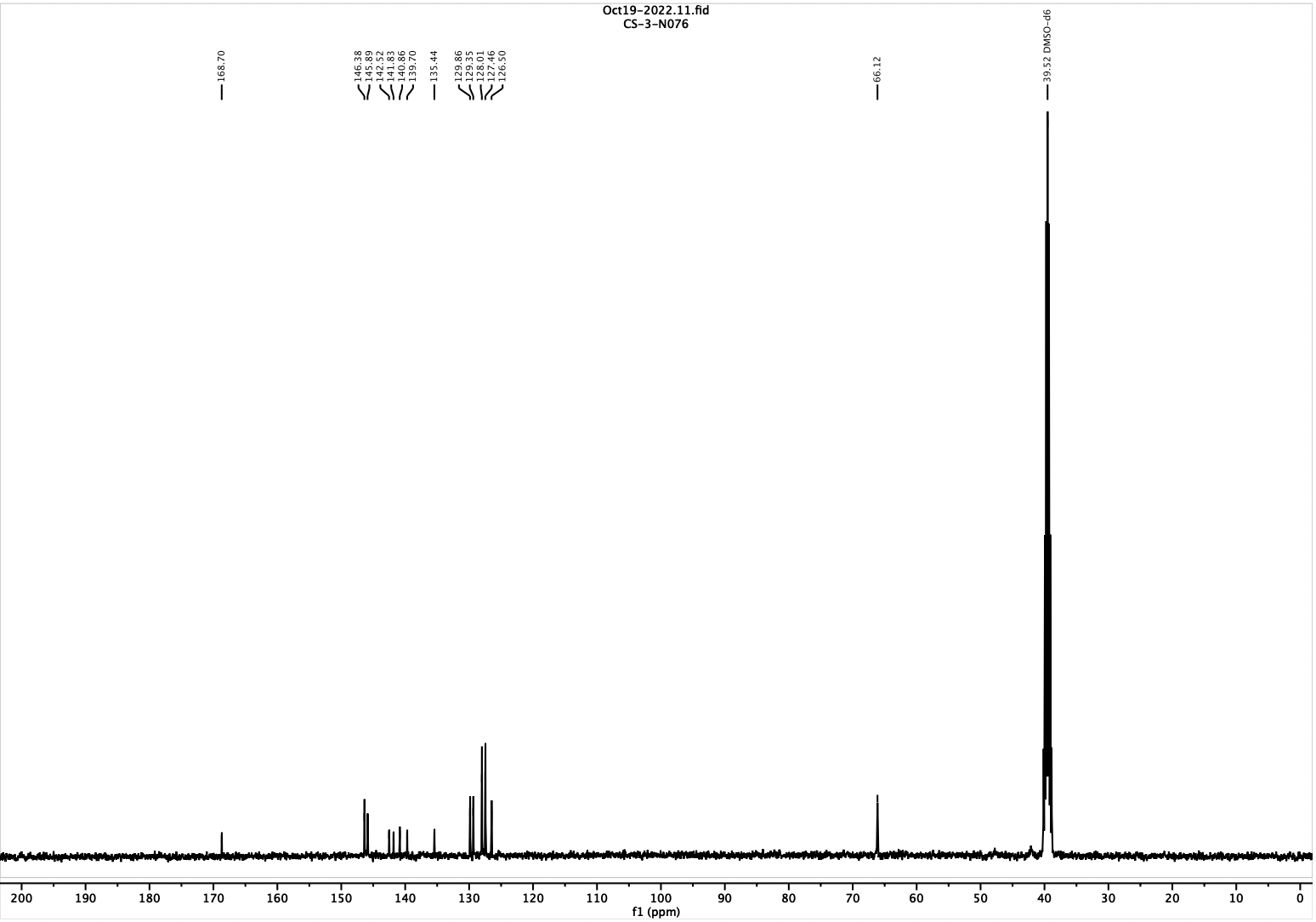


^13^C NMR spectrum of **12** in DMSO-*d*_6_ at 101 MHz.

**
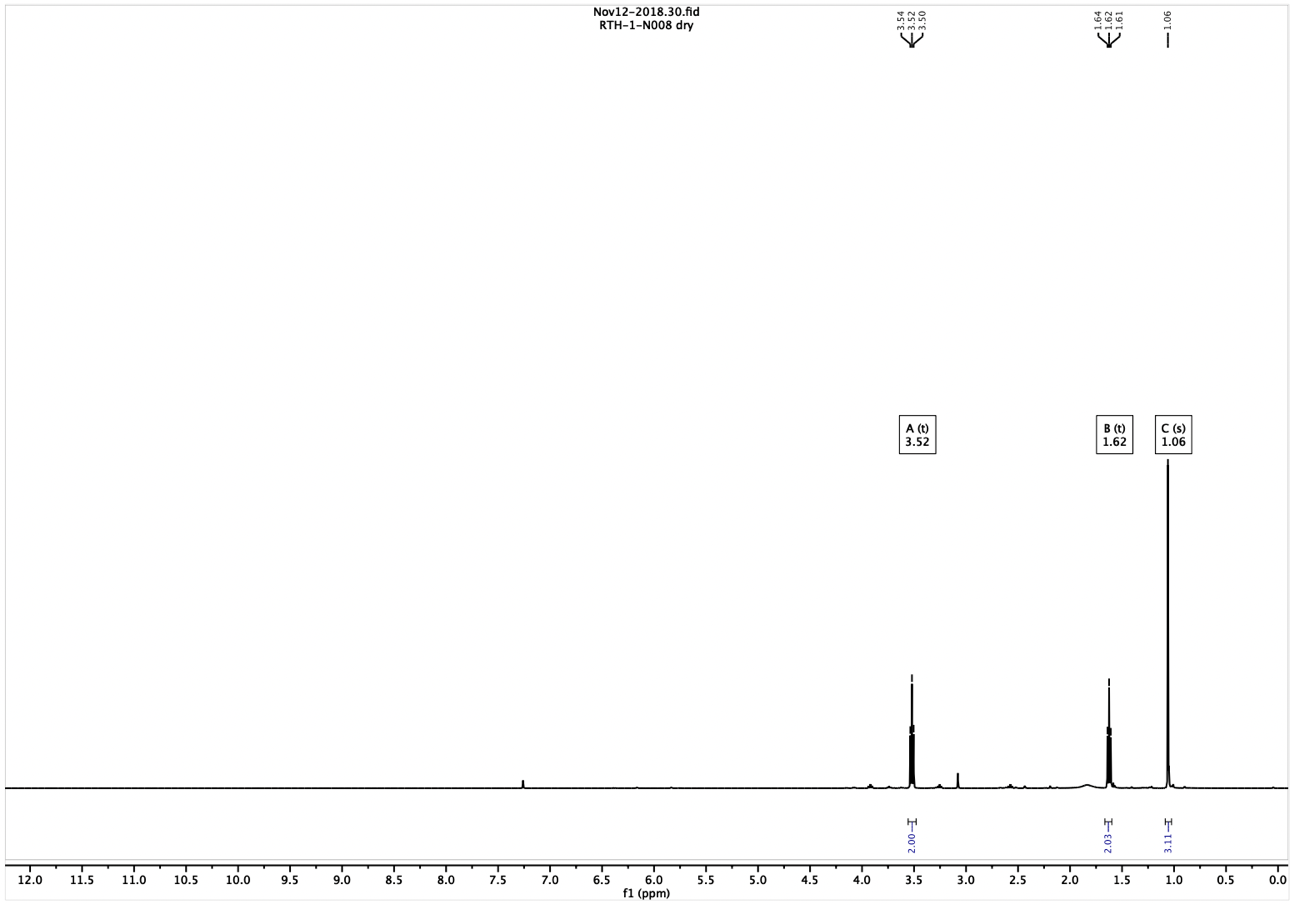
**

^1^H NMR spectrum of **18** in CDCl_3_ at 400 MHz.

^
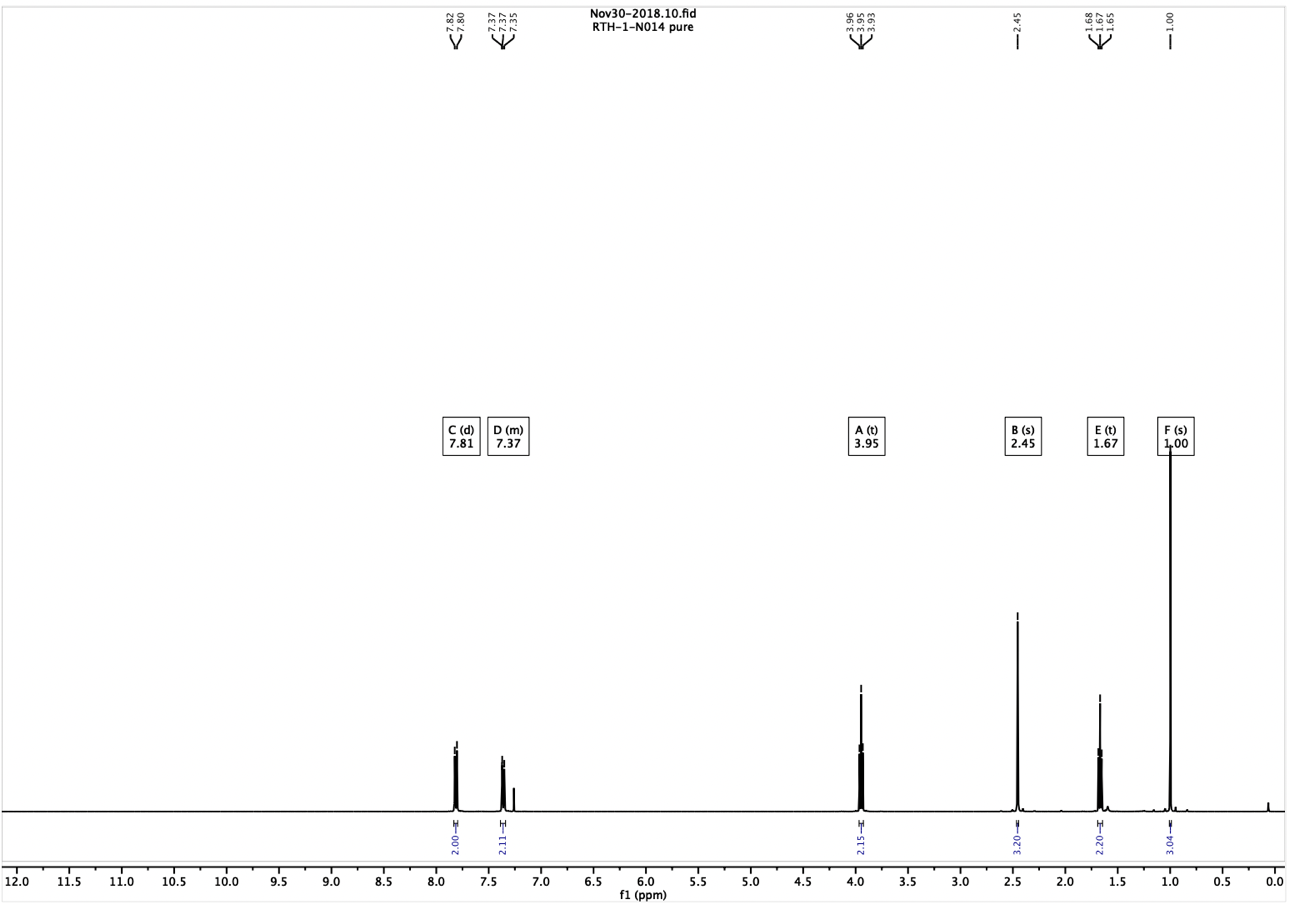
^

^1^H NMR spectrum of **19** in CDCl_3_ at 400 MHz.

**
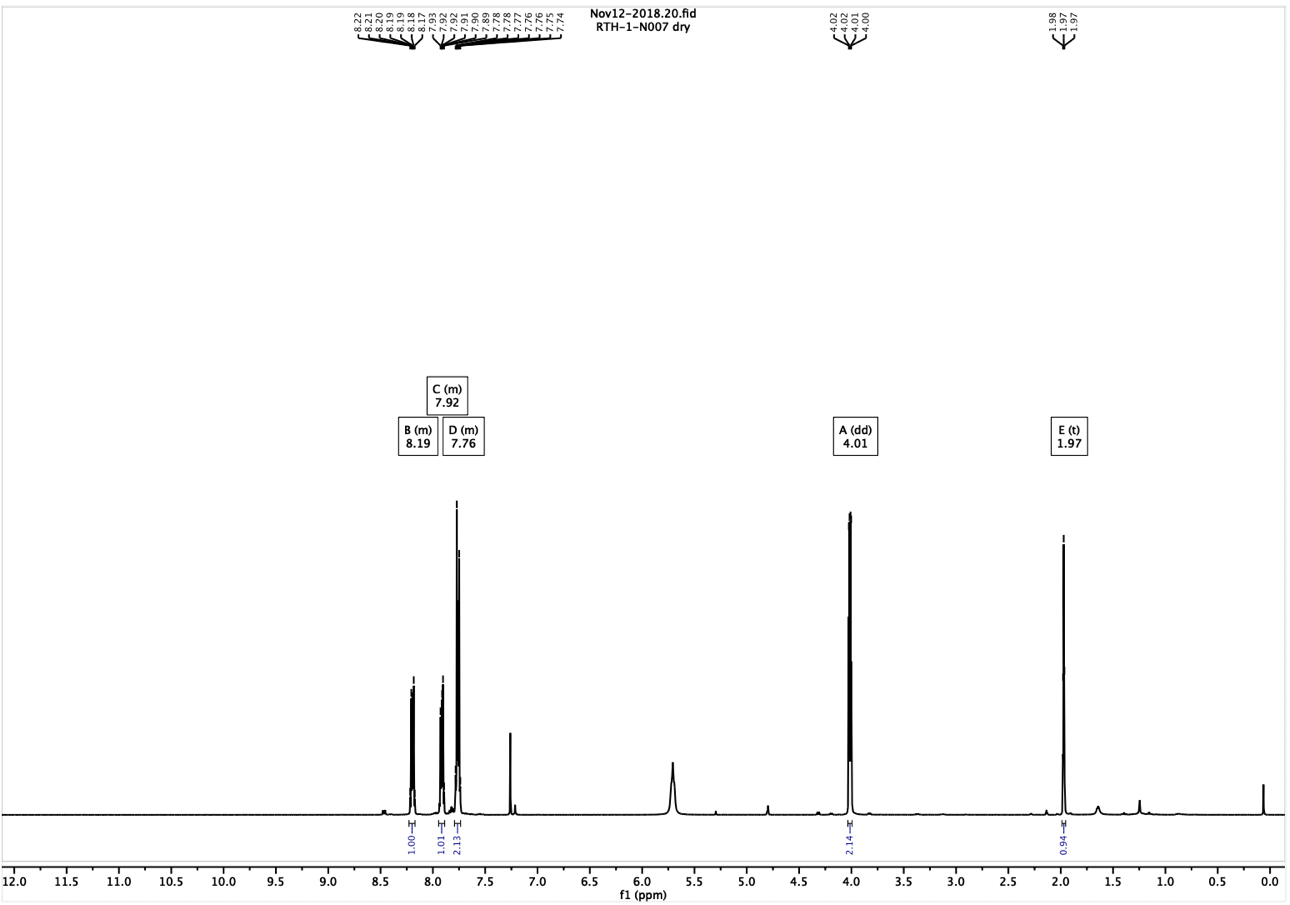
**

^1^H NMR spectrum of **21** in CDCl_3_ at 400 MHz.

**
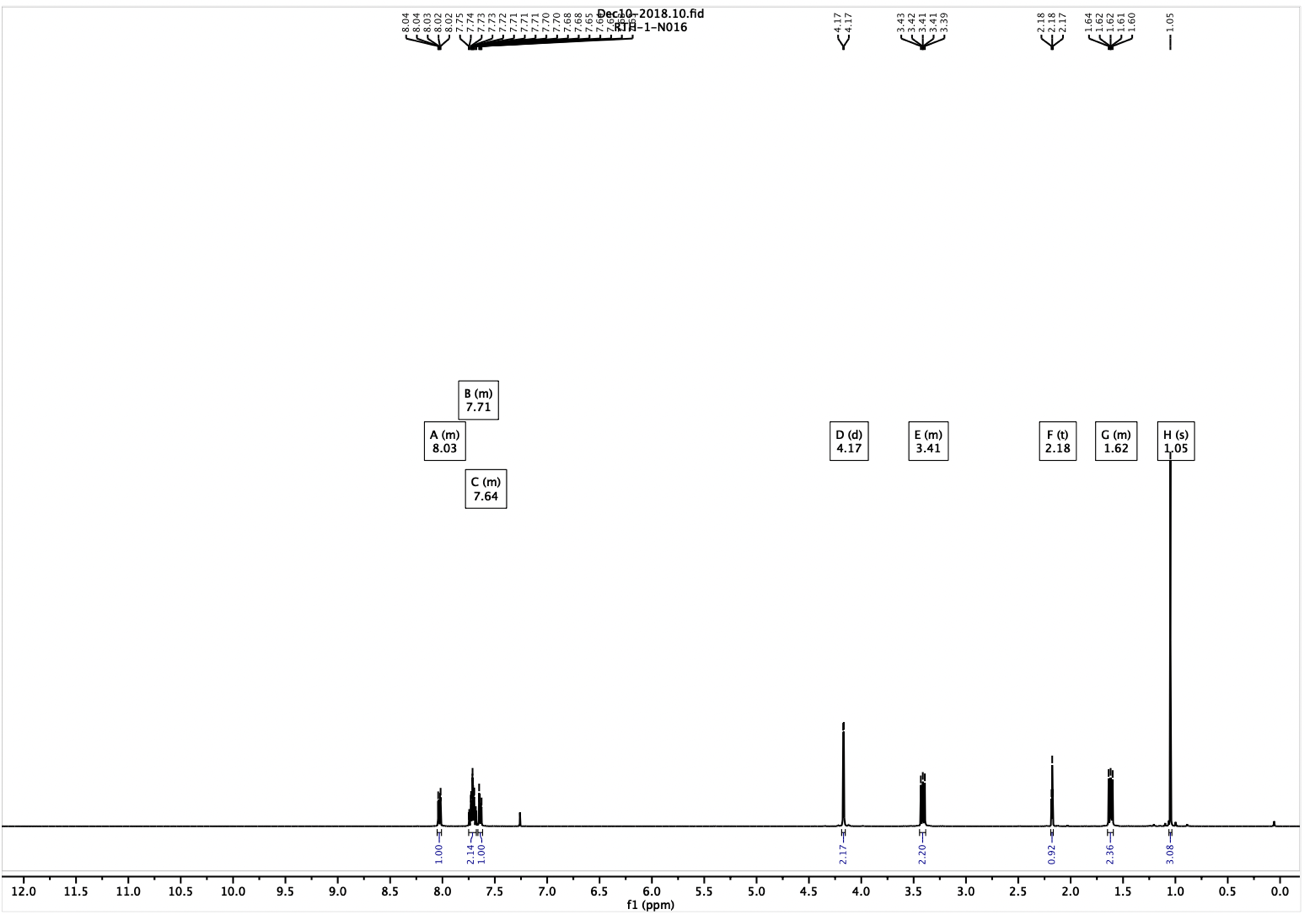
**

^1^H NMR spectrum of **22** in CDCl_3_ at 400 MHz.

**
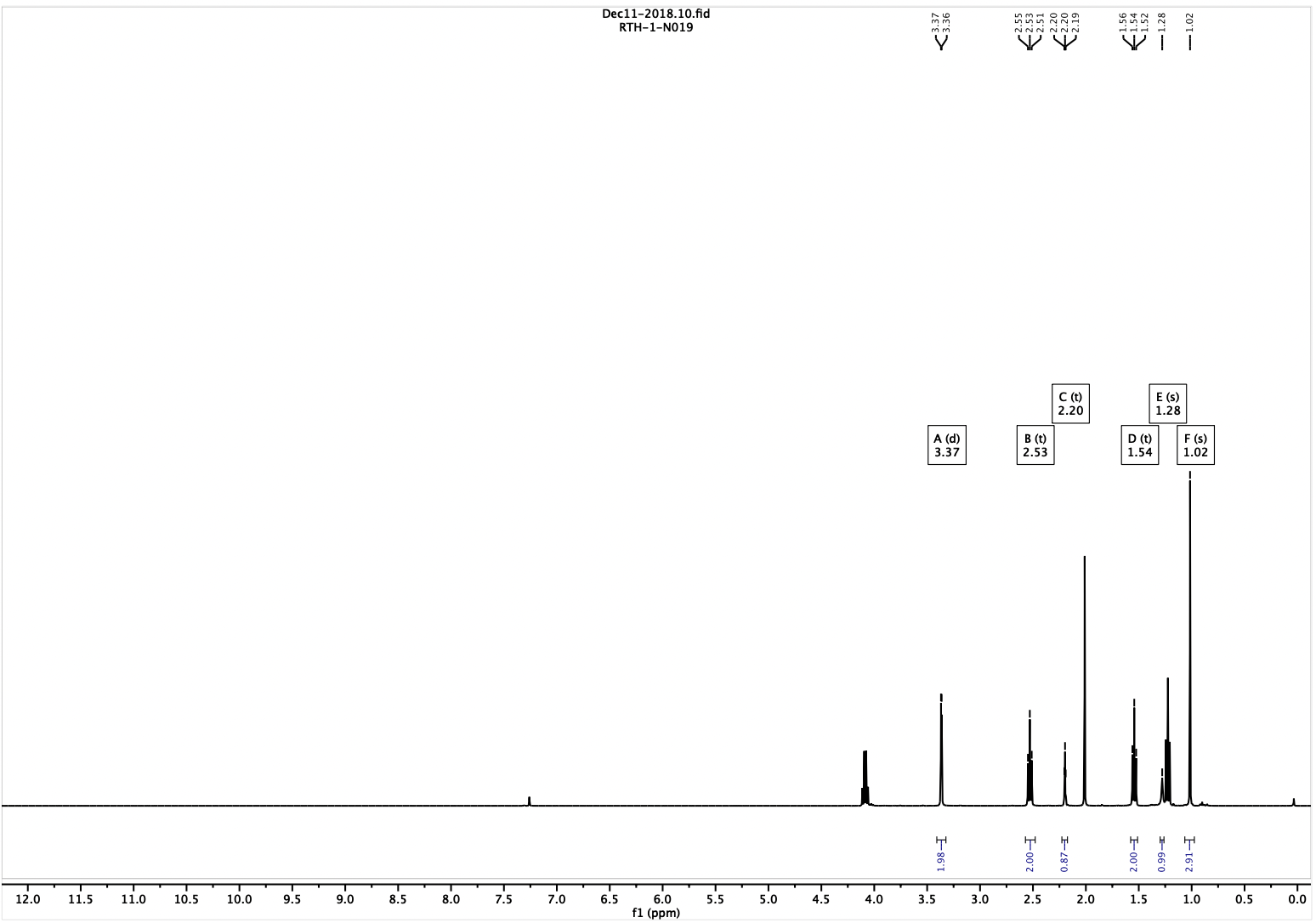
**

^1^H NMR spectrum of **23** in CDCl_3_ at 400 MHz.

**
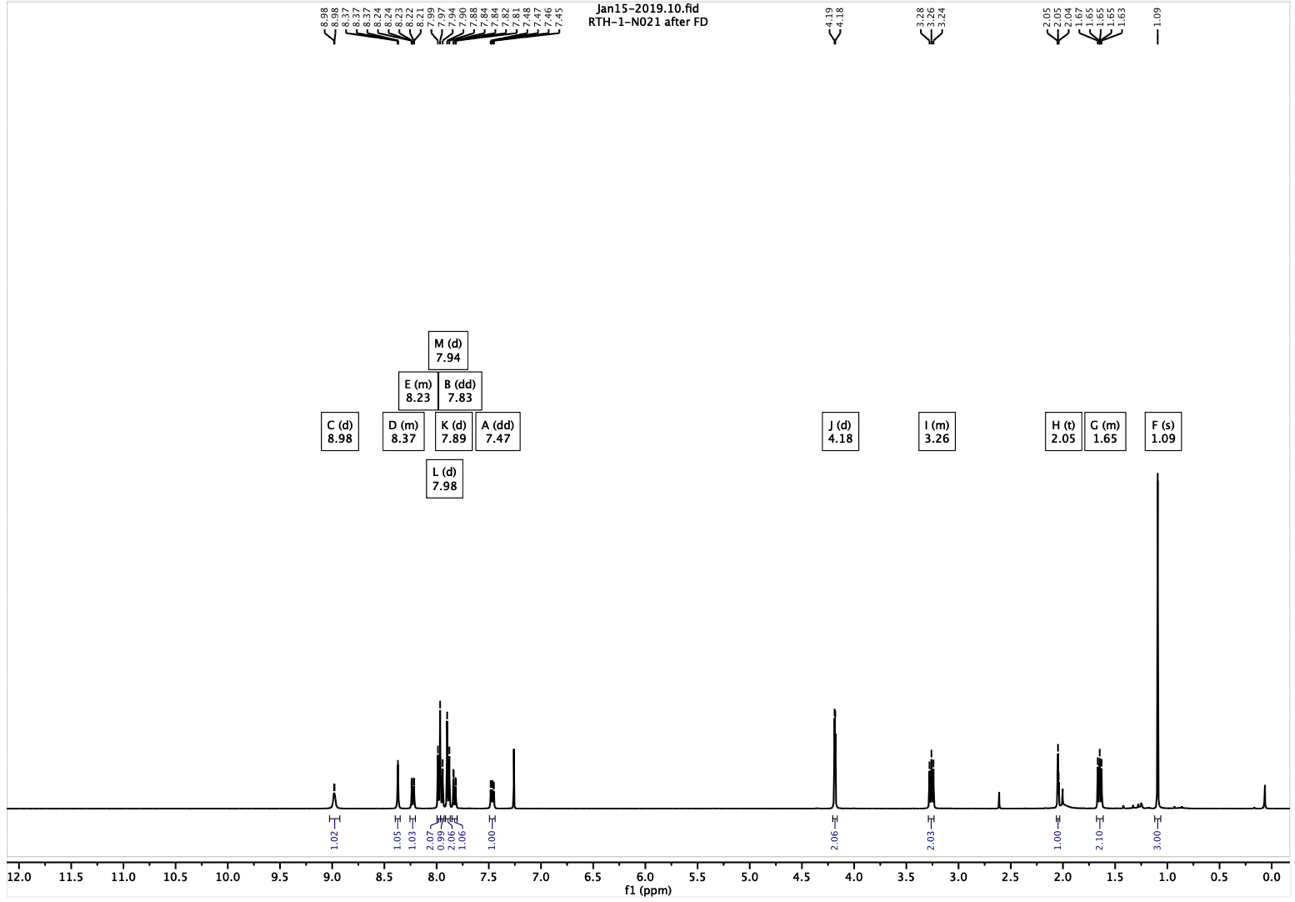
**

^1^H NMR spectrum of **7PQYnD** in CDCl_3_ at 400 MHz.


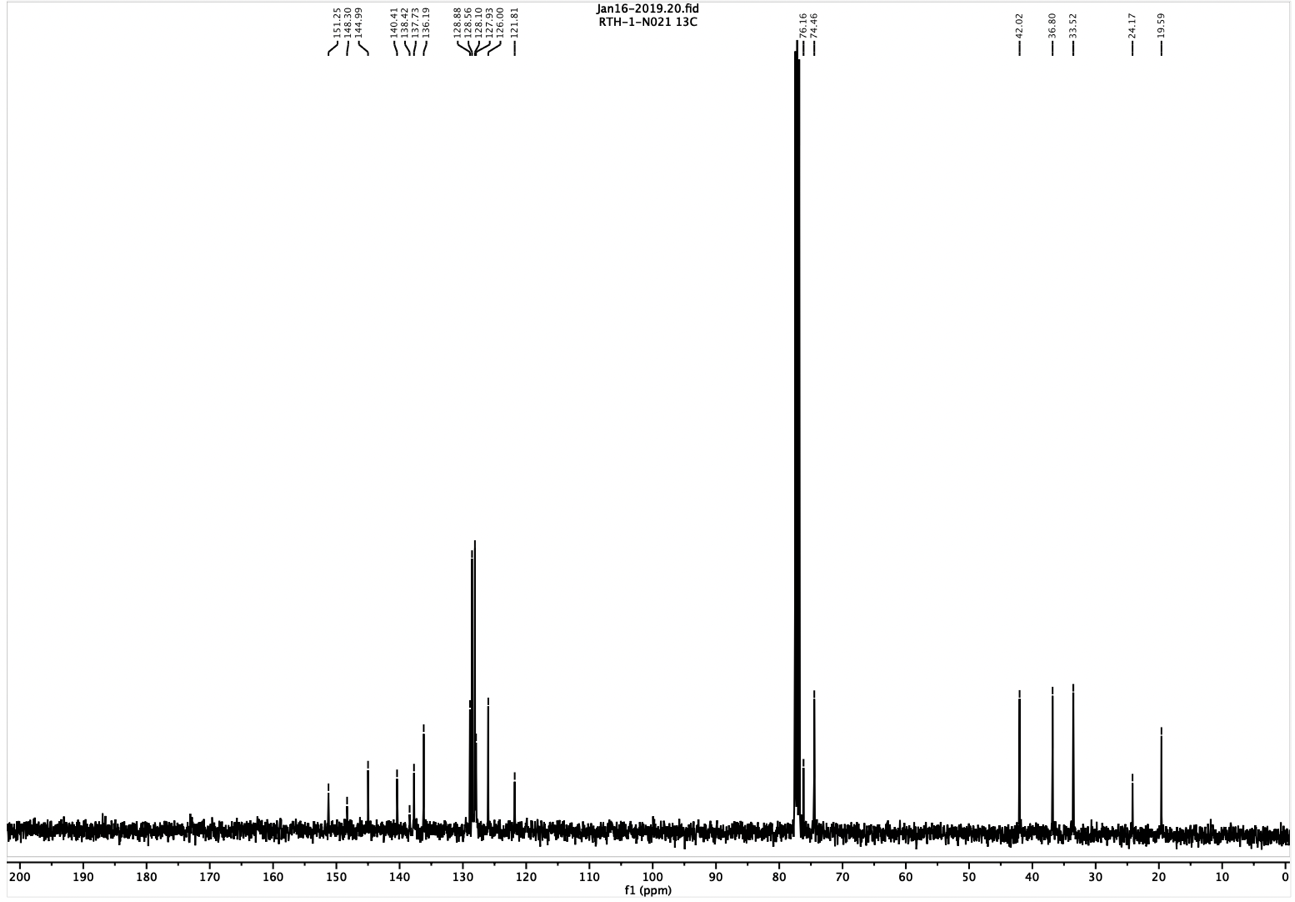


^13^C NMR spectrum of **7PQYnD** in CDCl_3_ at 101 MHz.

**
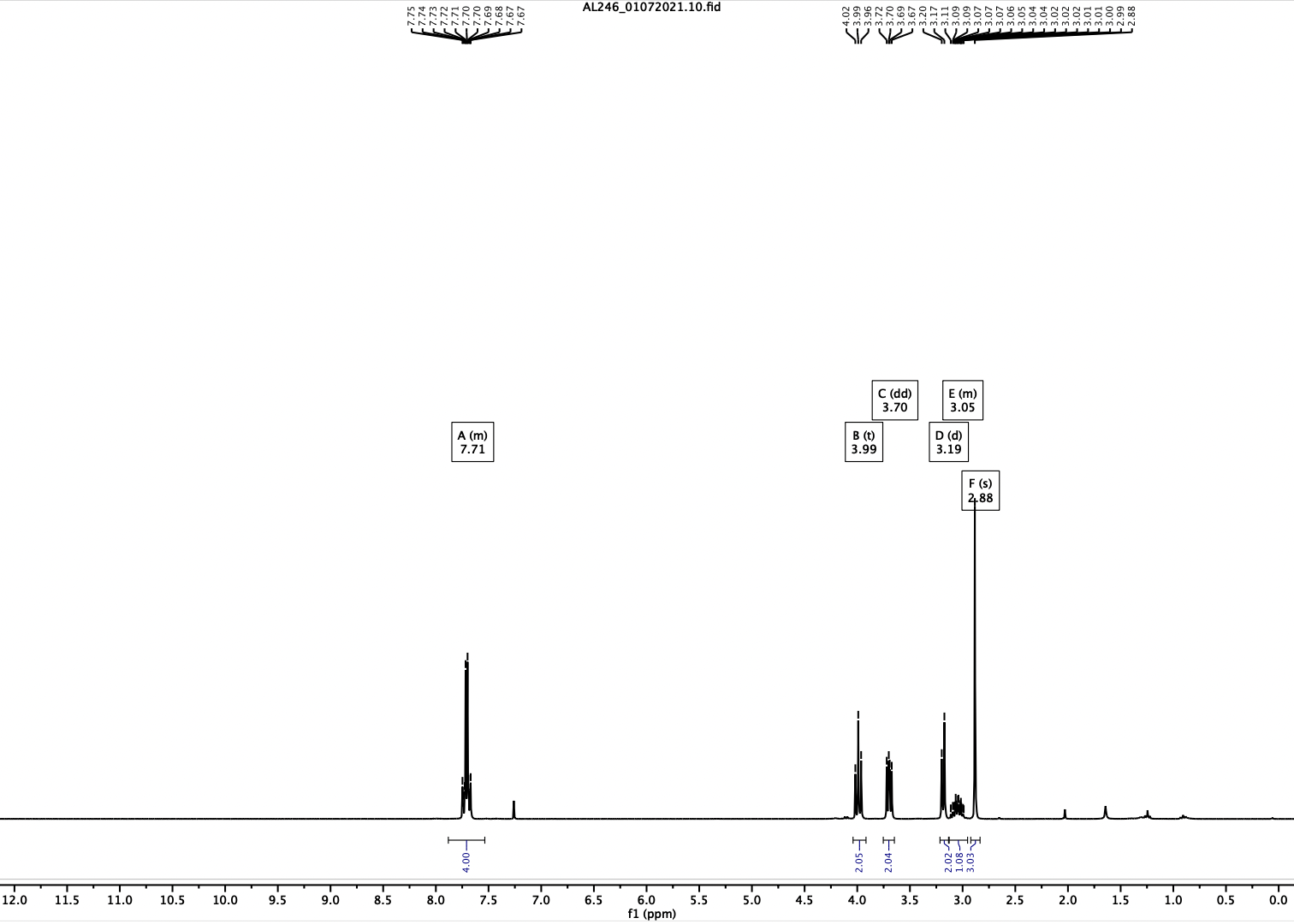
**

^1^H NMR spectrum of **26** in CDCl_3_ at 300 MHz.

**
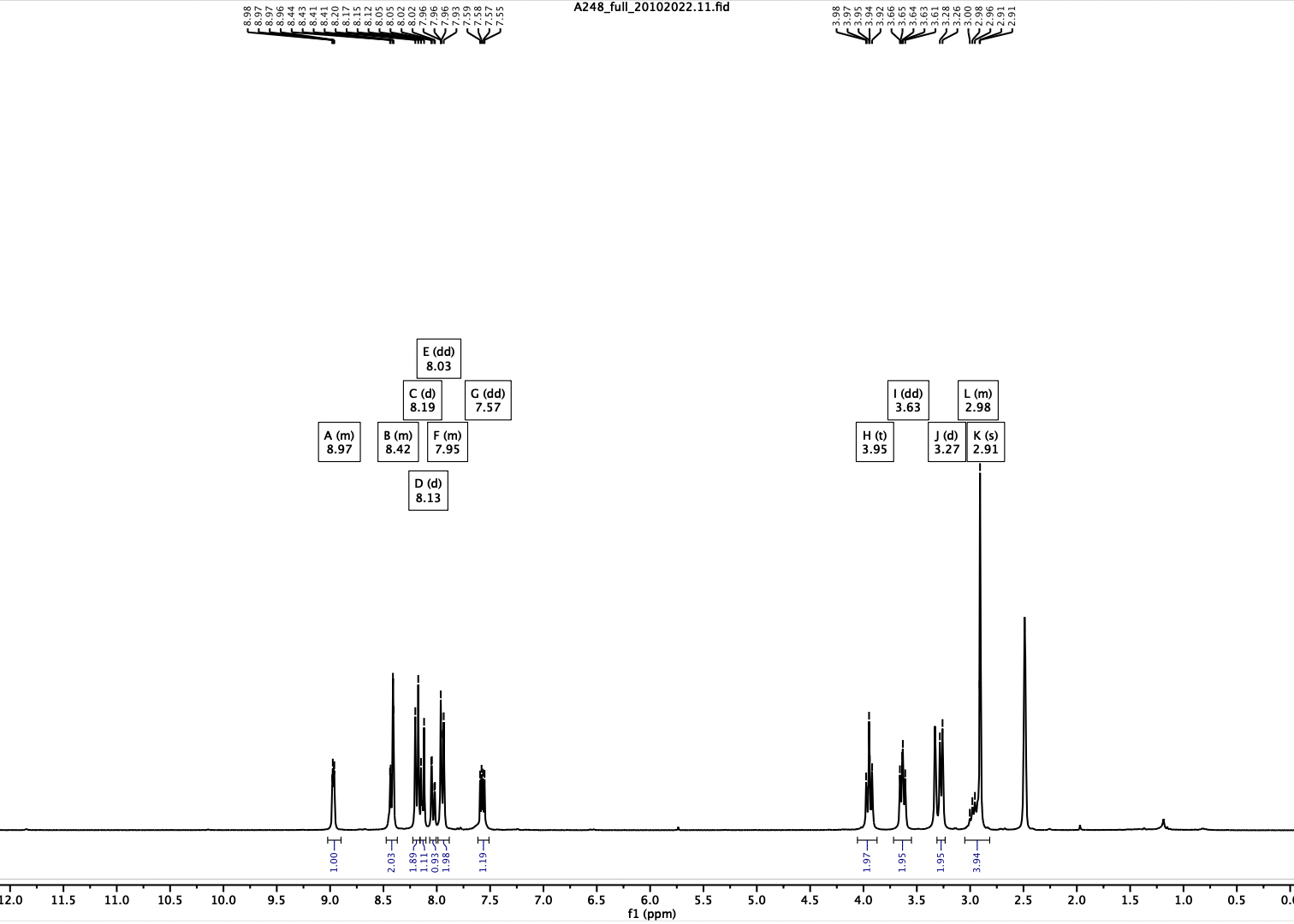
**

^1^H NMR spectrum of **AZ'320** in DMSO-*d*_6_ at 300 MHz.


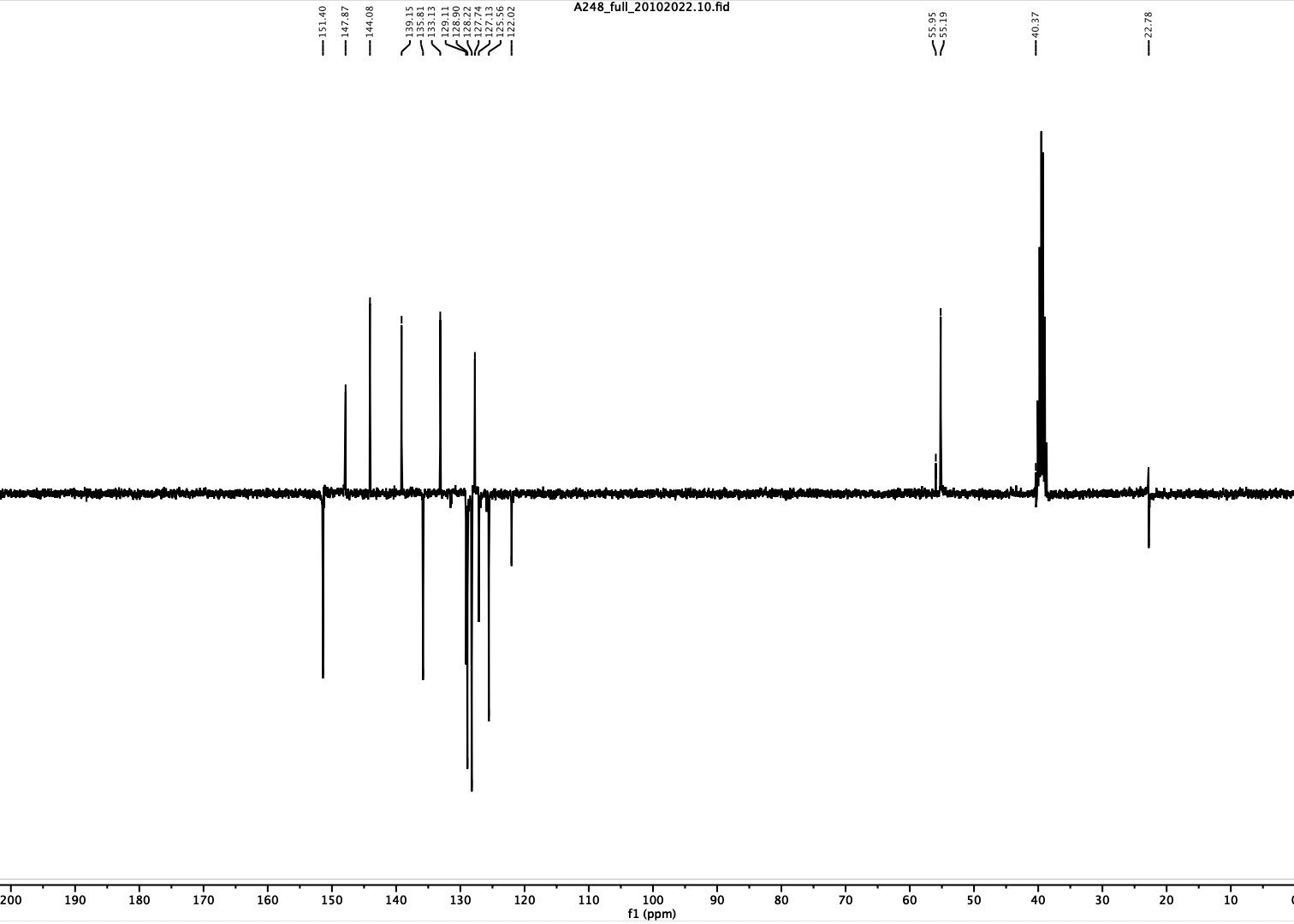


^13^C NMR spectrum of **AZ'320** in DMSO-*d*_6_ at 75 MHz.
